# The pharmacodynamics-based prophylactic benefits of GLP-1 receptor agonists and SGLT2 inhibitors on neurodegenerative diseases: evidence from a network meta-analysis

**DOI:** 10.1186/s12916-025-04018-w

**Published:** 2025-04-07

**Authors:** Ping-Tao Tseng, Bing-Yan Zeng, Chih-Wei Hsu, Chao-Ming Hung, Andre F. Carvalho, Brendon Stubbs, Yen-Wen Chen, Tien-Yu Chen, Wei-Te Lei, Jiann-Jy Chen, Kuan-Pin Su, Yow-Ling Shiue, Chih-Sung Liang

**Affiliations:** 1 Institute of Precision Medicine, National Sun Yat-Sen University, 70 Lienhai Rd, Kaohsiung City, 80424 Taiwan; 2 Institute of Biomedical Sciences, National Sun Yat-Sen University, Kaohsiung, Taiwan; 3 Prospect Clinic for Otorhinolaryngology & Neurology, No. 252, Nanzixin Road, Nanzi District, Kaohsiung City, 81166 Taiwan; 4 Department of Internal Medicine, E-Da Dachang Hospital, I-Shou University, Kaohsiung, Taiwan; 5 Department of Psychiatry, Kaohsiung Chang Gung Memorial Hospital and Chang Gung University College of Medicine, Kaohsiung, Taiwan; 6 Division of General Surgery, Department of Surgery, E-Da Cancer Hospital, I-Shou University, Kaohsiung, Taiwan; 7 School of Medicine, College of Medicine, I-Shou University, Kaohsiung, Taiwan; 8 Innovation in Mental and Physical Health and Clinical Treatment (IMPACT) Strategic Research Centre, School of Medicine, Barwon Health, Deakin University, Geelong, VIC Australia; 9 Department of Psychological Medicine, Institute of Psychiatry, Psychology and Neuroscience, King’s College London, London, UK; 10 Department of Sport, University of Vienna, Vienna, Austria; 11 Department of Psychiatry, Tri-Service General Hospital; School of Medicine, National Defense Medical Center, Taipei, Taiwan; 12 Institute of Brain Science, National Yang Ming Chiao Tung University, Taipei, 112 Taiwan; 13 Section of Immunology, Rheumatology, and Allergy Department of Pediatrics, Section of Immunology, Rheumatology, and Allergy Department of Pediatrics, Hsinchu Munipical MacKay Children’s Hospital, Hsinchu City, Taiwan; 14 Center for Molecular and Clinical Immunology, Chang Gung University, Taoyuan, Taiwan; 15 Department of Otorhinolaryngology, E-Da Cancer Hospital, I-Shou University, Kaohsiung, Taiwan; 16 Department of Psychiatry & Mind-Body Interface Laboratory (MBI-Lab), China Medical University Hospital, Taichung, Taiwan; 17 College of Medicine, China Medical University, Taichung, Taiwan; 18 An-Nan Hospital, China Medical University, Tainan, Taiwan; 19 Department of Psychiatry, Tri-Service General Hospital; School of Medicine, National Defense Medical Center, Beitou District, Beitou Branch, No. 60, Xinmin Road, Taipei City, 112 Taiwan; 20 Department of Psychiatry, National Defense Medical Center, Taipei, Taiwan

**Keywords:** Network meta-analysis, GLP-1 receptor agonist, SGLT2 inhibitor, Neurodegenerative disease, Parkinson’s disease

## Abstract

**Background:**

Glucagon-like peptide-1 (GLP-1) receptor agonists and sodium–glucose cotransporter 2 (SGLT2) inhibitors represent a new generation of antihyperglycemic agents that operate through mechanisms distinct from conventional diabetes treatments. Beyond their metabolic effects, these medications have demonstrated neuroprotective properties in preclinical studies. While clinical trials have explored their therapeutic potential in established neurodegenerative conditions, their role in disease prevention remains unclear. We conducted a network meta-analysis (NMA) to comprehensively evaluate the prophylactic benefits of these agents across multiple neurodegenerative diseases and identify the most promising preventive strategies.

**Methods:**

We systematically searched PubMed, Embase, ClinicalKey, Cochrane CENTRAL, ProQuest, ScienceDirect, Web of Science, and ClinicalTrials.gov through October 24th, 2024, for randomized controlled trials (RCTs) of GLP-1 receptor agonists or SGLT2 inhibitors. Our primary outcome was the incidence of seven major neurodegenerative diseases: Parkinson’s disease, Alzheimer’s disease, Lewy body dementia, multiple sclerosis, amyotrophic lateral sclerosis, frontotemporal dementia, and Huntington’s disease. Secondary outcomes included safety profiles assessed through dropout rates. We performed a frequentist-based NMA and evaluated risk of bias with Risk of Bias tool. The main result of the primary outcome in the current study would be re-affirmed via sensitivity test with Bayesian-based NMA.

**Results:**

Our analysis encompassed 22 RCTs involving 138,282 participants (mean age 64.8 years, 36.4% female). Among all investigated medications, only dapagliflozin demonstrated significant prophylactic benefits, specifically in preventing Parkinson’s disease (odds ratio = 0.28, 95% confidence intervals = 0.09 to 0.93) compared to controls. Neither GLP-1 receptor agonists nor other SGLT2 inhibitors showed significant preventive effects for any of the investigated neurodegenerative conditions. Drop-out rates were comparable across all treatments.

**Conclusions:**

This comprehensive NMA reveals a novel and specific prophylactic effect of dapagliflozin against Parkinson’s disease, representing a potential breakthrough in preventive neurology. The specificity of dapagliflozin’s protective effect to Parkinson’s disease might rely on its highly selective inhibition to SGLT2. These findings provide important direction for future research and could inform preventive strategies for populations at risk of Parkinson’s disease.

**Trial registration:**

PROSPERO CRD42021252381.

**Supplementary Information:**

The online version contains supplementary material available at 10.1186/s12916-025-04018-w.

## Background

Glucagon-like peptide-1 (GLP-1) receptor agonists and sodium–glucose cotransporter 2 (SGLT2) inhibitors have emerged as novel glucose-lowering agents, featuring mechanisms of action distinct from those of conventional treatments [1]. GLP-1, an incretin hormone produced by intestinal L cells, enhances insulin release, slows gastric emptying, and suppresses glucagon secretion, which collectively contribute to reduced blood glucose levels [2]. Meanwhile, SGLT2 is produced in the proximal tubules of the kidneys, where SGLT2 inhibitor facilitates the lowering of blood glucose by limiting glucose reabsorption and encouraging its excretion through urine, thereby aiding in improved glycemic control for patients [3].

Beyond their primary role in managing blood sugar, additional therapeutic advantages of GLP-1 receptor agonists and SGLT2 inhibitors have been uncovered in recent years. Notably, SGLT2 inhibitors have shown cardiovascular [4] and renal protective effects in patients with diabetes [5]. Similarly, GLP-1 receptor agonists have been found to provide cardiovascular and renal benefits within the same population [6]. As a result of these expanded benefits, researchers are increasingly viewing GLP-1 receptor agonists and SGLT2 inhibitors as versatile, multi-functional drugs.

Recently, interest has been growing around the potential application of GLP-1 receptor agonists and SGLT2 inhibitors in managing neurodegenerative diseases [7]. Animal studies have demonstrated neuroprotective effects for these medications across various disease models [8, 9]. Clinicians are now focusing on the therapeutic potential of GLP-1 receptor agonists and SGLT2 inhibitors in addressing symptoms of Parkinson’s disease [10, 11] and Alzheimer’s disease [12]. Specifically, Mulvaney and colleagues observed that GLP-1 receptor agonists might improve motor symptoms in individuals with Parkinson’s disease [13]. This clinical observation could be supported by the basic evidence that GLP-1 receptor were not only expressed in gastrointestinal tract but also in several brain regions, such as hypothalamus, subfornical organ, nucleus of the solitary tract, and area postrema [14]. Some of these regions played an important role in some neurodegenerative diseases. For example, Zhou and the colleague demonstrated that the microstructural degeneration in hypothalamus may be associated with development of Parkinson’s disease [15]. In contrast, Vijiaratnam et al., by adding on exenatide to subjects with Parkinson’s disease, suggested that exenatide could not modify the Parkinson’s disease progression [16]. In a recent one traditional pair-wise meta-analysis by Albuquerque et al., the authors showed that overall GLP-1 receptor agonists relieve the motor symptoms in subjects with Parkinson’s disease [17]. Moreover, dapagliflozin, a particular SGLT2 inhibitor, has been shown to exhibit anti-inflammatory properties [18], which may hypothetically be significant in managing diverse neurodegenerative diseases. This proposed neuroprotective effect is supported by data from several large-scale trials on neurodegenerative conditions, including Parkinson’s disease and Alzheimer’s disease [19–21]. However, the precise mechanisms and physiological impacts of these medications remain largely unexplored.

Following these clinical trials, several meta-analyses have shown a beneficial effect of GLP-1 receptor agonists and SGLT2 inhibitors on symptoms of Parkinson’s disease [22] and Alzheimer’s disease [23]. As the adage goes, “Prevention is better than cure” [24]. From a public health perspective, prevention holds particular importance, as most neurodegenerative diseases are irreversible [25]. Although there have been traditional pairwise meta-analyses assessing the protective effects of these newer glucose-lowering drugs on neurodegenerative diseases [26–28], conclusive evidence remains elusive due to methodological limitations. Traditional pairwise meta-analyses, which group various medications together, yield an overall efficacy but lack the specificity needed for individual comparisons, leading to heterogeneity among the medications and diluting statistical significance. Network meta-analysis (NMA), which allows for direct comparisons among different medications, enhances the ability to make multiple treatment efficacy comparisons and assess the potential superiority of specific interventions at various dosages [29]. This approach offers a more detailed and evidence-based framework for guiding future clinical practices.

Given this context, a well-constructed NMA could provide comparative efficacy estimates and offer fresh perspectives on the relative benefits of these medications. To the best of our knowledge, no NMA has yet assessed the preventive potential of various GLP-1 receptor agonists and SGLT2 inhibitors in neurodegenerative diseases. Therefore, this NMA aims to (1) compare the preventive efficacy of GLP-1 receptor agonists and SGLT2 inhibitors across multiple neurodegenerative diseases; (2) identify the most effective agents for prevention; and (3) assess their relative safety profiles in preventive use.

## Methods

This network meta-analysis (NMA) followed the guidelines from the Preferred Reporting Items for Systematic Reviews and Meta-Analyses (PRISMA) with the extension for network meta-analyses (PRISMA NMA) [30] (Additional file: Tab. S1A-S1B). The study was registered in PROSPERO under registration number CRD42021252381 and received ethical approval from the Institutional Review Board at the Tri-Service General Hospital, National Defense Medical Center, Taipei, Taiwan (TSGHIRB No. B-109–29).

### Database searches and study identification

We performed comprehensive database searches in PubMed, Embase, ClinicalKey, Cochrane CENTRAL, ProQuest, ScienceDirect, Web of Science, and ClinicalTrials.gov (Additional file: Tab. S2) for studies published up to October 24, 2024. Two independent authors (PT Tseng and BY Zeng) conducted these searches, screened the titles and abstracts, and resolved any disagreements about study inclusion through consensus. Additionally, we manually reviewed reference lists from relevant review articles and meta-analyses to identify additional studies [7, 13, 22, 23, 26–28, 31–41]. No language restrictions were applied in the search.

### Inclusion and exclusion criteria

Since the main goal of the current NMA was to evaluate the prophylactic effect, the participants to be included should not have pre-existed neurodegenerative diseases at baseline. Therefore, the inclusion criteria for this NMA were based on the following PICOS model (Population, Intervention, Comparison, Outcome, and Study design): Population: Adults (≥ 18 years) without pre-existing neurodegenerative diseases; Intervention: prescription of GLP-1 receptor agonist or SGLT2 inhibitor at any dose; Comparison: Placebo, standard care, or active comparator; Outcomes: Incident cases of neurodegenerative diseases; Study design: Randomized controlled trials.

This NMA focused on assessing prophylactic effects; therefore, only participants without neurodegenerative diseases at baseline were included. To limit heterogeneity, we included only studies comparing GLP-1 receptor agonists or SGLT2 inhibitors. Eligible studies were limited to peer-reviewed randomized controlled trials (RCTs) and included (1) RCTs with participants free of neurodegenerative diseases at baseline; (2) RCTs involving GLP-1 receptor agonists or SGLT2 inhibitors; (3) studies on human participants; and (4) RCTs that systematically screened for adverse events or specifically targeted these outcomes.

Exclusion criteria included (1) studies that were not RCTs or peer-reviewed; (2) RCTs involving participants with pre-existing neurodegenerative conditions; (3) RCTs not directly comparing GLP-1 receptor agonists or SGLT2 inhibitors; (4) after checking full text, not report target outcome, either in primary/secondary outcome or in adverse event profile; and (5) animal studies. Because the currently available RCTs regarding such medications were designed to evaluate their treatment efficacy but not to detect incidence of neurodegenerative diseases, it would easily miss the occurrence of neurodegenerative diseases and result in potential reporting bias if they were not designed as systematically screening for adverse events. Only RCTs with systematic screening for adverse events or those directly assessing our target outcomes were included to enhance reliability and reduce selective reporting bias [42].

### Methodological quality appraisal

Two independent authors evaluated the risk of bias for each study using the Cochrane Risk of Bias Tool 1.0 [43], achieving an inter-rater reliability of 0.85. Any differences were resolved by consulting a third author.

### Outcome definition

Due to the variability in the methods used to record targeted events, we defined our primary outcome as the “event numbers in registry systems.” Specifically, we counted total event occurrences rather than the number of affected patients. The primary outcome was the total number of overall neurodegenerative disease events recorded in registry systems. Based on the book by Suescun [44] and review article by Koenig [45], the neurodegenerative diseases were defined to include (1) Parkinson’s disease, (2) Alzheimer’s disease, (3) Lewy body dementia, (4) multiple sclerosis, (5) amyotrophic lateral sclerosis, (6) Frontotemporal dementia, and (7) Huntington’s disease. The safety profile was assessed through drop-out rates (i.e., participants who withdrew from the study before completion for any reason).

### Data extraction, management, and conversion

Data extraction was independently performed by two authors (PT Tseng and BY Zeng), recording demographic data, study design, treatment details, primary outcomes, and safety information. If essential data were missing, we reached out to corresponding authors. The data extraction adhered to protocols from the Cochrane Handbook for Systematic Reviews of Interventions and other pertinent medical literature [46].

Dose definitions followed original RCT classifications [19–21, 47–65]: canagliflozin (low: 100 mg, and high: 300 mg); ertugliflozin (low: 5 mg, and high: 15 mg); injectable semaglutide (low: 0.5 mg, and high: 1.0 mg); empagliflozin (low: 1–10 mg, and high: 25–50 mg).

### Statistical analyses

For analysis with multiple treatment arms, a random-effects model was used in the NMA [66], employing MetaInsight (version 4.0.2, Complex Reviews Support Unit, National Institute for Health Research, London, UK) within a frequentist framework. MetaInsight is a web-based platform for conducting NMAs via the netmeta package in R software, designed for frequentist statistical analysis [67].

For categorical data, a continuity correction of single-zero-event studies was applied in the meta-analytical procedure. However, for studies with zero event in both the intervention and the control arms, such a correction was not applied to avoid increasing the bias. Rather, we would exclude that comparison instead [68, 69]. Forest plots were created to present odds ratios (ORs) with 95% confidence intervals (95%CIs) for effect size calculation [70]. We then generated treatment rankings and effect sizes for direct and indirect comparisons, tabulated accordingly. A two-tailed *p*-value less than 0.05 indicated statistical significance.

### Inconsistency evaluation

The “node-splitting” method was applied to evaluate the potential inconsistency between direct and indirect evidence, a method particularly beneficial in NMA when trial-level data are available. To be specific, the inconsistency test of node-splitting method in MetaInsight was conducted based on R package netmeta (Gerta Rücker, Guido Schwarzer, Ulrike Krahn and Jochem König 2017) in the platform of R software-based webpage [67, 71].

### Sensitivity analyses

To assess the robustness of our findings, we conducted a subgroup analysis by grouping RCTs by seven primary outcome categories (e.g., Parkinson’s disease, Alzheimer’s disease, Lewy body dementia, multiple sclerosis, amyotrophic lateral sclerosis, frontotemporal dementia, and Huntington’s disease).

Further, to re-affirm the reliability and the convergence of the investigated treatment estimates, we arrange sensitivity analysis with Bayesian-based NMA to re-run the analytic process of the main primary outcome. Further, we arranged Bayesian-based surface under the cumulative ranking (SUCRA) evaluations by Litmus Rank-O-Gram and radial SUCRA plots [72] to evaluate the rank of superiority of individual regimen. Finally, we used a deviance model to evaluate the fit and influence of treatment effect estimates by comparing deviance from the NMA and the unrelated mean effects inconsistency model, examining residual deviance across study arms, and analyzing leverage versus residual deviance [73].

### General declaration

This study complies with the principles outlined in the Declaration of Helsinki.

## Results

### Eligibility of the studies

**Fig. 1:**
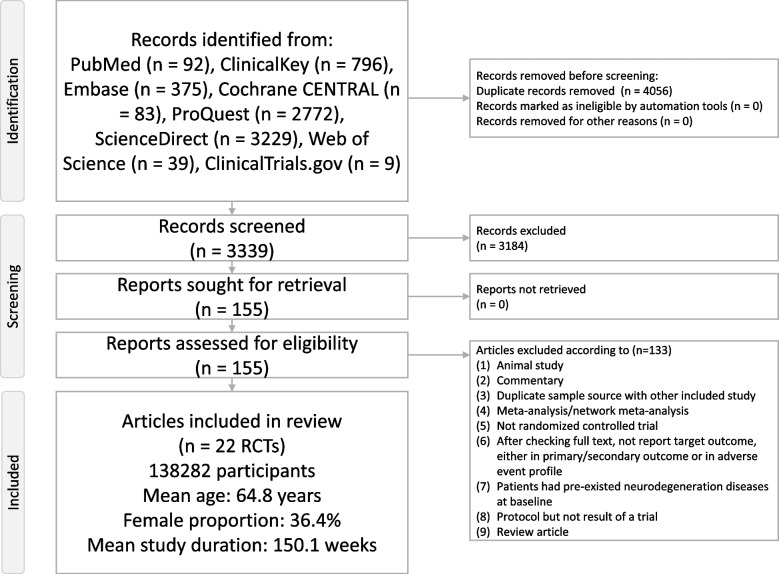
PRISMA2020 Flowchart of current network meta-analysis

Figure 1 illustrates the flowchart summarizing the literature search and screening process for this NMA. After excluding 133 articles for various reasons (Additional file: Tab. S3) [7–13, 16, 22, 23, 26–28, 31–41, 50, 51, 53, 54, 61, 63, 74–176], a total of 22 RCTs were included in the analysis [19–21, 47–65]. The selected studies involved 138,282 participants (mean age = 64.8 years, range 57.1 to 71.9 years; mean female proportion = 36.4%, range 23.4 to 46.5%) (Additional file: Tab. S4). The average study duration was 150.1 weeks (range 24 to 281 weeks). In total, 17 experimental arms were examined, comprising 1 placebo/control arm and 16 various dosage ranges of different GLP-1 receptor agonists/SGLT2 inhibitors arms. The investigated GLP-1 receptor agonists included liraglutide, albiglutide, dulaglutide, exenatide, semaglutide, and lixisenatide. The investigated SGLT2 inhibitors included canagliflozin, empagliflozin, ertugliflozin, dapagliflozin, and sotagliflozin.

### Primary outcome: overall events of neurodegenerative diseases

**Fig. 2:**
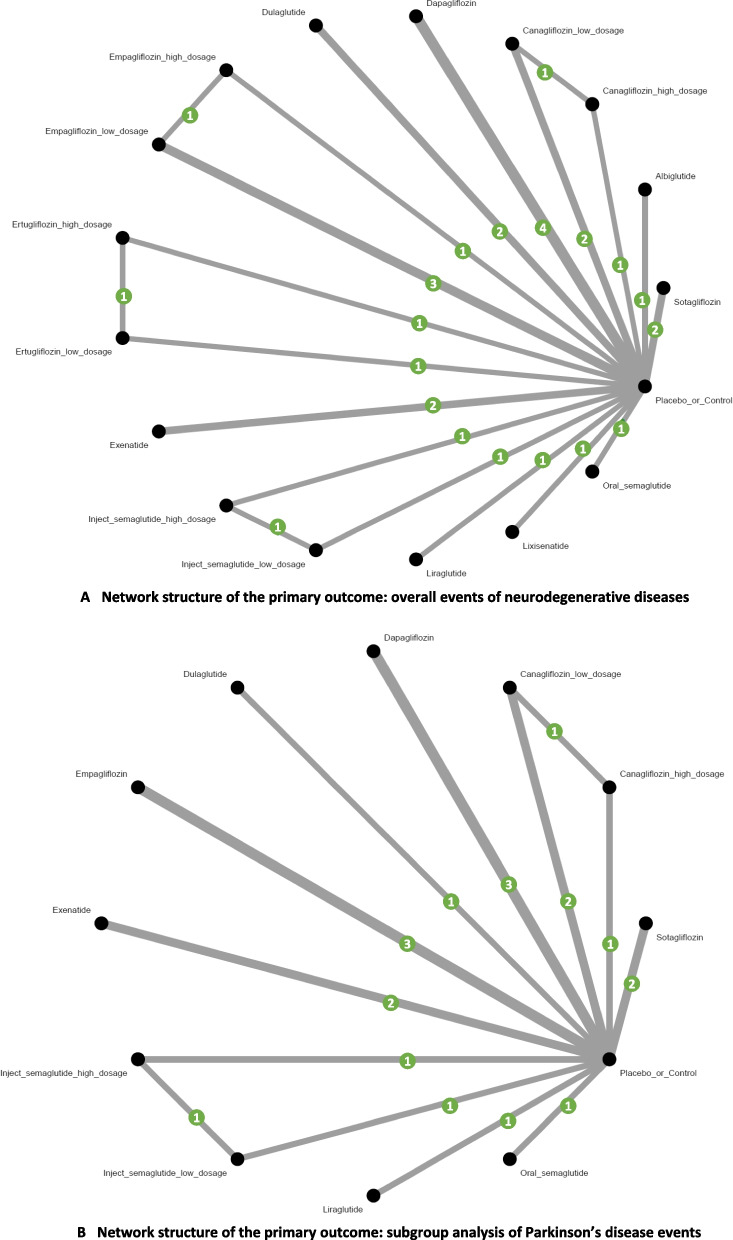
**A** Network structure of the primary outcome: overall events of neurodegenerative diseases. **A** depicts the structure of the overall network meta-analysis of primary outcome. The lines between nodes represent direct comparisons from various trials, with the numbers over the lines indicating the number of trials providing these comparisons for each specific treatment. The thickness of the lines corresponds to the number of trials linked to the network. **B** Network structure of the primary outcome: subgroup analysis of Parkinson’s disease events. **B** depicts the structure of the subgroup analysis focusing on Parkinson’s disease. The lines between nodes represent direct comparisons from various trials, with the numbers over the lines indicating the number of trials providing these comparisons for each specific treatment. The thickness of the lines corresponds to the number of trials linked to the network

**Fig. 3:**
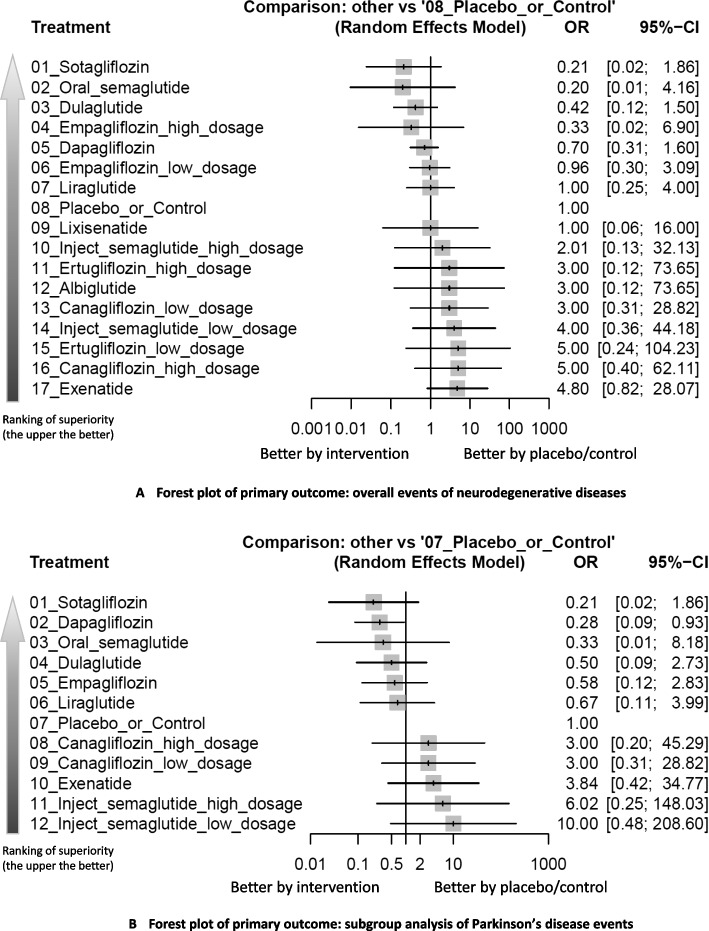
**A** Forest plot of primary outcome: overall events of neurodegenerative diseases. When the effect size (expressed as odds ratio) is less than 1, the specified treatment is associated with fewer neurodegenerative disease events compared to placebo/controls. **B** Forest plot of primary outcome: subgroup analysis of Parkinson’s disease events. When the effect size (expressed as odds ratio) is less than 1, the specified treatment is associated with fewer Parkinson’s disease events compared to placebo/controls. Dosage definition: canagliflozin (low: 100 mg, and high: 300 mg); ertugliflozin (low: 5 mg, and high: 15 mg); injectable semaglutide (low: 0.5 mg, and high: 1.0 mg); empagliflozin (low: 1–10 mg, and high: 25–50 mg). Abbreviations: 95%CIs: 95% confidence intervals; GLP-1 agonist: glucagon-like peptide-1 agonist; NMA: network meta-analysis; OR: odds ratio; RCT: randomized controlled trial; SGLT2 inhibitor: sodium–glucose cotransporter 2 inhibitor

Analysis of overall neurodegenerative disease events revealed no statistically significant differences between any investigated treatments and the control group. However, several interventions showed promising trends: Sotagliflozin demonstrated a trend of favorable profile, OR = 0.21 (95%CIs = 0.02 to 1.86), which had a consistent trend across subgroups although not achieved statistical significance. Oral semaglutide ranked second (OR = 0.20, 95%CIs = 0.01 to 4.16), demonstrating similar magnitude of effect to sotagliflozin. Dulaglutide ranked third (OR = 0.42, 95%CIs = 0.12 to 1.50), showing consistent effects across sensitivity analyses (Figs. 2A, 3A, and Table 1).

**Table 1:** League table of the primary outcome: overall events of neurodegenerative diseases

Sotagliflozin							0.21 [0.02; 1.86]									
Sotagliflozin							0.21 [0.02; 1.86]									
1.06 [0.03; 44.45]	Oral_ semaglutide						0.20 [0.01; 4.16]									
0.50 [0.04; 6.25]	0.47 [0.02; 12.78]	Dulaglutide					0.42 [0.12; 1.50]									
0.65 [0.02; 27.48]	0.61 [0.01; 45.29]	1.29 [0.05; 35.13]	Empagliflozin_ high_dosage		0.33 [0.01; 8.19]		0.33 [0.01; 8.15]									
0.30 [0.03; 3.10]	0.29 [0.01; 6.65]	0.60 [0.13; 2.74]	0.47 [0.02; 11.01]	Dapagliflozin			0.70 [0.31; 1.60]									
0.22 [0.02; 2.59]	0.21 [0.01; 5.37]	0.44 [0.08; 2.45]	0.34 [0.02; 7.16]	0.73 [0.17; 3.03]	Empagliflozin_ low_dosage		0.96 [0.30; 3.09]									
0.21 [0.02; 2.79]	0.20 [0.01; 5.62]	0.42 [0.06; 2.76]	0.33 [0.01; 9.31]	0.70 [0.14; 3.51]	0.96 [0.16; 5.88]	Liraglutide	1.00 [0.25; 4.00]									
0.21 [0.02; 1.86]	0.20 [0.01; 4.16]	0.42 [0.12; 1.50]	0.33 [0.02; 6.90]	0.70 [0.31; 1.60]	0.96 [0.30; 3.09]	1.00 [0.25; 4.00]	Placebo_ or_Control	1.00 [0.06; 15.99]	0.50 [0.03; 7.97]	0.33 [0.01; 8.19]	0.33 [0.01; 8.19]	0.33 [0.03; 3.21]	0.25 [0.02; 2.76]	0.20 [0.01; 4.17]	0.20 [0.01; 4.17]	0.21 [0.04; 1.22]
0.21 [0.01; 7.18]	0.20 [0.00; 12.20]	0.42 [0.02; 8.89]	0.33 [0.01; 20.13]	0.70 [0.04; 12.62]	0.96 [0.05; 19.47]	1.00 [0.05; 22.20]	1.00 [0.06; 15.99]	Lixisenatide								
0.11 [0.00; 3.58]	0.10 [0.00; 6.08]	0.21 [0.01; 4.43]	0.16 [0.00; 10.04]	0.35 [0.02; 6.29]	0.48 [0.02; 9.71]	0.50 [0.02; 11.07]	0.50 [0.03; 7.97]	0.50 [0.01; 25.15]	Inject_ semaglutide_ high_dosage				0.50 [0.05; 5.55]			
0.07 [0.00; 3.39]	0.07 [0.00; 5.49]	0.14 [0.00; 4.40]	0.11 [0.00; 9.06]	0.23 [0.01; 6.36]	0.32 [0.01; 9.68]	0.33 [0.01; 10.92]	0.33 [0.01; 8.19]	0.33 [0.00; 23.03]	0.67 [0.01; 46.23]	Ertugliflozin_ high_dosage				0.60 [0.08; 4.54]		
0.07 [0.00; 3.39]	0.07 [0.00; 5.49]	0.14 [0.00; 4.40]	0.11 [0.00; 9.06]	0.23 [0.01; 6.36]	0.32 [0.01; 9.68]	0.33 [0.01; 10.92]	0.33 [0.01; 8.19]	0.33 [0.00; 23.02]	0.67 [0.01; 46.22]	1.00 [0.01; 92.45]	Albiglutide					
0.07 [0.00; 1.63]	0.07 [0.00; 2.94]	0.14 [0.01; 1.89]	0.11 [0.00; 4.86]	0.23 [0.02; 2.60]	0.32 [0.03; 4.10]	0.33 [0.02; 4.75]	0.33 [0.03; 3.21]	0.33 [0.01; 11.96]	0.67 [0.02; 24.02]	1.00 [0.02; 50.47]	1.00 [0.02; 50.47]	Canagliflozin_ low_dosage			0.60 [0.08; 4.54]	
0.05 [0.00; 1.35]	0.05 [0.00; 2.40]	0.11 [0.01; 1.59]	0.08 [0.00; 3.96]	0.17 [0.01; 2.22]	0.24 [0.02; 3.47]	0.25 [0.02; 4.01]	0.25 [0.02; 2.76]	0.25 [0.01; 9.80]	0.50 [0.05; 5.55]	0.75 [0.01; 41.01]	0.75 [0.01; 41.02]	0.75 [0.03; 20.32]	Inject_ semaglutide_ low_dosage			
0.04 [0.00; 1.77]	0.04 [0.00; 2.93]	0.08 [0.00; 2.26]	0.07 [0.00; 4.83]	0.14 [0.01; 3.25]	0.19 [0.01; 4.98]	0.20 [0.01; 5.64]	0.20 [0.01; 4.17]	0.20 [0.00; 12.21]	0.40 [0.01; 24.52]	0.60 [0.08; 4.54]	0.60 [0.01; 49.44]	0.60 [0.01; 26.45]	0.80 [0.02; 38.41]	Ertugliflozin_ low_dosage		
0.04 [0.00; 1.18]	0.04 [0.00; 2.06]	0.08 [0.01; 1.41]	0.07 [0.00; 3.41]	0.14 [0.01; 1.98]	0.19 [0.01; 3.09]	0.20 [0.01; 3.54]	0.20 [0.02; 2.48]	0.20 [0.00; 8.46]	0.40 [0.01; 16.99]	0.60 [0.01; 35.20]	0.60 [0.01; 35.20]	0.60 [0.09; 4.19]	0.80 [0.02; 25.95]	1.00 [0.02; 51.66]	Canagliflozin_ high_dosage	

*0.04 [0.00; 0.73]

0.04 [0.00; 1.40]

*0.09 [0.01; 0.77]

0.07 [0.00; 2.31]

0.15 [0.02; 1.03]

0.20 [0.02; 1.67]

0.21 [0.02; 1.97]

0.21 [0.04; 1.22]

0.21 [0.01; 5.58]

0.42 [0.02; 11.21]

0.63 [0.02; 24.21]

0.63 [0.02; 24.20]

0.62 [0.04; 11.03]

0.83 [0.04; 16.45]

1.04 [0.03; 35.00]

1.04 [0.05; 22.62]

Exenatide

Data present as OR [95%CIs]. Pairwise (upper-right portion) and network (lower-left portion) meta-analysis results are presented as estimate effect sizes for the outcome of overall events of neurodegenerative diseases. Interventions are reported in order of mean ranking of beneficially prophylactic effect on overall events of neurodegenerative diseases, and outcomes are expressed as odds ratio (OR) (95% confidence intervals) (95%CIs). For the pairwise meta-analyses, OR of less than 1 indicate that the treatment specified in the row got more beneficial effect than that specified in the column. For the network meta-analysis (NMA), OR of less than 1 indicate that the treatment specified in the column got more beneficial effect than that specified in the row. Bold results marked with * indicate statistical significance

Network meta-analysis results for all treatment comparisons are presented in Fig. 3A and detailed in Table 1. While these results suggest potential protective effects, the wide confidence intervals and lack of statistical significance highlight the need for larger, targeted studies.

### Subgroup analyses of seven categories of neurodegenerative diseases

In our analysis of specific neurodegenerative conditions, dapagliflozin emerged as the only intervention showing significant prophylactic benefits against Parkinson’s disease (OR = 0.28, 95%CIs = 0.09 to 0.93) compared to control. While sotagliflozin demonstrated the most favorable point estimate (OR = 0.21, 95%CIs = 0.02 to 1.86) and ranked first in the network, the wide confidence intervals precluded statistical significance. Dapagliflozin ranked second in the network hierarchy for Parkinson’s disease prevention (Figs. 2B, 3B, and Table 2).

**Table 2:** League table of the primary outcome: subgroup analysis of Parkinson’s disease events

Sotagliflozin						0.21 [0.02; 1.86]					
0.75 [0.06; 8.93]	Dapagliflozin					*0.28 [0.09; 0.93]					
0.64 [0.01; 30.49]	0.85 [0.03; 25.84]	Oral_semaglutide				0.33 [0.01; 8.18]					
0.42 [0.03; 6.68]	0.57 [0.07; 4.49]	0.67 [0.02; 24.95]	Dulaglutide			0.50 [0.09; 2.73]					
0.36 [0.02; 5.33]	0.48 [0.07; 3.50]	0.57 [0.02; 20.24]	0.86 [0.08; 8.70]	Empagliflozin		0.58 [0.12; 2.83]					
0.32 [0.02; 5.31	0.42 [0.05; 3.64]	0.50 [0.01; 19.55]	0.75 [0.06; 8.84]	0.88 [0.08; 9.52]	Liraglutide	0.67 [0.11; 3.99]					
0.21 [0.02; 1.86]	*0.28 [0.09; 0.93]	0.33 [0.01; 8.18]	0.50 [0.09; 2.73]	0.58 [0.12; 2.83]	0.67 [0.11; 3.99]	Placebo_or_Control	0.33 [0.01; 8.19]	0.33 [0.03; 3.21]	0.26 [0.03; 2.36]	0.17 [0.01; 4.08]	0.10 [0.00; 2.08]
0.07 [0.00; 2.29]	0.09 [0.00; 1.83]	0.11 [0.00; 7.38]	0.17 [0.01; 4.10]	0.19 [0.01; 4.50]	0.22 [0.01; 5.74]	0.33 [0.02; 5.03]	Canagliflozin_ high_dosage	1.00 [0.10; 9.64]			
0.07 [0.00; 1.63]	0.09 [0.01; 1.22]	0.11 [0.00; 5.61]	0.17 [0.01; 2.83]	0.19 [0.01; 3.08]	0.22 [0.01; 3.99]	0.33 [0.03; 3.21]	1.00 [0.11; 8.97]	Canagliflozin_ low_dosage			
0.06 [0.00; 1.22]	*0.07 [0.01; 0.90]	0.09 [0.00; 4.23]	0.13 [0.01; 2.11]	0.15 [0.01; 2.29]	0.17 [0.01; 2.98]	0.26 [0.03; 2.36]	0.78 [0.02; 25.82]	0.78 [0.03; 18.41]	Exenatide		
0.04 [0.00; 1.69]	0.05 [0.00; 1.43]	0.06 [0.00; 5.12]	0.08 [0.00; 3.11]	0.10 [0.00; 3.44]	0.11 [0.00; 4.34]	0.17 [0.01; 4.08]	0.50 [0.01; 33.13]	0.50 [0.01; 25.10]	0.64 [0.01; 31.06]	Inject_semaglutide_ high_dosage	0.60 [0.08; 4.57]
*0.02 [0.00; 0.89]	*0.03 [0.00; 0.74]	0.03 [0.00; 2.75]	0.05 [0.00; 1.62]	0.06 [0.00; 1.79]	0.07 [0.00; 2.27]	0.10 [0.00; 2.08]	0.30 [0.01; 17.63]	0.30 [0.01; 13.23]	0.38 [0.01; 16.36]	0.60 [0.08; 4.57]	Inject_semaglutide_ low_dosage

Data present as OR [95%CIs]. Pairwise (upper-right portion) and network (lower-left portion) meta-analysis results are presented as estimate effect sizes for the outcome of events of Parkinson’s disease. Interventions are reported in order of mean ranking of beneficially prophylactic effect on events of Parkinson’s disease, and outcomes are expressed as odds ratio (OR) (95% confidence intervals) (95%CIs). For the pairwise meta-analyses, OR of less than 1 indicate that the treatment specified in the row got more beneficial effect than that specified in the column. For the network meta-analysis (NMA), OR of less than 1 indicate that the treatment specified in the column got more beneficial effect than that specified in the row. Bold results marked with * indicate statistical significance

Dosage definition: Canagliflozin (Low: 100mg, and High: 300mg); Ertugliflozin (Low: 5mg, and High: 15mg); Injectable semaglutide (Low: 0.5mg, and High: 1.0mg); Empagliflozin (Low: 1-10mg, and High: 25-50mg)

*Abbreviation*: *95%CIs *95% confidence intervals, *GLP-1* agonist: glucagon-like peptide-1 agonist, *NMA* network meta-analysis, *OR *odds ratio, *RCT* randomized controlled trial, *SGLT2 inhibitor* sodium–glucose cotransporter 2 inhibitor

For Alzheimer’s disease, our network meta-analysis revealed no significant preventive effects across all investigated interventions (Additional file: Fig. S1A, Additional file: Fig. S2A, and Additional file: Tab. S5A). This pattern of non-significant findings extended to several other neurodegenerative conditions. Specifically, we found no significant prophylactic benefits for Lewy body dementia (Additional file: Fig. S1B, Additional file: Fig. S2B, and Additional file: Tab. S5B), multiple sclerosis (Additional file: Fig. S1C, Additional file: Fig. S2C, and Additional file: Tab. S5C), or amyotrophic lateral sclerosis (Additional file: Fig. S1D, Additional file: Fig. S2D, and Additional file: Tab. S5D).

The evidence base for frontotemporal dementia was limited to a single RCT, and no eligible trials reported outcomes for Huntington’s disease, preventing meaningful network meta-analysis for these conditions. This paucity of data highlights an important gap in current research regarding the preventive potential of these medications for less common neurodegenerative disorders.

### Safety profile: drop-out rate

Only the canagliflozin was associated with significantly less drop-out rates than the control group did (high dosage canagliflozin: OR = 0.57, 95%CIs = 0.40 to 0.83; low dosage canagliflozin OR = 0.65, 95%CIs = 0.47 to 0.91). Among these interventions, high dosage canagliflozin ranked the best (Additional file: Fig. S1E, Additional file: Fig. S2E, and Additional file: Tab. S5E).

### Sensitivity analysis with Bayesian-based NMA

The relative ranking of interventions remained stable across different analytical approaches (Additional file: Fig. S3, and Additional file: Fig. S4). Generally, the main results of primary outcome did not differ between frequentist-based NMA and Bayesian-based NMA (Additional file: Fig. S5). The Bayesian-based SUCRA ranking list had been depicted in Additional file: Tab. S6 and Additional file: Fig. S6A-S6B. The deviation-model assessment did not demonstrate significant deviation among the current NMA (Additional file: Fig. S7A-S7C).

### Risk of bias and inconsistency

We identified that 82.5% (127/154 items), 14.3% (22/154 items), and 3.2% (5/154 items) of the included studies had low, unclear, and high risks of bias, respectively (Additional file: Fig. S8). The inconsistency test, evaluating the assumption of consistency, showed no significant inconsistencies in the present NMA (Additional file: Tab. S7A-S7G).

## Discussion

This comprehensive network meta-analysis revealed a novel and specific prophylactic benefit of dapagliflozin against Parkinson’s disease, marking a potentially important advancement in preventive neurology. While multiple GLP-1 receptor agonists and SGLT2 inhibitors were evaluated across various neurodegenerative conditions, only dapagliflozin demonstrated significant preventive effects (OR = 0.28, 95%CIs = 0.09 to 0.93). This specificity is particularly noteworthy, as no significant prophylactic benefits were observed for other major neurodegenerative conditions, including Alzheimer’s disease, Lewy body dementia, multiple sclerosis, and amyotrophic lateral sclerosis. The evidence base for less common conditions, specifically frontotemporal dementia and Huntington’s disease, proved insufficient for definitive conclusions, highlighting critical gaps in current research. These findings suggest that the neuroprotective mechanisms of these medications may be more selective than previously hypothesized, with particular relevance to Parkinson’s disease pathophysiology.

This study represents the first network meta-analysis to systematically evaluate the prophylactic potential of GLP-1 receptor agonists and SGLT2 inhibitors across neurodegenerative conditions. While previous research has primarily investigated the therapeutic effects of these medications in established neurodegenerative diseases [13, 22, 23], our analysis specifically addresses their preventive capabilities. This distinction is crucial, as the irreversible nature of neurodegenerative processes makes prevention potentially more impactful than treatment from a public health perspective [24]. Our network meta-analytic approach offers several advantages over traditional pair-wise meta-analyses, enabling direct comparisons between individual medications and doses, thus providing more nuanced evidence of their relative prophylactic efficacy [29]. This methodological strength allows us to identify specific agents, such as dapagliflozin, that may offer particular promise for preventive interventions, while also highlighting areas where current evidence remains insufficient.

One key finding of this NMA was that only the dapagliflozin, a highly selective and reversible SGLT2 inhibitor, was associated with significantly less events of Parkinson’s disease than the control group did. As addressed in the method section, the current NMA did not include participants with pre-existed neurodegenerative diseases, including Parkinson’s disease. Further, among the included RCTs, none of them specifically recruit subjects with pre-existed Parkinson’s disease. Therefore, the findings of our NMA might suggest a potential of protective benefit of dapagliflozin to patients who had indications for such medications but without current Parkinson’s disease. Dapagliflozin has been found to exert potential neuroprotective effects against neurodegenerative dysfunctions via ROS-dependent AKT/GSK-3β/NF-κB and DJ-1/Nrf2 pathways in the rotenone-induced Parkinson’s disease rat model [177]. Further, the prescription of dapagliflozin could help in the attenuation of motor dysfunction in Parkinson’s disease animal model [177]. In addition, dapagliflozin could also reduce the histopathologic alterations and α-synuclein expression and increase the tyrosine hydroxylase and dopamine levels [177], which physiopathology had been found to be one of the etiology of Parkinson’s disease [178, 179]. Another potential mechanism which could involve dapagliflozin’s neuroprotective properties relied on its anti-inflammatory property. Previous studies have suggested that Parkinson’s disease might be associated with interleukin-1 related over-oxidative environment [180]. Elevated cytokines, such as interleukin-1 beta (IL-1B) [180], in the brain can alter neural function and lead to neural death. Dapagliflozin has been shown to reduce systemic inflammation, including plasma IL-1B levels, in patients treated with dapagliflozin for 12 months [181]. Finally, different from the other SGLT2 inhibitors, the dapagliflozin exhibited its properties of highly selection to SGLT2 and reversibility [182]. In contrary, the other SGLT2 inhibitors, which also inhibit the SGLT1, might interfere with the neuroprotective effects of SGLT1 in the central nervous system [183]. This pharmacodynamical theory could be supported by the insignificant findings of those SGLT2 inhibitors with SGLT1 affinity. Finally, although no formal reports have directly linked this reduction in inflammation to the prevention of Parkinson’s disease, it may serve as a basis for hypothesizing that dapagliflozin could help prevent this condition. In addition to the above mechanism, the better comparative efficacy on glycated hemoglobin (HbA1c) by dapagliflozin use than other SGLT2 inhibitors might also be another explanation of the preferably protective effects by dapagliflozin on Parkinson’s disease [184]. Since the existence of diabetes mellitus would increase risk of Parkinson’s disease to an extent of 23–85% [185], dapagliflozin would serve as one of the choices of anti-diabetic medications who treating diabetic subjects with risk of Parkinson’s disease.

Regarding other neurodegenerative diseases, such as Alzheimer’s disease, dementia of Lewy body, multiple sclerosis, amyotrophic lateral sclerosis, frontotemporal dementia, and Huntington’s disease, this NMA did not find any significant benefits from the investigated medications. This may be due to the fact that the most neurodegenerative diseases often involve more chronic changes [186], and the duration of the RCTs included in the analysis may not have been long enough to detect meaningful differences.

### Strengths and limitations

This network meta-analysis offers several methodological strengths that enhance the reliability and clinical utility of our findings. The NMA design enables direct comparisons between different GLP-1 receptor agonists and SGLT2 inhibitors, providing more comprehensive evidence than traditional pairwise meta-analyses. Our rigorous methodology included exclusive focus on peer-reviewed randomized controlled trials, ensuring high-quality evidence while minimizing potential bias. By specifically excluding participants with pre-existing neurodegenerative conditions, we were able to isolate true prophylactic effects. Furthermore, our detailed subgroup analyses across individual neurodegenerative conditions offer clinicians granular evidence to inform preventive strategies for specific patient populations. Finally, to enhance the reliability, we also arranged sensitivity analysis with Bayesian-based NMA to re-affirm the main result of the current study, which sensitivity analysis revealed similar results.

Despite these strengths, several important limitations warrant consideration. The primary limitation relates to study duration; although the included trials averaged 150.1 weeks of follow-up, this timeframe may be insufficient to fully capture the development of neurodegenerative conditions, which typically evolve over decades [181]. Our stringent focus on RCTs, while ensuring methodological rigor, potentially excluded valuable observational data from long-term cohort studies. Additionally, the variation in diagnostic approaches across multi-country trials presents a notable limitation. The lack of standardized neuropsychiatric assessment and structured diagnostic interviews may have introduced heterogeneity in case identification, potentially affecting the precision of our effect estimates. Besides, since this is a statistical study, we could not know the actual molecular and physiological mechanism between the neuroprotection and dapagliflozin prescription. Finally, since the original data did not provide further information regarding classified outcomes according to achieving glycemic control (i.e. HbA1c < 7 or HbA1c > 7) or gender-related difference, we could not do further sensitivity analysis based on these issues. These limitations suggest the need for longer-term, standardized studies specifically designed to assess preventive effects in neurodegenerative conditions based on levels of glycemic control or gender-specific design.

## Conclusions

This comprehensive network meta-analysis reveals a potentially important breakthrough in neurodegenerative disease prevention, demonstrating that dapagliflozin, an SGLT2 inhibitor, significantly reduces the risk of Parkinson’s disease development (OR = 0.28, 95% CIs = 0.09 to 0.93). This finding is particularly noteworthy given the absence of significant prophylactic effects for other investigated agents across multiple neurodegenerative conditions, including Alzheimer’s disease, Lewy body dementia, multiple sclerosis, and amyotrophic lateral sclerosis.

The specificity of dapagliflozin’s preventive effect suggests distinct neuroprotective mechanisms that warrant further investigation. These findings have important implications for clinical practice and future research directions, particularly in Parkinson’s disease prevention. Future studies should focus on elucidating the underlying mechanisms of dapagliflozin’s neuroprotective effects, determining optimal preventive strategies, and identifying patient populations most likely to benefit from prophylactic intervention.

While longer-term studies are needed to fully understand the preventive potential of these medications, our findings provide valuable evidence to guide both clinical decision-making and the design of future preventive trials in neurodegenerative diseases. The results particularly highlight the need for targeted investigation of dapagliflozin’s role in Parkinson’s disease prevention, potentially opening a new avenue in preventive neurology.

## Supplementary Information

Supplementary Material 1: Fig. S1. (A) Network structure of primary outcome: subgroup analysis of Alzheimer’s disease events; (B) Network structure of primary outcome: subgroup analysis of dementia of Lewy body events; (C) Network structure of primary outcome: subgroup analysis of multiple sclerosis events; (D) Network structure of primary outcome: subgroup analysis of amyotrophic lateral sclerosis events; (E) Network structure of safety profile: drop-out rate. Fig. S2. (A) Forest plot of primary outcome: subgroup analysis of Alzheimer’s disease events; (B) Forest plot of primary outcome: subgroup analysis of dementia of Lewy body events; (C) Forest plot of primary outcome: subgroup analysis of multiple sclerosis events; (D) Forest plot of primary outcome: subgroup analysis of amyotrophic lateral sclerosis events; (E) Forest plot of safety profile: drop-out rate. Fig. S3. Summary plot of ranking of primary outcome (overall events of neurodegenerative diseases). Fig. S4. Individual study result of primary outcome: overall events of neurodegenerative diseases. Fig. S5. Bayesian-based forest plot of primary outcome: overall events of neurodegenerative diseases. Fig. S6. (A) Bayesian-based Litmus Rank-O-Gram rank plot of primary outcome: overall events of neurodegenerative diseases; (B) Bayesian-based radial surface under the cumulative ranking of primary outcome: overall events of neurodegenerative diseases. Fig. S7. (A) Bayesian-based residual deviance NMA/UME model of primary outcome: overall events of neurodegenerative diseases; (B) Bayesian-based per-arm residual deviance of primary outcome: overall events of neurodegenerative diseases; (C) Bayesian-based leverage plot of primary outcome: overall events of neurodegenerative diseases. Fig. S8. Detailed risk of bias in each study. Tab. S1. (A) PRISMA 2020 checklist of the current network meta-analysis; (B) PRISMA 2020 abstract checklist of the current network meta-analysis. Tab. S2. Keyword used in each database and search results. Tab. S3. Excluded studies and reason. Tab. S4. Characteristics of the included studies. Tab. S5. (A) League table of primary outcome: subgroup of Alzheimer’s disease events; (B) League table of primary outcome: subgroup of dementia of Lewy body events; (C) League table of primary outcome: subgroup of multiple sclerosis events; (D) League table of primary outcome: subgroup of amyotrophic lateral sclerosis events; (E) League table of safety profile: drop-out rate. Tab. S6. Surface under the cumulative ranking of primary outcome: overall events of neurodegenerative diseases. Tab. S7. (A) Inconsistency within the primary outcome: overall events of neurodegenerative diseases; (B) Inconsistency within the primary outcome: subgroup of Parkinson’s disease events; (C) Inconsistency within the primary outcome: subgroup of Alzheimer’s disease events; (D) Inconsistency within the primary outcome: subgroup of dementia of Lewy body events; (E) Inconsistency within the primary outcome: subgroup of multiple sclerosis events; (F) Inconsistency within the primary outcome: subgroup of amyotrophic lateral sclerosis events; (G) Inconsistency within the safety profile: drop-out rate. Legends to Combined Additional files [7–13, 16, 19–23, 26–28, 31–41, 47–65, 74–176, 187].

## Data Availability

All the data of the current study were available upon reasonable request to the corresponding authors.

## Abbreviations

95%CI: 95% confidence interval
GLP-1: Glucagon-like peptide-1
HbA1c: Glycated hemoglobin
NMA: Network meta-analysis
OR: Odds ratio
PICOS: Population, intervention, comparison, outcome, and study design
PRISMA: Preferred Reporting Items for Systematic Reviews and Meta-Analyses
RCT: Randomized controlled trial
SGLT2: Sodium–glucose cotransporter 2
SUCRA: Surface under the cumulative ranking
