# Supplementary material for: The pharmacodynamics-based prophylactic benefits of GLP-1 receptor agonists and SGLT2 inhibitors on neurodegenerative diseases: evidence from a network meta-analysis
Source: BMC Med. 2025 Apr 7;23:197. doi: 10.1186/s12916-025-04018-w (PMC11974209; doi:10.1186/s12916-025-04018-w)
Supplement: Supplementary file 1 — Supplementary Material 1: Fig. S1. (A) Network structure of primary outcome: subgroup analysis of Alzheimer’s disease events; (B) Network structure of primary outcome: subgroup analysis of dementia of Lewy body events; (C) Network structure of primary outcome: subgroup analysis of multiple sclerosis events; (D) Network structure of primary outcome: subgroup analysis of amyotrophic lateral sclerosis events; (E) Network structure of safety profile: drop-out rate. Fig. S2. (A) Forest plot of primary outcome: subgroup analysis of Alzheimer’s disease events; (B) Forest plot of primary outcome: subgroup analysis of dementia of Lewy body events; (C) Forest plot of primary outcome: subgroup analysis of multiple sclerosis events; (D) Forest plot of primary outcome: subgroup analysis of amyotrophic lateral sclerosis events; (E) Forest plot of safety profile: drop-out rate. Fig. S3. Summary plot of ranking of primary outcome (overall events of neurodegenerative diseases). Fig. S4. Individual study result of primary outcome: overall events of neurodegenerative diseases. Fig. S5. Bayesian-based forest plot of primary outcome: overall events of neurodegenerative diseases. Fig. S6. (A) Bayesian-based Litmus Rank-O-Gram rank plot of primary outcome: overall events of neurodegenerative diseases; (B) Bayesian-based radial surface under the cumulative ranking of primary outcome: overall events of neurodegenerative diseases. Fig. S7. (A) Bayesian-based residual deviance NMA/UME model of primary outcome: overall events of neurodegenerative diseases; (B) Bayesian-based per-arm residual deviance of primary outcome: overall events of neurodegenerative diseases; (C) Bayesian-based leverage plot of primary outcome: overall events of neurodegenerative diseases. Fig. S8. Detailed risk of bias in each study. Tab. S1. (A) PRISMA 2020 checklist of the current network meta-analysis; (B) PRISMA 2020 abstract checklist of the current network meta-analysis. Tab. S2. Keyword used in each database and [file 12916_2025_4018_MOESM1_ESM.docx]

**List of content in Additional File**

**The Pharmacodynamics-Based Prophylactic Benefits of GLP-1 Receptor Agonists and SGLT2 Inhibitors on Neurodegenerative Diseases:**

**Evidence from a Network Meta-Analysis**

*Ping-Tao Tseng, et al.*

| Fig. S1 | (A) Network structure of primary outcome: subgroup analysis of Alzheimer’s disease events |
| --- | --- |
|  | (B) Network structure of primary outcome: subgroup analysis of dementia of Lewy body events |
|  | (C) Network structure of primary outcome: subgroup analysis of multiple sclerosis events |
|  | (D) Network structure of primary outcome: subgroup analysis of amyotrophic lateral sclerosis events |
|  | (E) Network structure of safety profile: drop-out rate |
| Fig. S2 | (A) Forest plot of primary outcome: subgroup analysis of Alzheimer’s disease events |
|  | (B) Forest plot of primary outcome: subgroup analysis of dementia of Lewy body events |
|  | (C) Forest plot of primary outcome: subgroup analysis of multiple sclerosis events |
|  | (D) Forest plot of primary outcome: subgroup analysis of amyotrophic lateral sclerosis events |
|  | (E) Forest plot of safety profile: drop-out rate |
| Fig. S3 | Summary plot of ranking of primary outcome (overall events of neurodegenerative diseases) |
| Fig. S4 | Individual study result of primary outcome: overall events of neurodegenerative diseases |
| Fig. S5 | Bayesian-based forest plot of primary outcome: overall events of neurodegenerative diseases |
| Fig. S6 | (A) Bayesian-based Litmus Rank-O-Gram rank plot of primary outcome: overall events of neurodegenerative diseases |
|  | (B) Bayesian-based radial surface under the cumulative ranking of primary outcome: overall events of neurodegenerative diseases |
| Fig. S7 | (A) Bayesian-based residual deviance NMA/UME model of primary outcome: overall events of neurodegenerative diseases |
|  | (B) Bayesian-based per-arm residual deviance of primary outcome: overall events of neurodegenerative diseases |
|  | (C) Bayesian-based leverage plot of primary outcome: overall events of neurodegenerative diseases |
| Fig. S8 | Detailed risk of bias in each study |
| Tab. S1 | (A) PRISMA 2020 checklist of the current network meta-analysis |
|  | (B) PRISMA 2020 abstract checklist of the current network meta-analysis |
| Tab. S2 | Keyword used in each database and search results |
| Tab. S3 | Excluded studies and reason |
| Tab. S4 | Characteristics of the included studies |
| Tab. S5 | (A) League table of primary outcome: subgroup of Alzheimer’s disease events |
|  | (B) League table of primary outcome: subgroup of dementia of Lewy body events |
|  | (C) League table of primary outcome: subgroup of multiple sclerosis events |
|  | (D) League table of primary outcome: subgroup of amyotrophic lateral sclerosis events |
|  | (E) League table of safety profile: drop-out rate |
| Tab. S6 | Surface under the cumulative ranking of primary outcome: overall events of neurodegenerative diseases |
| Tab. S7 | (A) Inconsistency within the primary outcome: overall events of neurodegenerative diseases |
|  | (B) Inconsistency within the primary outcome: subgroup of Parkinson’s disease events |
|  | (C) Inconsistency within the primary outcome: subgroup of Alzheimer’s disease events |
|  | (D) Inconsistency within the primary outcome: subgroup of dementia of Lewy body events |
|  | (E) Inconsistency within the primary outcome: subgroup of multiple sclerosis events |
|  | (F) Inconsistency within the primary outcome: subgroup of amyotrophic lateral sclerosis events |
|  | (G) Inconsistency within the safety profile: drop-out rate |

**Fig. S1A network structure of primary outcome: subgroup analysis of Alzheimer’s disease events**

**
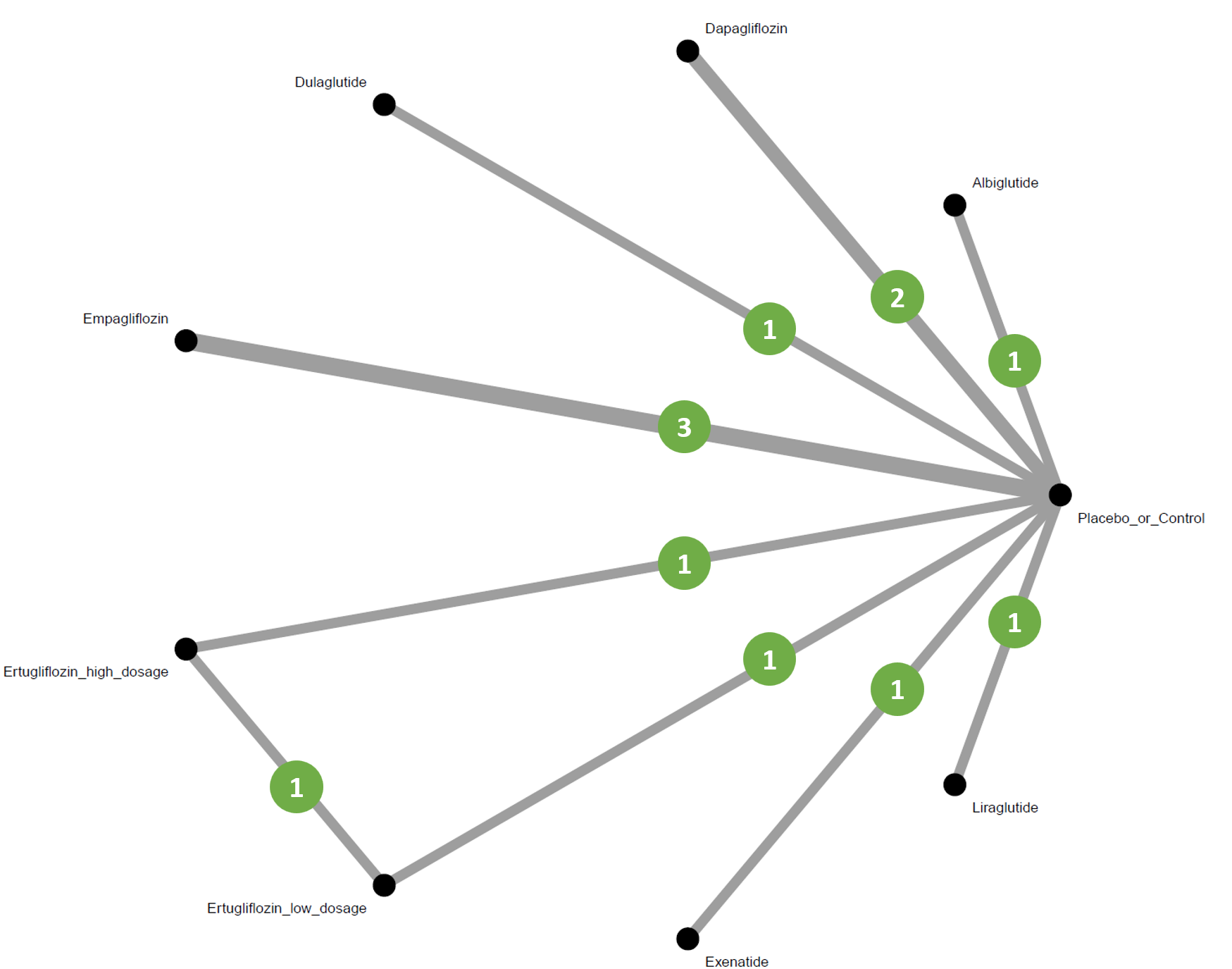
**

**Fig. S1B network structure of primary outcome: subgroup analysis of dementia of Lewy body events**

**
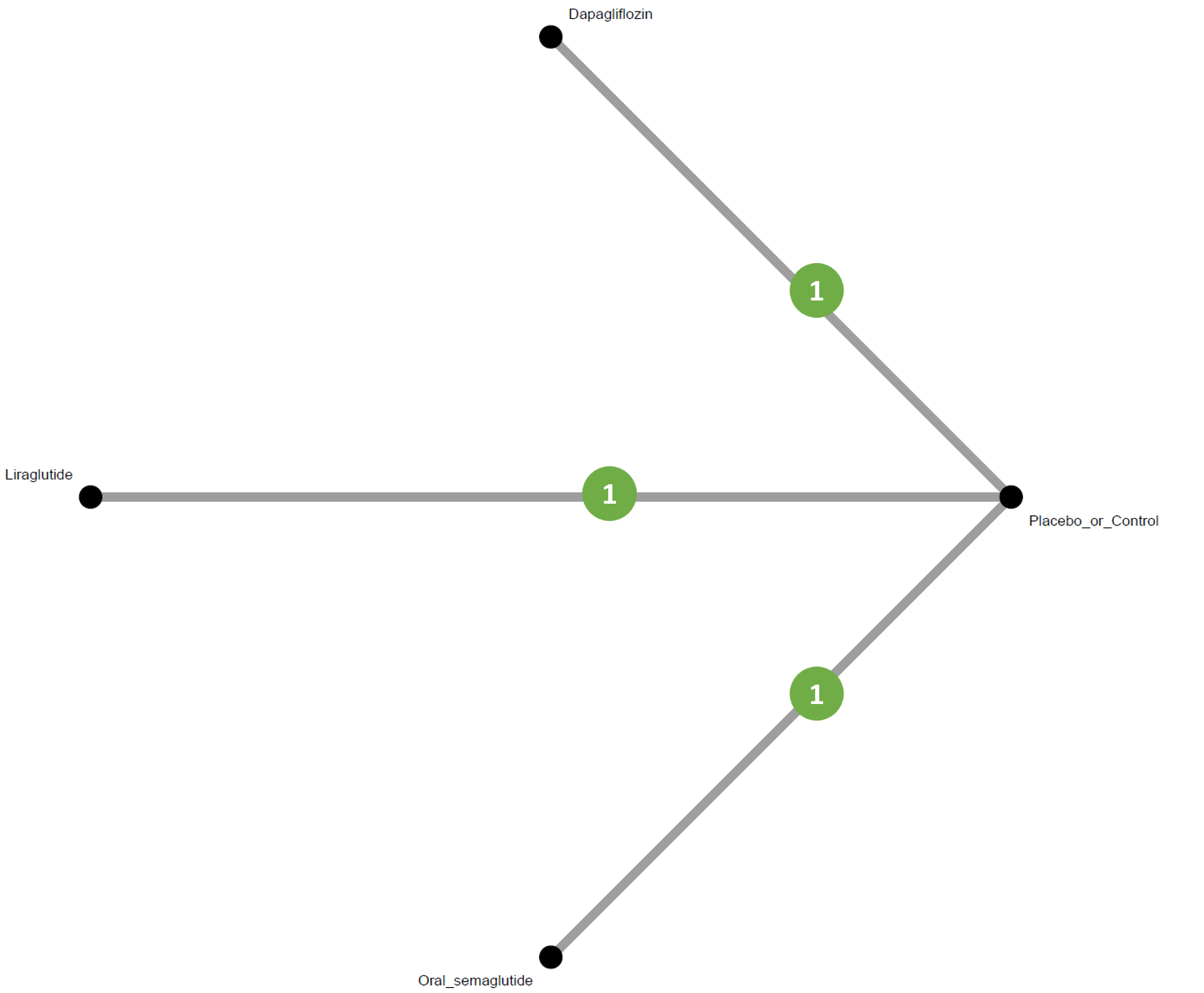
**

**Fig. S1C network structure of primary outcome: subgroup analysis of multiple sclerosis events**

**
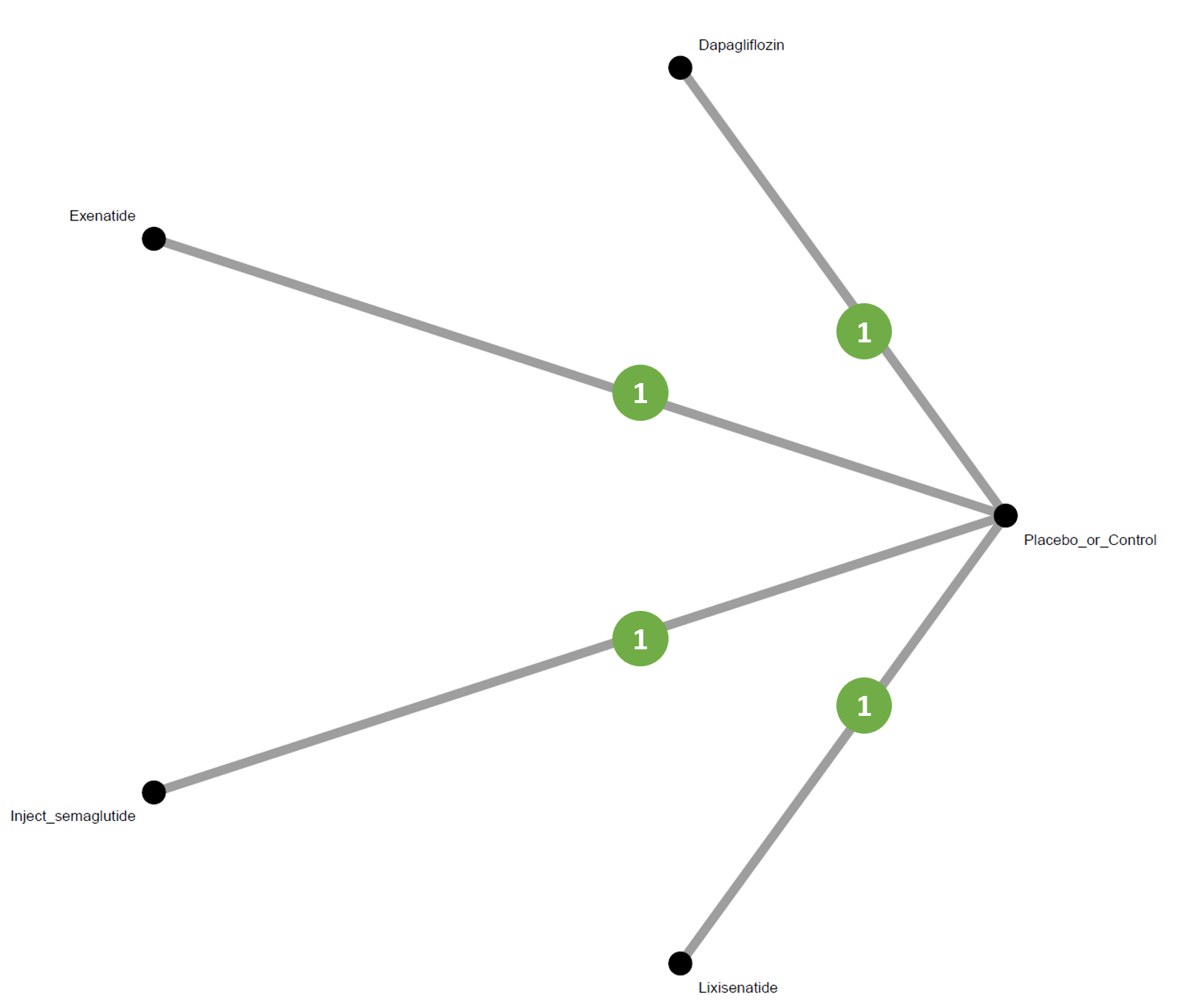
**

**Fig. S1D network structure of primary outcome: subgroup analysis of amyotrophic lateral sclerosis events**

**
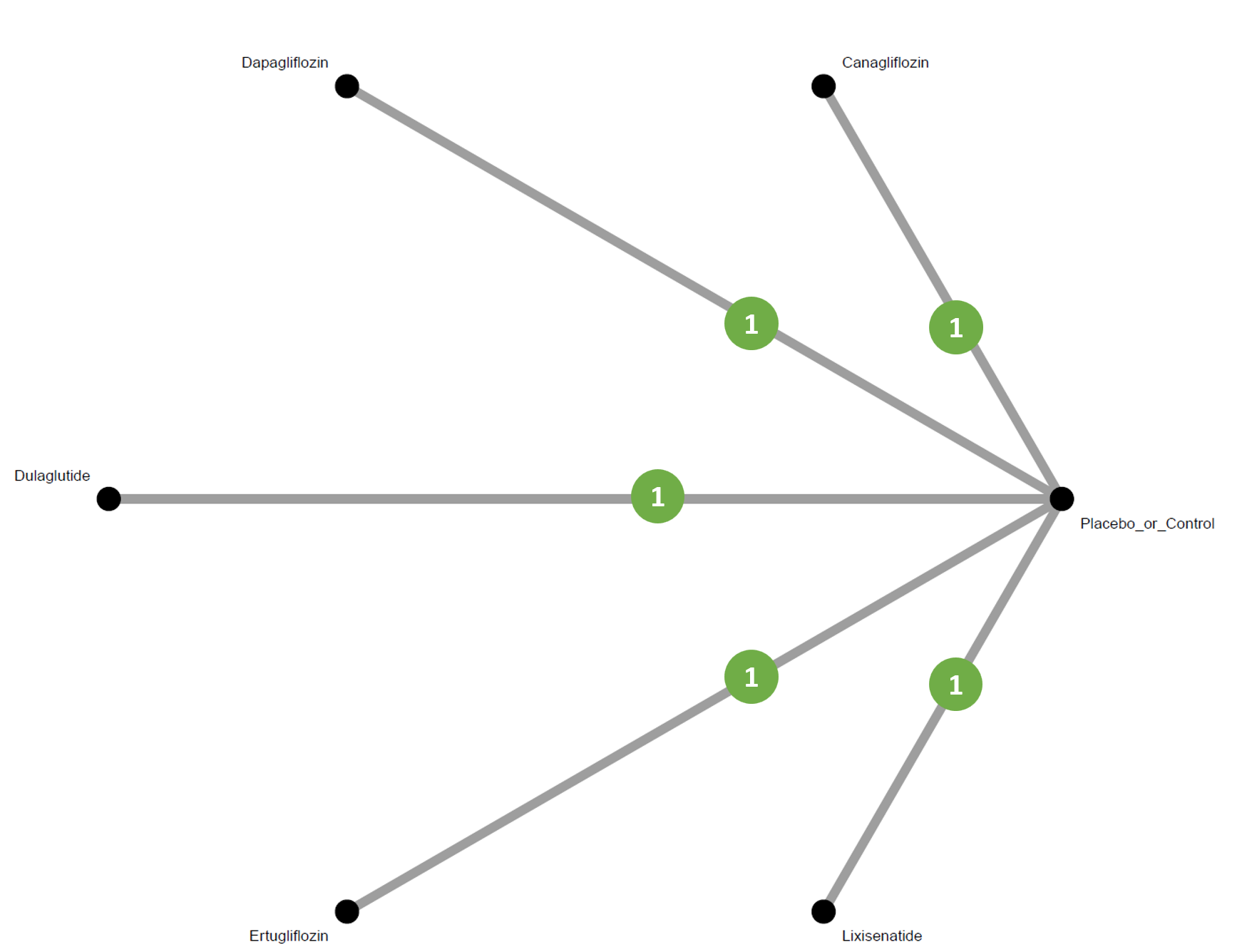
**

**Fig. S1E network structure of safety profile: drop-out rate**

**
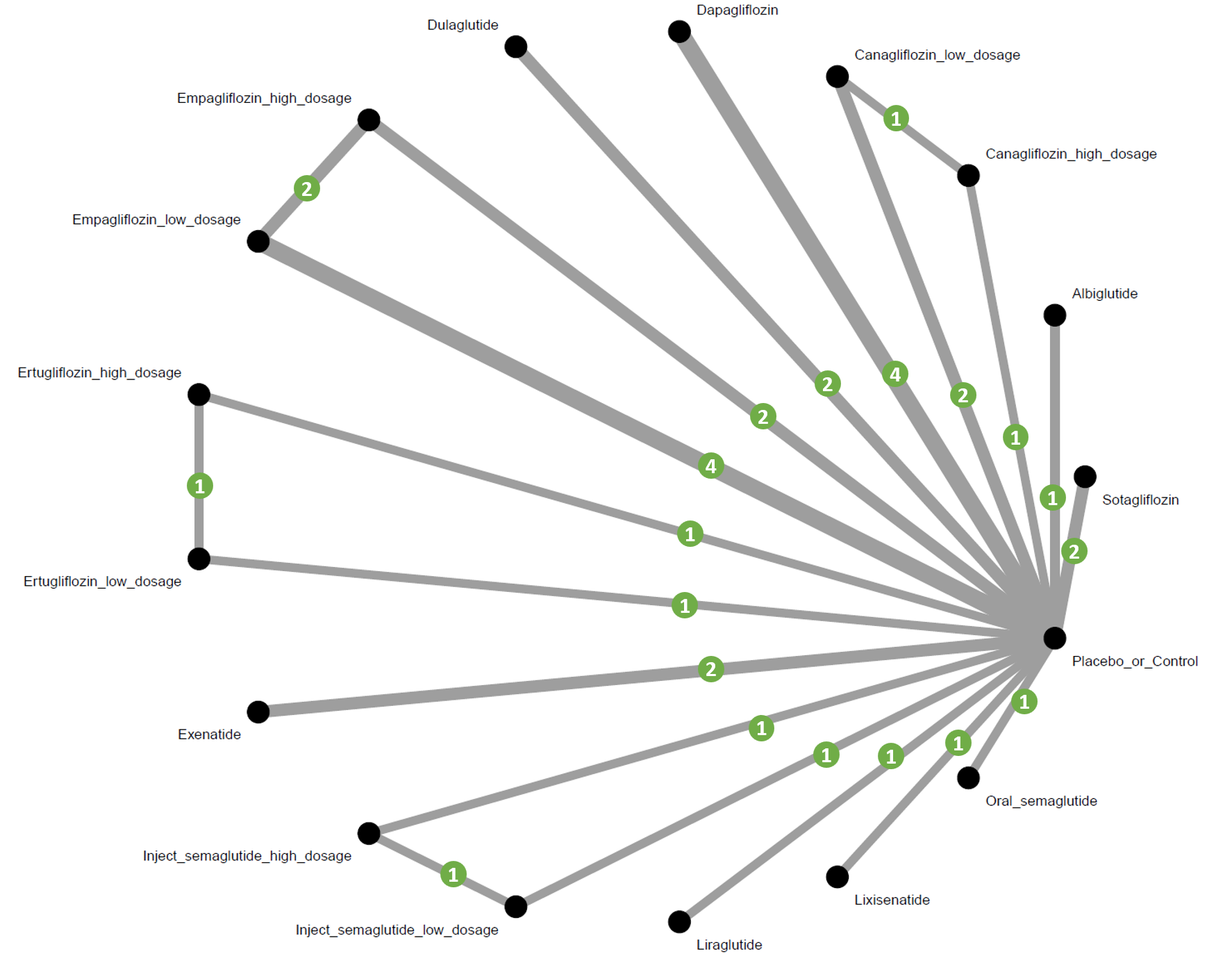
**

**Figure legend of Fig. S1A-S1E**

The structure of the network meta-analysis. The lines between nodes represent direct comparisons from various trials, with the numbers over the lines indicating the number of trials providing these comparisons for each specific treatment. The thickness of the lines corresponds to the number of trials linked to the network.

***Abbreviation for Fig. S1A-S1E:***

*95%CIs: 95% confidence intervals; GLP-1 agonist: glucagon-like peptide-1 agonist; NMA: network meta-analysis; OR: odds ratio; RCT: randomized controlled trial; SGLT2 inhibitor: sodium–glucose cotransporter 2 inhibitor*

*Dosage definition: Canagliflozin (Low: 100mg, and High: 300mg); Ertugliflozin (Low: 5mg, and High: 15mg); Injectable semaglutide (Low: 0.5mg, and High: 1.0mg); Empagliflozin (Low: 1-10mg, and High: 25-50mg).*

**Fig. S2A forest plot of primary outcome: subgroup analysis of Alzheimer’s disease events**

**
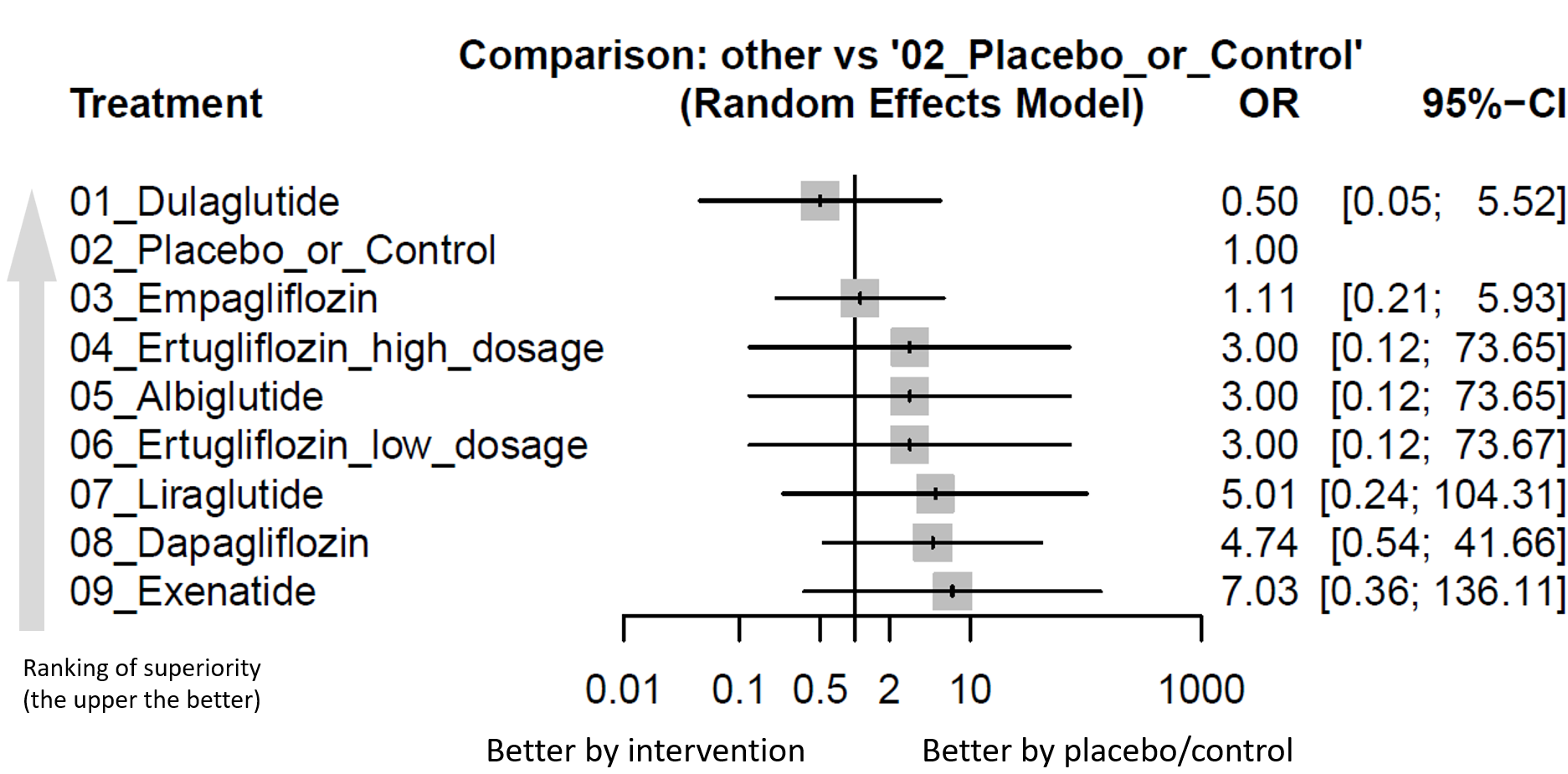
**

**Fig. S2B forest plot of primary outcome: subgroup analysis of dementia of Lewy body events**

**
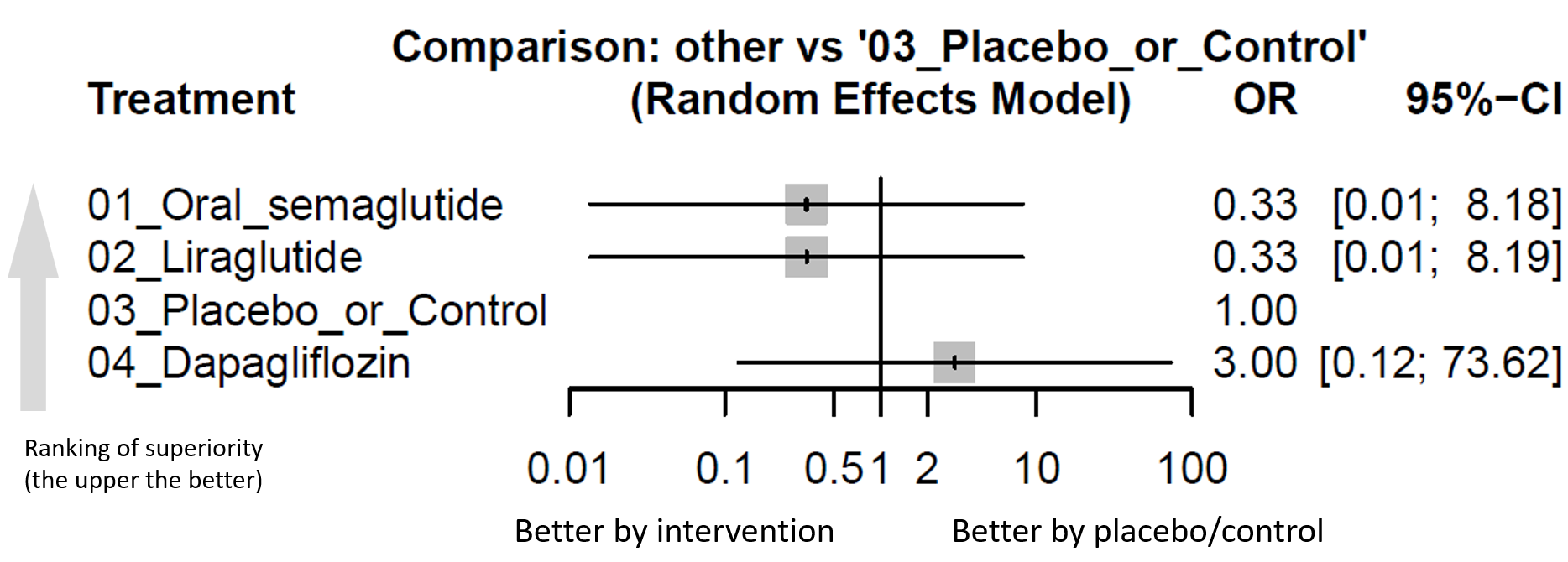
**

**Fig. S2C forest plot of primary outcome: subgroup analysis of multiple sclerosis events**

**
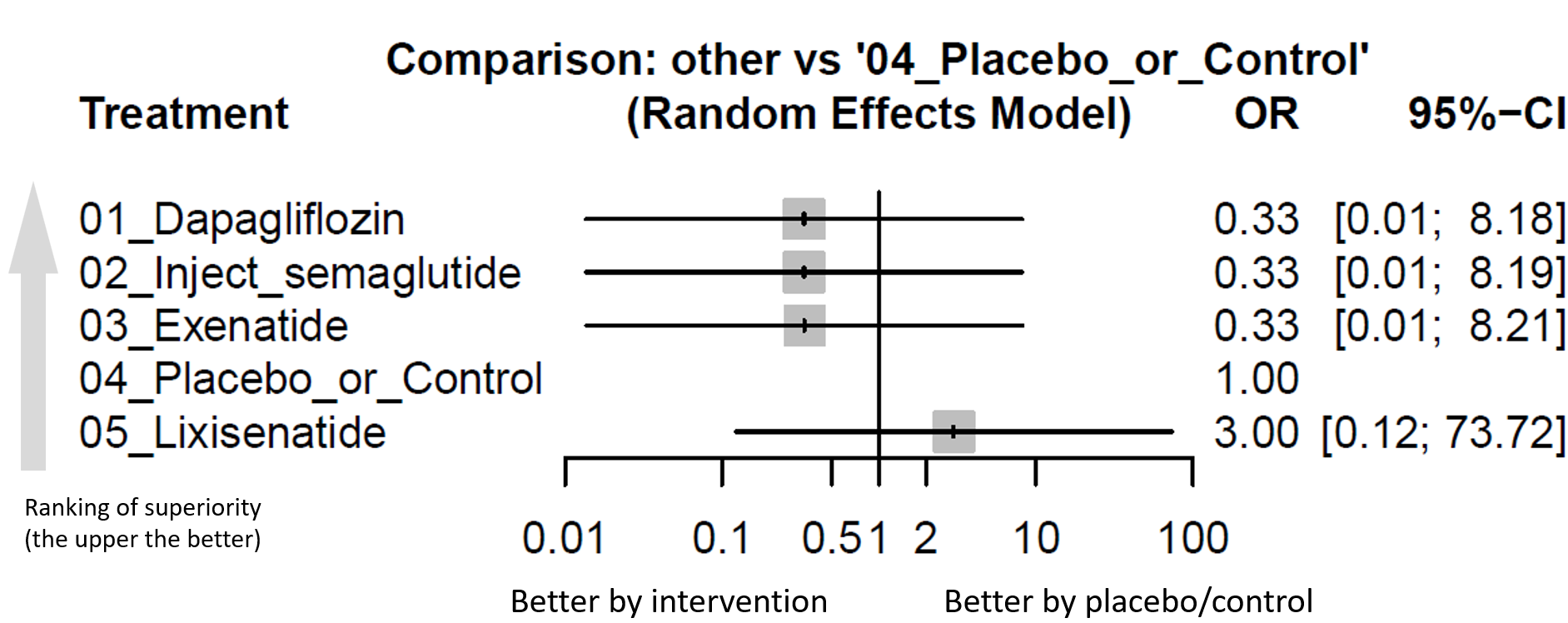
**

**Fig. S2D forest plot of primary outcome: subgroup analysis of amyotrophic lateral sclerosis events**

**
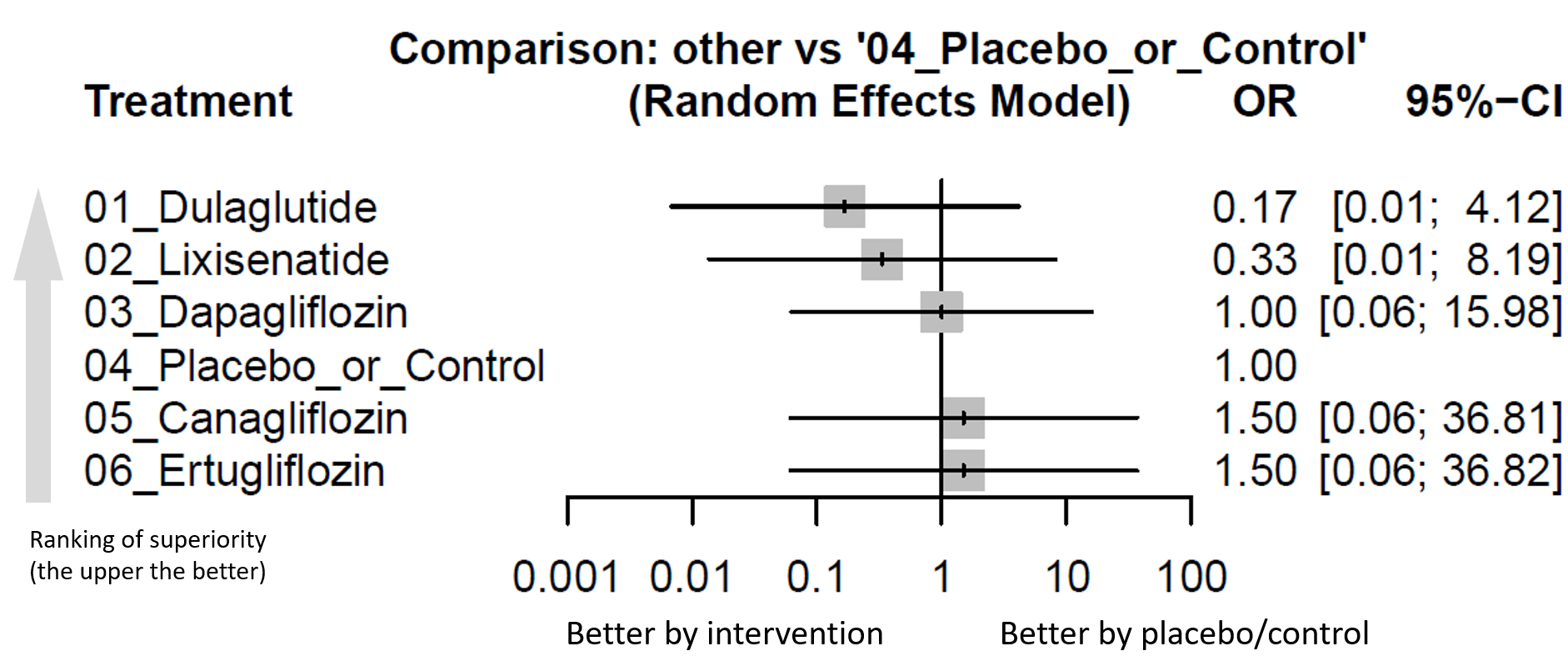
**

**Fig. S2E forest plot of safety profile: drop-out rate**

**
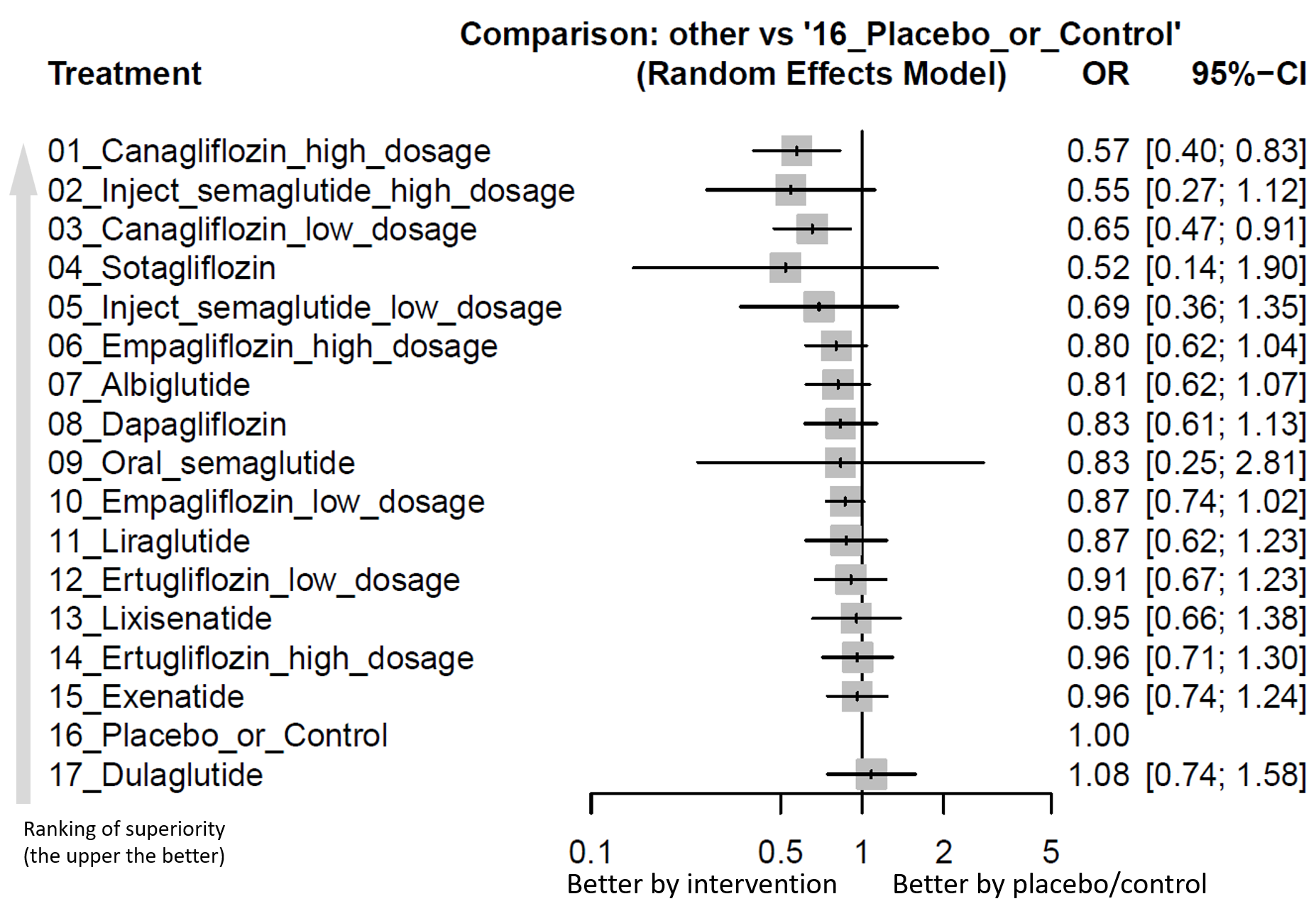
**

***Abbreviation for Fig. S2A-S2E:***

*95%CIs: 95% confidence intervals; GLP-1 agonist: glucagon-like peptide-1 agonist; NMA: network meta-analysis; OR: odds ratio; RCT: randomized controlled trial; SGLT2 inhibitor: sodium–glucose cotransporter 2 inhibitor*

*Dosage definition: Canagliflozin (Low: 100mg, and High: 300mg); Ertugliflozin (Low: 5mg, and High: 15mg); Injectable semaglutide (Low: 0.5mg, and High: 1.0mg); Empagliflozin (Low: 1-10mg, and High: 25-50mg).*

**Fig. S3 summary plot of ranking of primary outcome (overall events of neurodegenerative diseases)**

**
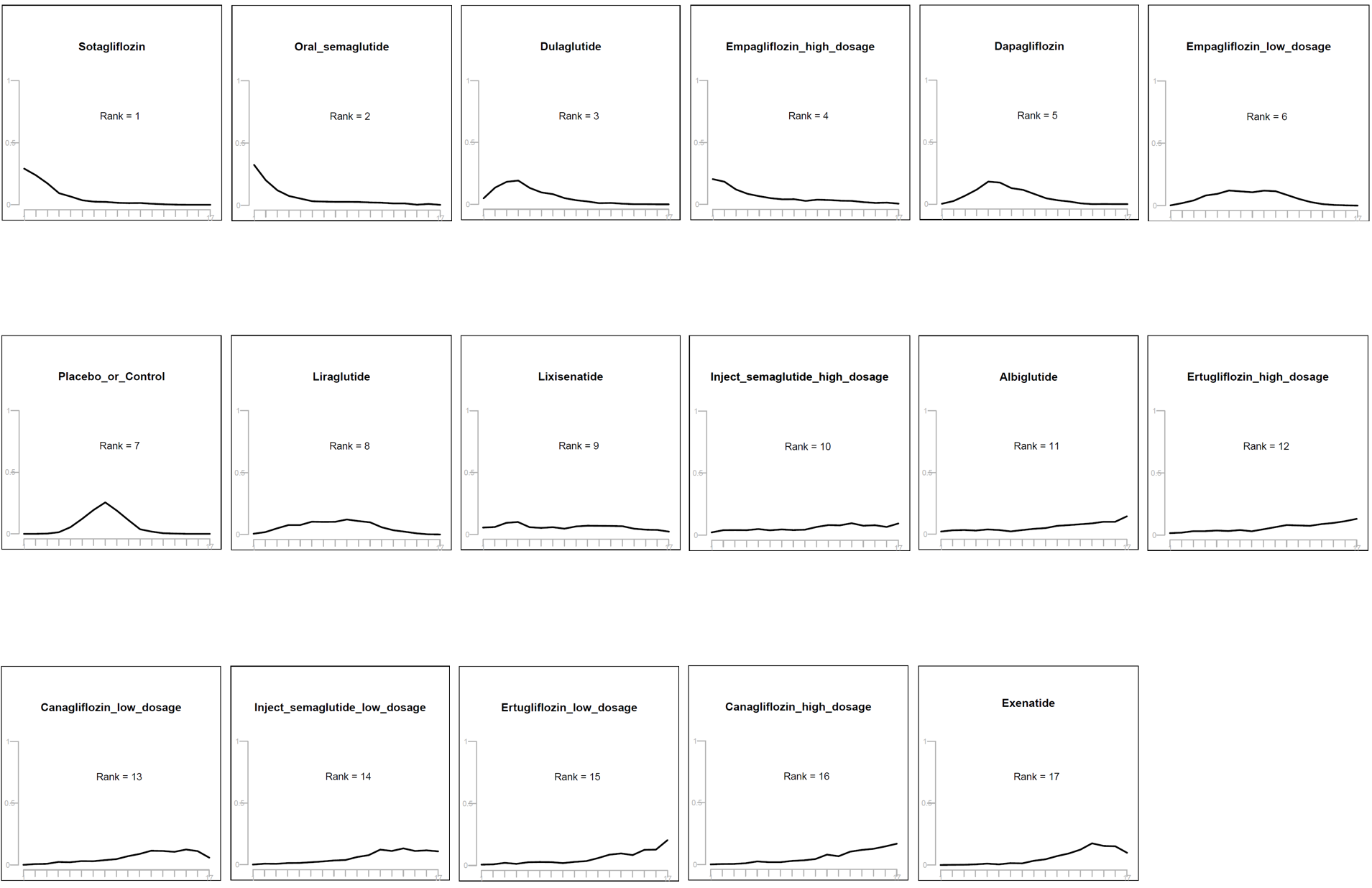
**

***Abbreviation for Fig. S3:***

*95%CIs: 95% confidence intervals; GLP-1 agonist: glucagon-like peptide-1 agonist; NMA: network meta-analysis; OR: odds ratio; RCT: randomized controlled trial; SGLT2 inhibitor: sodium–glucose cotransporter 2 inhibitor*

*Dosage definition: Canagliflozin (Low: 100mg, and High: 300mg); Ertugliflozin (Low: 5mg, and High: 15mg); Injectable semaglutide (Low: 0.5mg, and High: 1.0mg); Empagliflozin (Low: 1-10mg, and High: 25-50mg).*

**Fig. S4 Individual study result of primary outcome: overall events of neurodegenerative diseases**

**
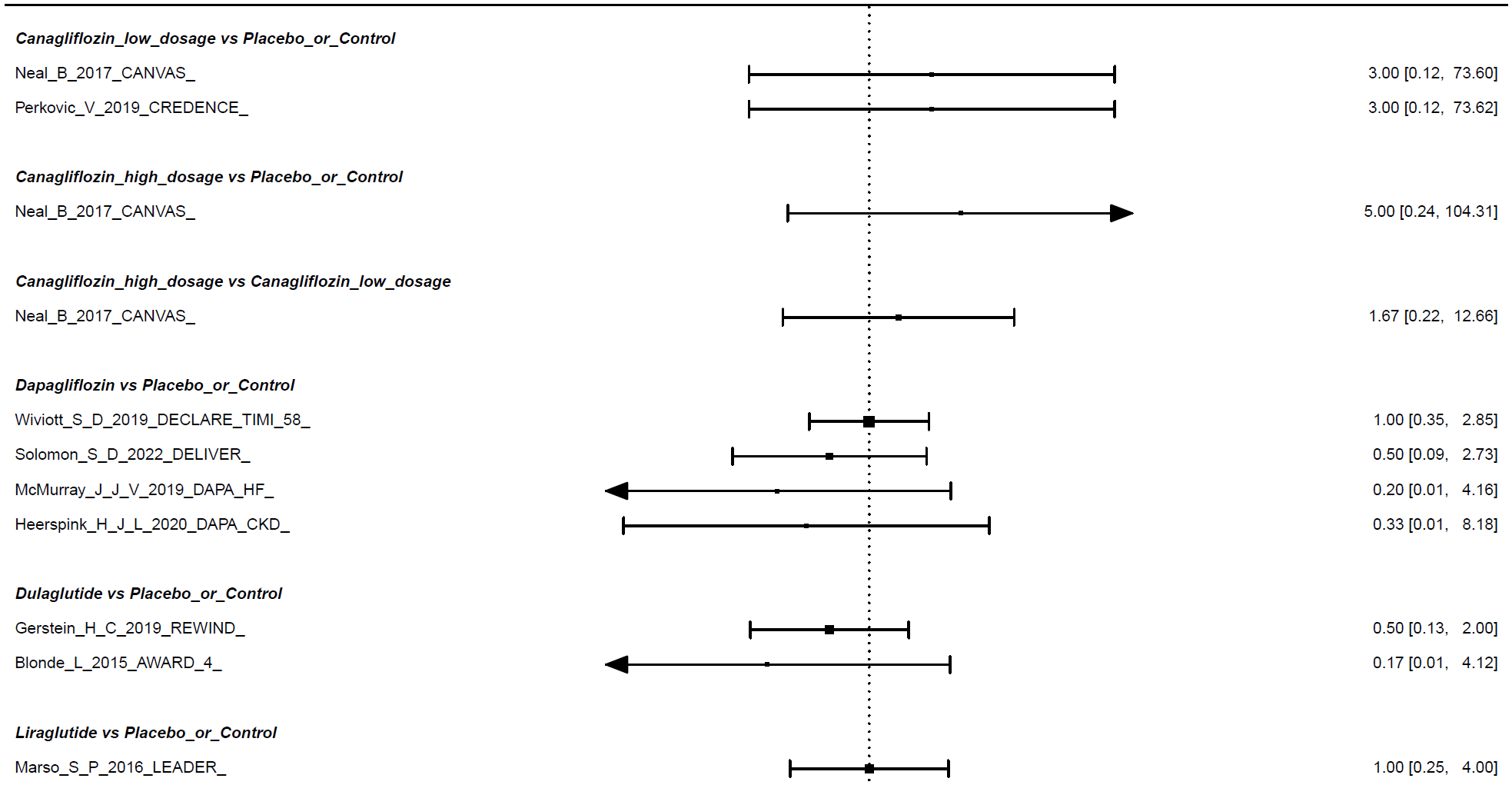
**

**
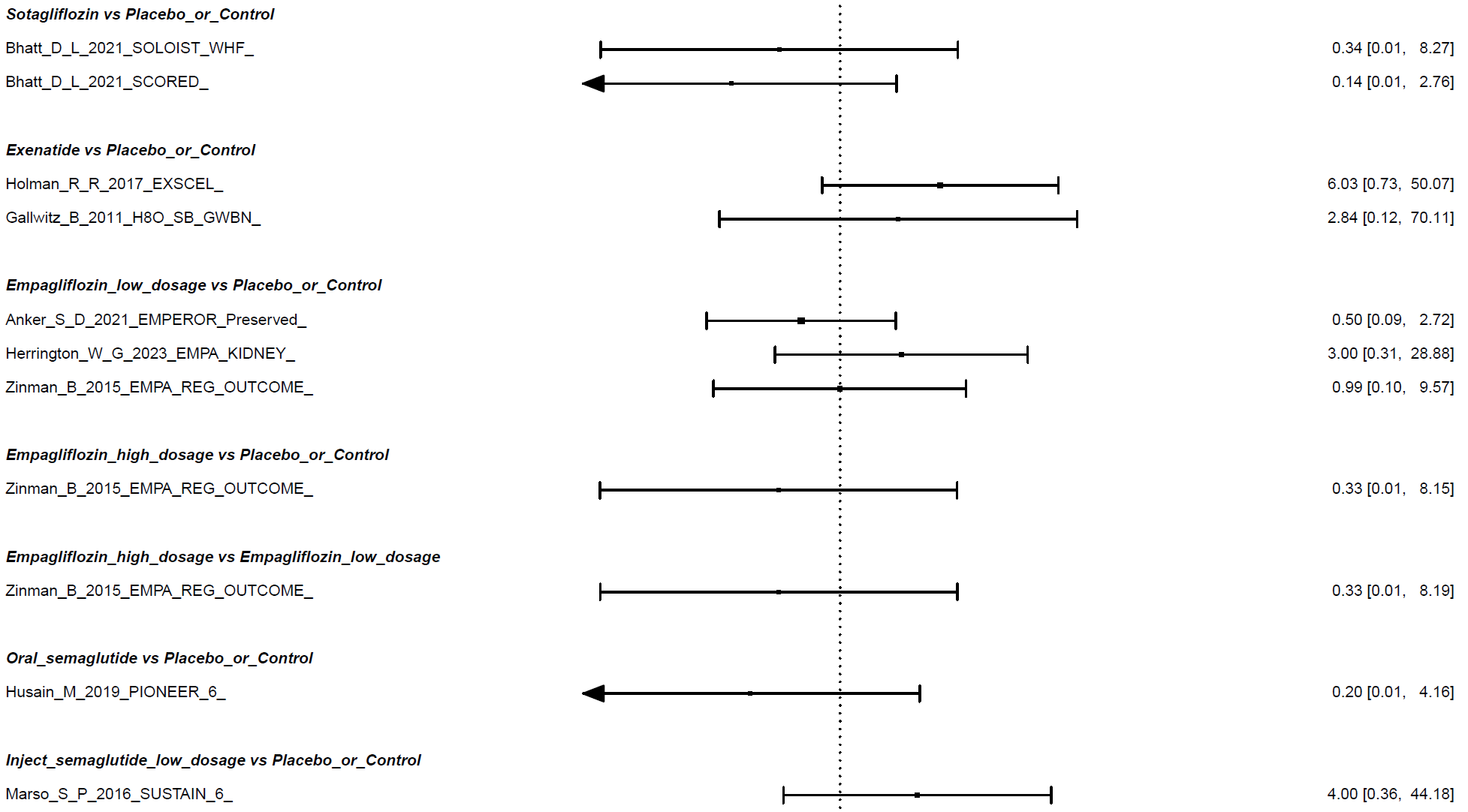
**

**
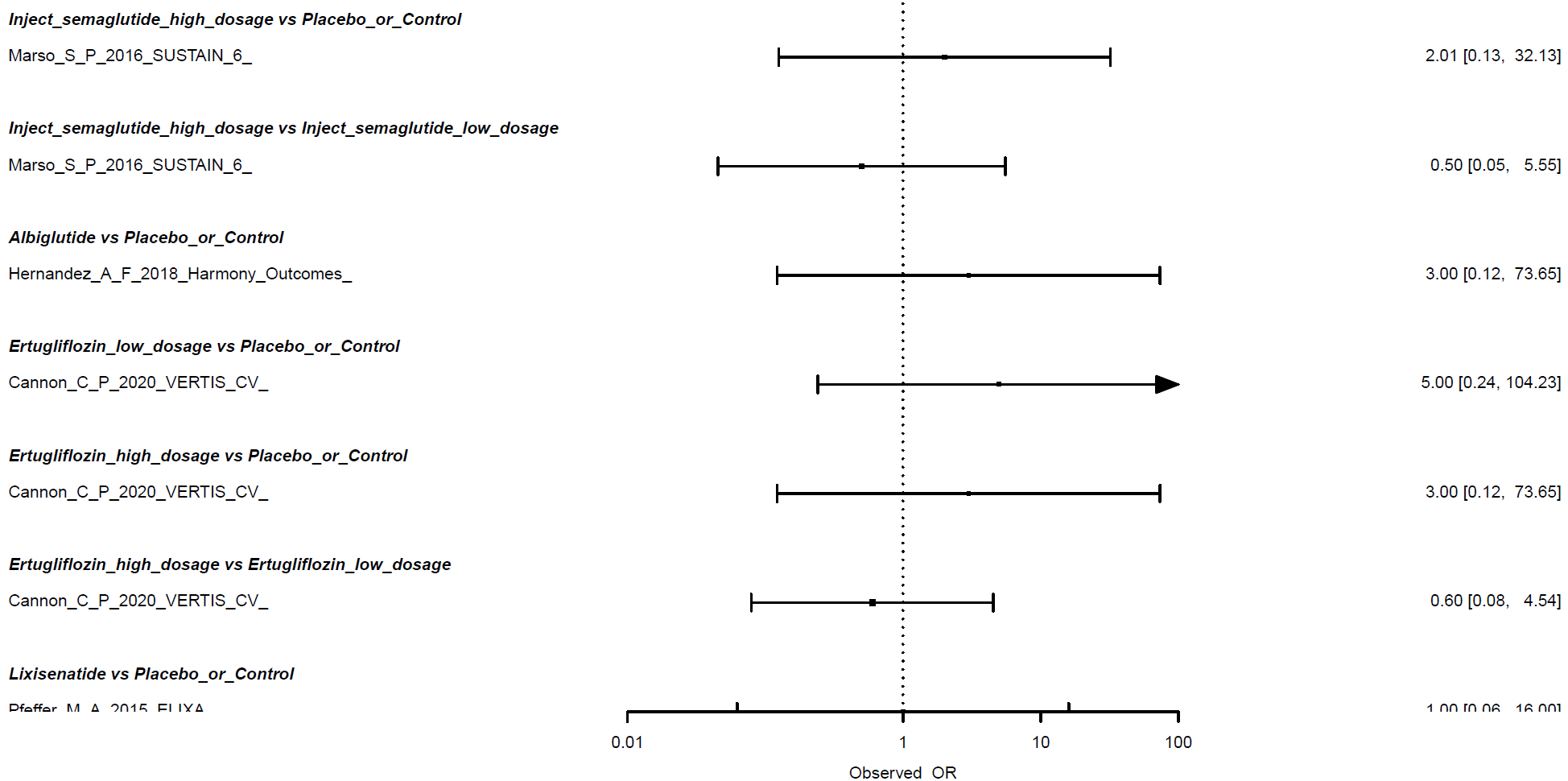
**

***Abbreviation for Fig. S4:***

*95%CIs: 95% confidence intervals; GLP-1 agonist: glucagon-like peptide-1 agonist; NMA: network meta-analysis; OR: odds ratio; RCT: randomized controlled trial; SGLT2 inhibitor: sodium–glucose cotransporter 2 inhibitor*

*Dosage definition: Canagliflozin (Low: 100mg, and High: 300mg); Ertugliflozin (Low: 5mg, and High: 15mg); Injectable semaglutide (Low: 0.5mg, and High: 1.0mg); Empagliflozin (Low: 1-10mg, and High: 25-50mg).*

**Fig. S5 Bayesian-based forest plot of primary outcome: overall events of neurodegenerative diseases**

**
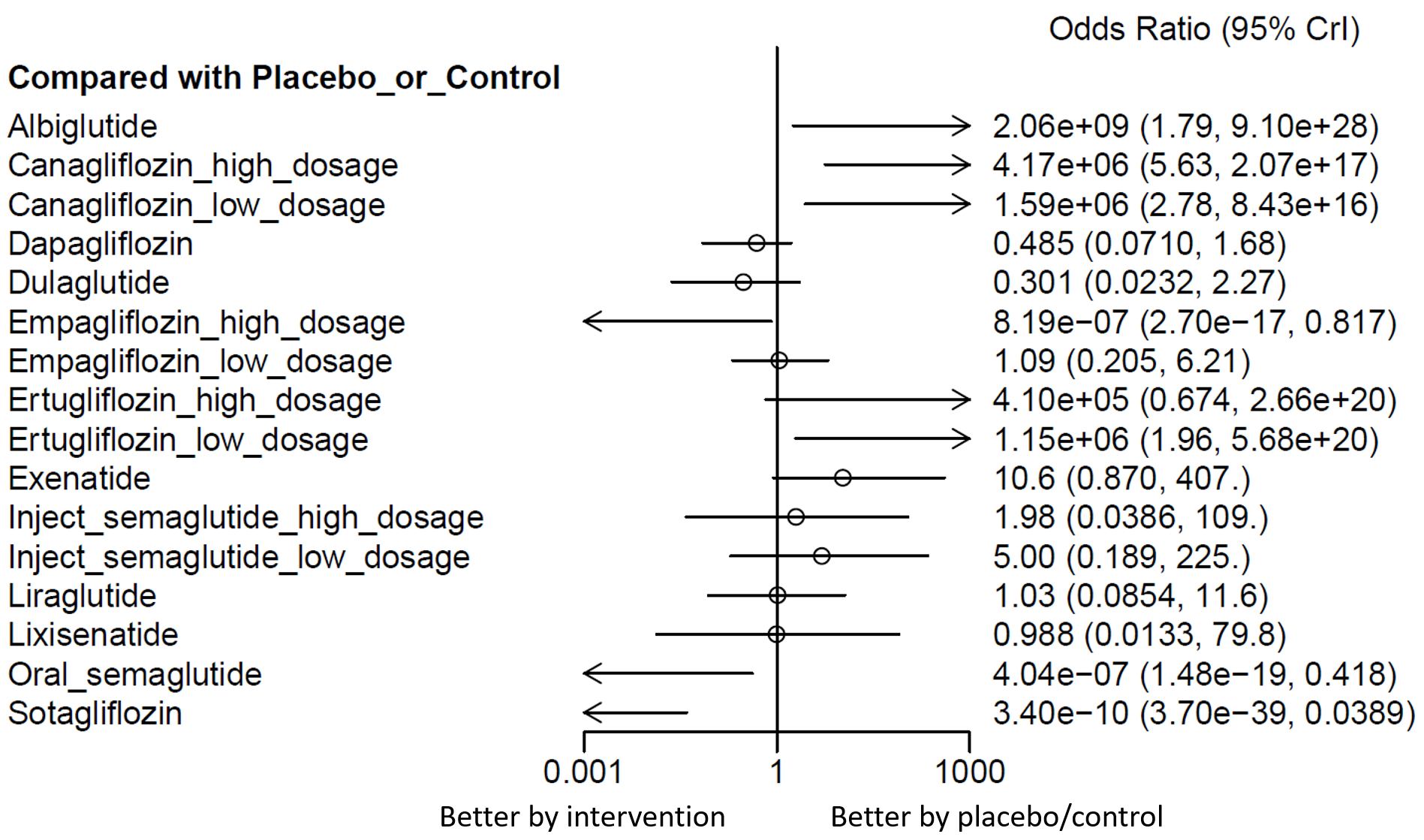
**

***Abbreviation for Fig. S5:***

*95%CIs: 95% confidence intervals; GLP-1 agonist: glucagon-like peptide-1 agonist; NMA: network meta-analysis; OR: odds ratio; RCT: randomized controlled trial; SGLT2 inhibitor: sodium–glucose cotransporter 2 inhibitor*

*Dosage definition: Canagliflozin (Low: 100mg, and High: 300mg); Ertugliflozin (Low: 5mg, and High: 15mg); Injectable semaglutide (Low: 0.5mg, and High: 1.0mg); Empagliflozin (Low: 1-10mg, and High: 25-50mg).*

**Fig. S6A Bayesian-based Litmus Rank-O-Gram rank plot of primary outcome: overall events of neurodegenerative diseases**

**
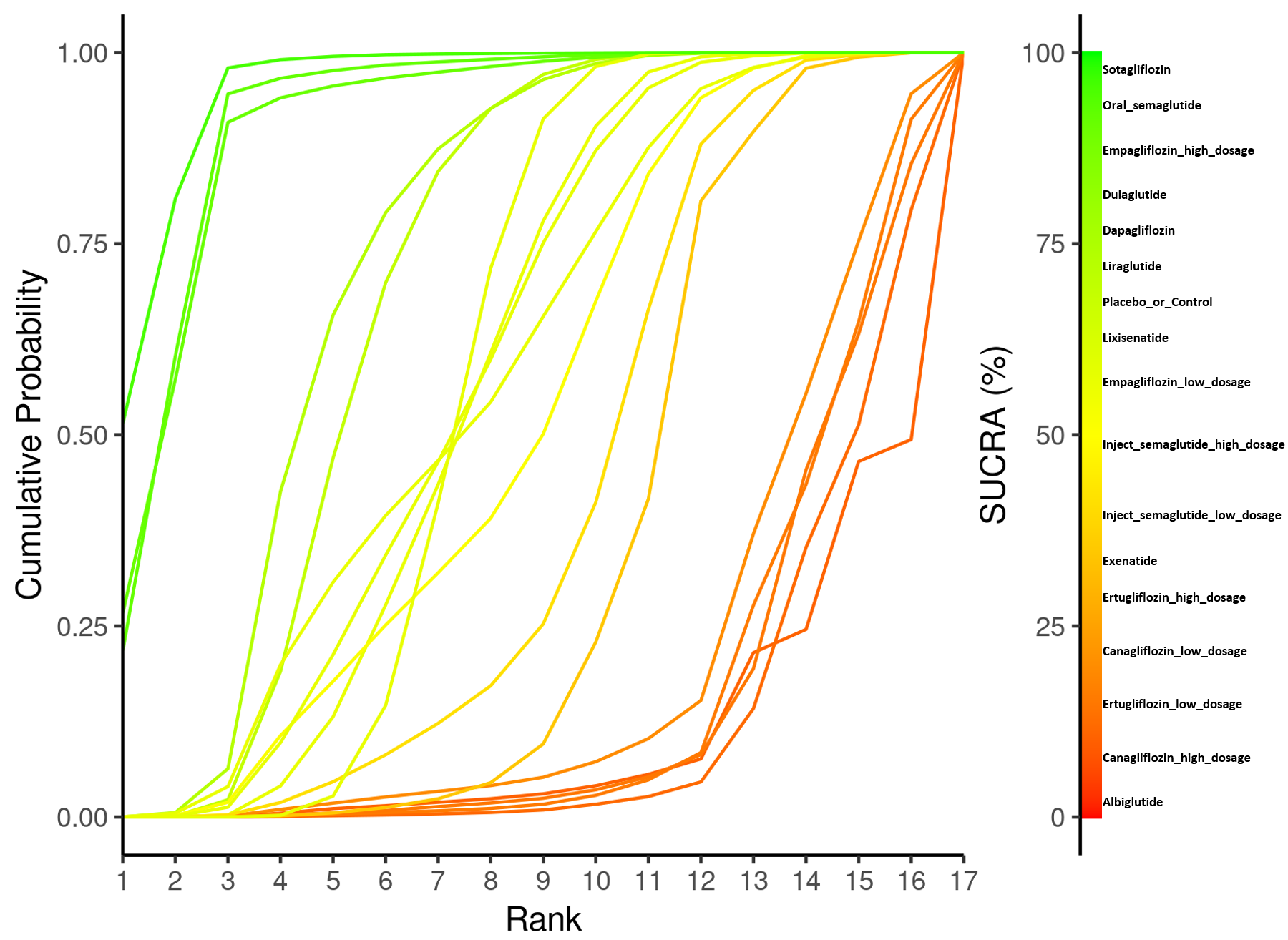
**

**Fig. S6B Bayesian-based radial surface under the cumulative ranking of primary outcome: overall events of neurodegenerative diseases**

**
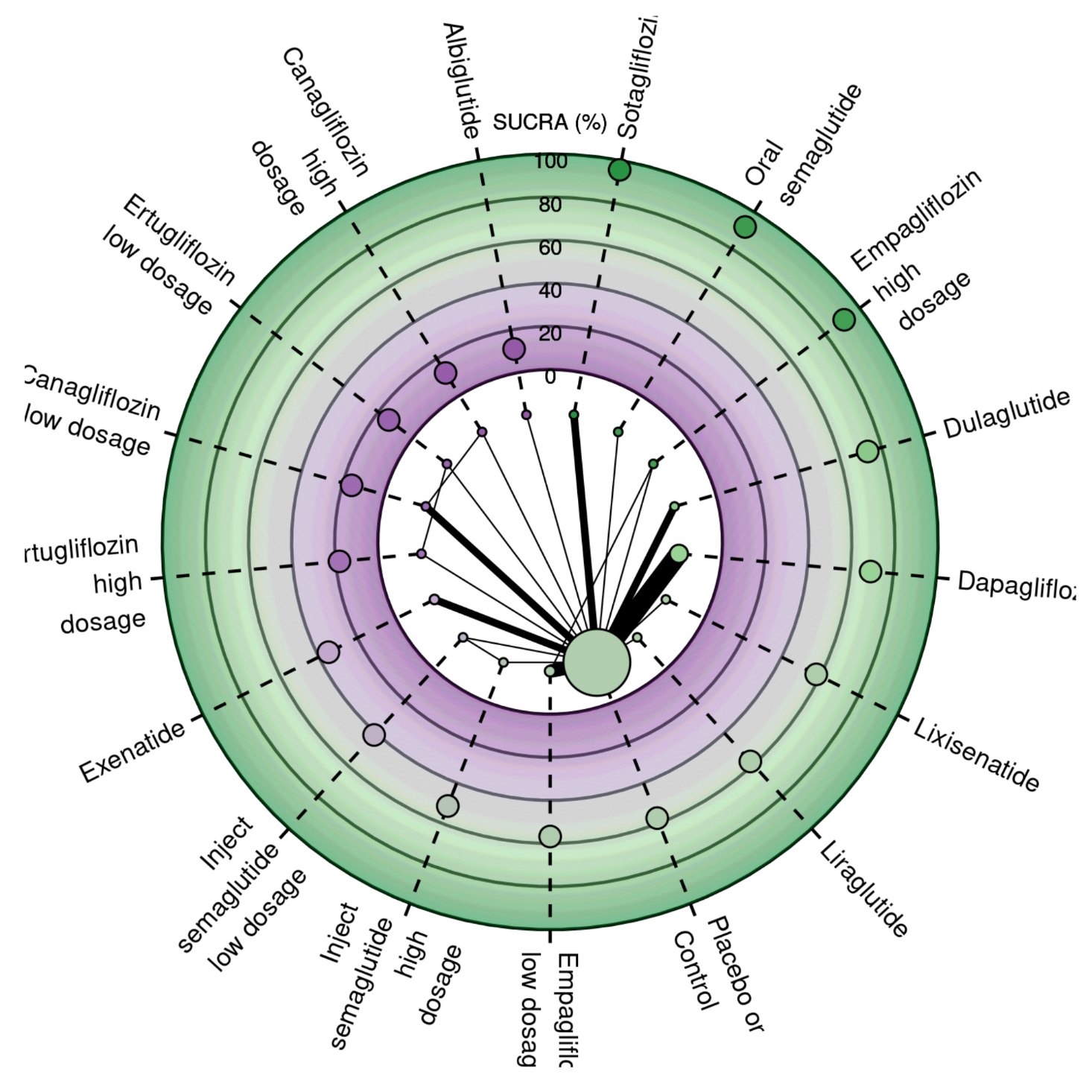
**

***Abbreviation for Fig. S6A-S6B:***

*95%CIs: 95% confidence intervals; GLP-1 agonist: glucagon-like peptide-1 agonist; NMA: network meta-analysis; OR: odds ratio; RCT: randomized controlled trial; SGLT2 inhibitor: sodium–glucose cotransporter 2 inhibitor*

*Dosage definition: Canagliflozin (Low: 100mg, and High: 300mg); Ertugliflozin (Low: 5mg, and High: 15mg); Injectable semaglutide (Low: 0.5mg, and High: 1.0mg); Empagliflozin (Low: 1-10mg, and High: 25-50mg).*

**Fig. S7A Bayesian-based residual deviance NMA/UME model of primary outcome: overall events of neurodegenerative diseases**

**
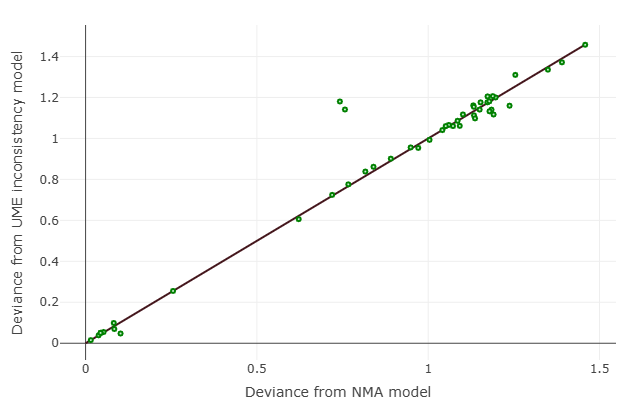
**

**Fig. S7B Bayesian-based per-arm residual deviance of primary outcome: overall events of neurodegenerative diseases**

**
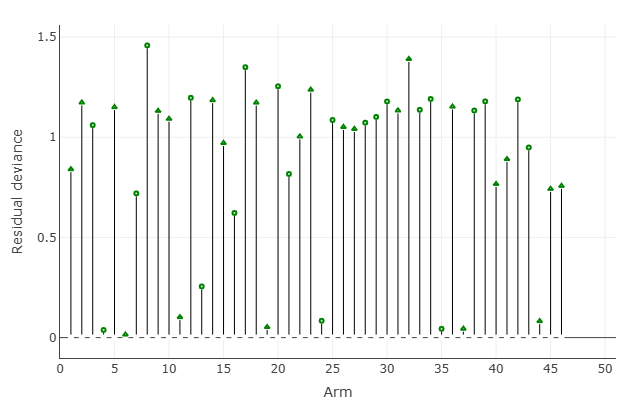
**

**Fig. S7C Bayesian-based leverage plot of primary outcome: overall events of neurodegenerative diseases**

**
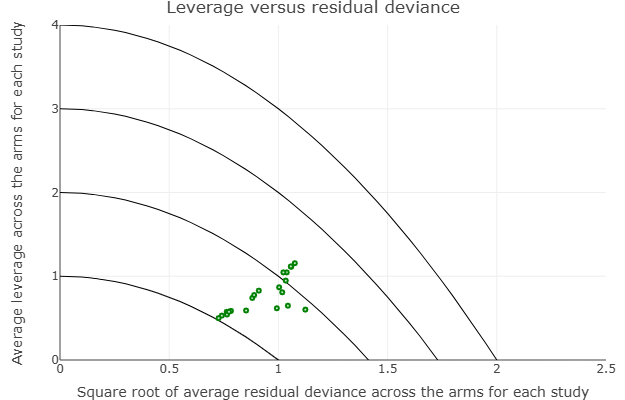
**

***Abbreviation for Fig. S7A-7C:***

*95%CIs: 95% confidence intervals; GLP-1 agonist: glucagon-like peptide-1 agonist; NMA: network meta-analysis; OR: odds ratio; RCT: randomized controlled trial; SGLT2 inhibitor: sodium–glucose cotransporter 2 inhibitor*

*Dosage definition: Canagliflozin (Low: 100mg, and High: 300mg); Ertugliflozin (Low: 5mg, and High: 15mg); Injectable semaglutide (Low: 0.5mg, and High: 1.0mg); Empagliflozin (Low: 1-10mg, and High: 25-50mg).*

**Fig. S8: detailed risk of bias in each study**

**
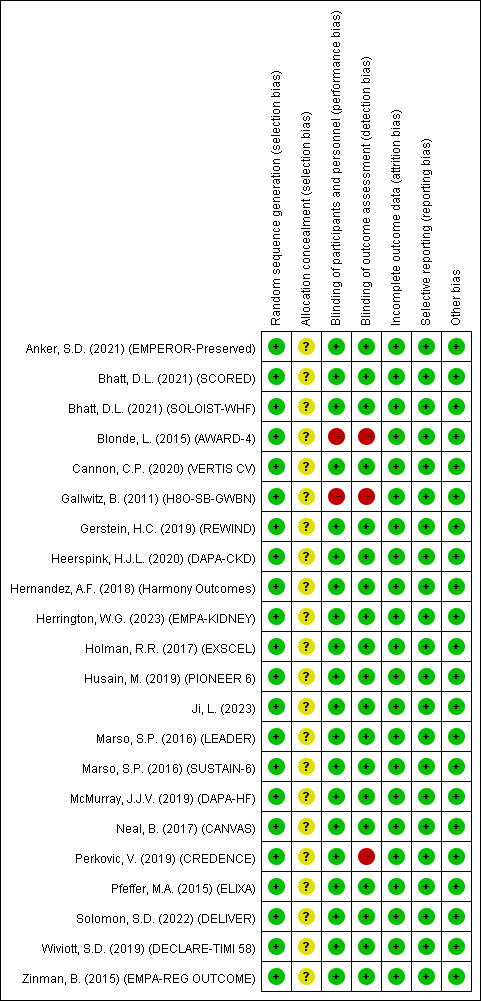
**

**Tab. S1A:** PRISMA 2020 checklist of the current network meta-analysis

| **Section and Topic** | **Item #** | **Checklist item** | **Page where item is reported** |
| --- | --- | --- | --- |
| **TITLE** | | |  |
| Title | 1 | Identify the report as a systematic review. | 1 |
| **ABSTRACT** | | |  |
| Abstract | 2 | See the PRISMA 2020 for Abstracts checklist. | 7-8 |
| **INTRODUCTION** | | |  |
| Rationale | 3 | Describe the rationale for the review in the context of existing knowledge. | 9-10 |
| Objectives | 4 | Provide an explicit statement of the objective(s) or question(s) the review addresses. | 9-10 |
| **METHODS** | | |  |
| Eligibility criteria | 5 | Specify the inclusion and exclusion criteria for the review and how studies were grouped for the syntheses. | 11-12 |
| Information sources | 6 | Specify all databases, registers, websites, organisations, reference lists and other sources searched or consulted to identify studies. Specify the date when each source was last searched or consulted. | 11-12 |
| Search strategy | 7 | Present the full search strategies for all databases, registers and websites, including any filters and limits used. | 11-12 |
| Selection process | 8 | Specify the methods used to decide whether a study met the inclusion criteria of the review, including how many reviewers screened each record and each report retrieved, whether they worked independently, and if applicable, details of automation tools used in the process. | 11-12 |
| Data collection process | 9 | Specify the methods used to collect data from reports, including how many reviewers collected data from each report, whether they worked independently, any processes for obtaining or confirming data from study investigators, and if applicable, details of automation tools used in the process. | 11-12 |
| Data items | 10a | List and define all outcomes for which data were sought. Specify whether all results that were compatible with each outcome domain in each study were sought (e.g. for all measures, time points, analyses), and if not, the methods used to decide which results to collect. | 12-13 |
|  | 10b | List and define all other variables for which data were sought (e.g. participant and intervention characteristics, funding sources). Describe any assumptions made about any missing or unclear information. | 12-13 |
| Study risk of bias assessment | 11 | Specify the methods used to assess risk of bias in the included studies, including details of the tool(s) used, how many reviewers assessed each study and whether they worked independently, and if applicable, details of automation tools used in the process. | 12-13 |
| Effect measures | 12 | Specify for each outcome the effect measure(s) (e.g. risk ratio, mean difference) used in the synthesis or presentation of results. | 12-13 |
| Synthesis methods | 13a | Describe the processes used to decide which studies were eligible for each synthesis (e.g. tabulating the study intervention characteristics and comparing against the planned groups for each synthesis (item #5)). | 12-13 |
|  | 13b | Describe any methods required to prepare the data for presentation or synthesis, such as handling of missing summary statistics, or data conversions. | 13-15 |
|  | 13c | Describe any methods used to tabulate or visually display results of individual studies and syntheses. | 13-15 |
|  | 13d | Describe any methods used to synthesize results and provide a rationale for the choice(s). If meta-analysis was performed, describe the model(s), method(s) to identify the presence and extent of statistical heterogeneity, and software package(s) used. | 13-15 |
|  | 13e | Describe any methods used to explore possible causes of heterogeneity among study results (e.g. subgroup analysis, meta-regression). | 13-15 |
|  | 13f | Describe any sensitivity analyses conducted to assess robustness of the synthesized results. | 13-15 |
| Reporting bias assessment | 14 | Describe any methods used to assess risk of bias due to missing results in a synthesis (arising from reporting biases). | 13-15 |
| Certainty assessment | 15 | Describe any methods used to assess certainty (or confidence) in the body of evidence for an outcome. | 13-15 |
| **RESULTS** | | |  |
| Study selection | 16a | Describe the results of the search and selection process, from the number of records identified in the search to the number of studies included in the review, ideally using a flow diagram. | 16-17, Fig 1, eTab 2 |
|  | 16b | Cite studies that might appear to meet the inclusion criteria, but which were excluded, and explain why they were excluded. | 16-17, eTab 3 |
| Study characteristics | 17 | Cite each included study and present its characteristics. | 16-17, eTab 4 |
| Risk of bias in studies | 18 | Present assessments of risk of bias for each included study. | 16-17, eFig 8 |
| Results of individual studies | 19 | For all outcomes, present, for each study: (a) summary statistics for each group (where appropriate) and (b) an effect estimate and its precision (e.g. confidence/credible interval), ideally using structured tables or plots. | 16-17, eFig 4 |
| Results of syntheses | 20a | For each synthesis, briefly summarise the characteristics and risk of bias among contributing studies. | 17-18, Fig 2 |
|  | 20b | Present results of all statistical syntheses conducted. If meta-analysis was done, present for each the summary estimate and its precision (e.g. confidence/credible interval) and measures of statistical heterogeneity. If comparing groups, describe the direction of the effect. | 17-18, Fig 3 |
|  | 20c | Present results of all investigations of possible causes of heterogeneity among study results. | 17-18, eTab 7 |
|  | 20d | Present results of all sensitivity analyses conducted to assess the robustness of the synthesized results. | 17-18 |
| Reporting biases | 21 | Present assessments of risk of bias due to missing results (arising from reporting biases) for each synthesis assessed. | 17-18, eFig 8 |
| Certainty of evidence | 22 | Present assessments of certainty (or confidence) in the body of evidence for each outcome assessed. | 17-18 |
| **DISCUSSION** | | |  |
| Discussion | 23a | Provide a general interpretation of the results in the context of other evidence. | 19-21 |
|  | 23b | Discuss any limitations of the evidence included in the review. | 21-22 |
|  | 23c | Discuss any limitations of the review processes used. | 21-22 |
|  | 23d | Discuss implications of the results for practice, policy, and future research. | 23 |
| **OTHER INFORMATION** | | |  |
| Registration and protocol | 24a | Provide registration information for the review, including register name and registration number, or state that the review was not registered. | 8 |
|  | 24b | Indicate where the review protocol can be accessed, or state that a protocol was not prepared. | 8 |
|  | 24c | Describe and explain any amendments to information provided at registration or in the protocol. | 8 |
| Support | 25 | Describe sources of financial or non-financial support for the review, and the role of the funders or sponsors in the review. | 24 |
| Competing interests | 26 | Declare any competing interests of review authors. | 24 |
| Availability of data, code and other materials | 27 | Report which of the following are publicly available and where they can be found: template data collection forms; data extracted from included studies; data used for all analyses; analytic code; any other materials used in the review. | 24 |

The current checklist followed the latest PRISMA 2020 guideline [187].

**Tab. S1B:** PRISMA 2020 abstract checklist of the current network meta-analysis

| **Section and Topic** | **Item #** | **Checklist item** | **Reported (Yes/No)** |
| --- | --- | --- | --- |
| **TITLE** | | |  |
| Title | 1 | Identify the report as a systematic review. | Yes |
| **BACKGROUND** | | |  |
| Objectives | 2 | Provide an explicit statement of the main objective(s) or question(s) the review addresses. | Yes |
| **METHODS** | | |  |
| Eligibility criteria | 3 | Specify the inclusion and exclusion criteria for the review. | Yes |
| Information sources | 4 | Specify the information sources (e.g. databases, registers) used to identify studies and the date when each was last searched. | Yes |
| Risk of bias | 5 | Specify the methods used to assess risk of bias in the included studies. | Yes |
| Synthesis of results | 6 | Specify the methods used to present and synthesise results. | Yes |
| **RESULTS** | | |  |
| Included studies | 7 | Give the total number of included studies and participants and summarise relevant characteristics of studies. | Yes |
| Synthesis of results | 8 | Present results for main outcomes, preferably indicating the number of included studies and participants for each. If meta-analysis was done, report the summary estimate and confidence/credible interval. If comparing groups, indicate the direction of the effect (i.e. which group is favoured). | Yes |
| **DISCUSSION** | | |  |
| Limitations of evidence | 9 | Provide a brief summary of the limitations of the evidence included in the review (e.g. study risk of bias, inconsistency and imprecision). | Yes |
| Interpretation | 10 | Provide a general interpretation of the results and important implications. | Yes |
| **OTHER** | | |  |
| Funding | 11 | Specify the primary source of funding for the review. | Yes |
| Registration | 12 | Provide the register name and registration number. | Yes |

**Tab. S2: Keyword used in each database and search results**

| Database | Keyword | Filter | Date | Result |
| --- | --- | --- | --- | --- |
| PubMed | (dementia OR Alzheimer OR Huntington OR multiple sclerosis OR amyotrophic lateral sclerosis OR Parkinson's disease OR Parkinsonism OR paralysis agitans) AND (glucagon-like peptide-1 receptor agonist OR Sodium Glucose Cotransporter 2 Inhibitor OR lixisenatide OR orforglipron OR exenatide OR semaglutide OR liraglutide OR albiglutide OR dulaglutide OR tirzepatide OR bexagliflozin OR canagliflozin OR dapagliflozin OR empagliflozin OR ertugliflozin OR ipragliflozin OR luseogliflozin OR remogliflozin OR sergliflozin OR sotagliflozin OR tofogliflozin OR henagliflozin OR janagliflozin OR mizagliflozin OR velagliflozin OR enavogliflozin OR licogliflozin OR rongliflozin) AND (random OR randomized OR randomised) | N/A | 2024/10/24 | 92 |
| ClinicalKey | (dementia OR Alzheimer OR Huntington OR multiple sclerosis OR amyotrophic lateral sclerosis OR Parkinson's disease OR Parkinsonism OR paralysis agitans) AND (glucagon-like peptide-1 receptor agonist OR Sodium Glucose Cotransporter 2 Inhibitor OR lixisenatide OR orforglipron OR exenatide OR semaglutide OR liraglutide OR albiglutide OR dulaglutide OR tirzepatide OR bexagliflozin OR canagliflozin OR dapagliflozin OR empagliflozin OR ertugliflozin OR ipragliflozin OR luseogliflozin OR remogliflozin OR sergliflozin OR sotagliflozin OR tofogliflozin OR henagliflozin OR janagliflozin OR mizagliflozin OR velagliflozin OR enavogliflozin OR licogliflozin OR rongliflozin) AND (random OR randomized OR randomised) | N/A | 2024/10/24 | 796 |
| Embase | (Alzheimer OR Huntington OR multiple sclerosis OR amyotrophic lateral sclerosis OR Parkinson OR dementia) AND (glucagon-like peptide-1 receptor agonist OR Sodium Glucose Cotransporter 2 Inhibitor) AND (random OR randomized OR randomised) | N/A | 2024/10/24 | 375 |
| Cochrane CENTRAL | (dementia OR Alzheimer OR Huntington OR multiple sclerosis OR amyotrophic lateral sclerosis OR Parkinson's disease OR Parkinsonism OR paralysis agitans) AND (glucagon-like peptide-1 receptor agonist OR Sodium Glucose Cotransporter 2 Inhibitor OR lixisenatide OR orforglipron OR exenatide OR semaglutide OR liraglutide OR albiglutide OR dulaglutide OR tirzepatide OR bexagliflozin OR canagliflozin OR dapagliflozin OR empagliflozin OR ertugliflozin OR ipragliflozin OR luseogliflozin OR remogliflozin OR sergliflozin OR sotagliflozin OR tofogliflozin OR henagliflozin OR janagliflozin OR mizagliflozin OR velagliflozin OR enavogliflozin OR licogliflozin OR rongliflozin) AND (random OR randomized OR randomised) | N/A | 2024/10/24 | 83 |
| ProQuest | (Alzheimer OR Huntington OR multiple sclerosis OR amyotrophic lateral sclerosis OR Parkinson OR dementia) AND (glucagon-like peptide-1 receptor agonist OR Sodium Glucose Cotransporter 2 Inhibitor) AND (random OR randomized OR randomised) | N/A | 2024/10/24 | 2772 |
| ScienceDirect | (Alzheimer OR Huntington OR multiple sclerosis OR amyotrophic lateral sclerosis OR Parkinson OR dementia) AND (glucagon-like peptide-1 receptor agonist OR Sodium Glucose Cotransporter 2 Inhibitor) AND (random) | N/A | 2024/10/24 | 3229 |
| Web of Science | (Alzheimer OR Huntington OR multiple sclerosis OR amyotrophic lateral sclerosis OR Parkinson OR dementia) AND (glucagon-like peptide-1 receptor agonist OR Sodium Glucose Cotransporter 2 Inhibitor) AND (random OR randomized OR randomised) | N/A | 2024/10/24 | 39 |
| ClinicalTrials.gov | (dementia OR Alzheimer OR Huntington OR multiple sclerosis OR amyotrophic lateral sclerosis OR Parkinson's disease OR Parkinsonism OR paralysis agitans) AND (glucagon-like peptide-1 receptor agonist OR Sodium Glucose Cotransporter 2 Inhibitor OR lixisenatide OR orforglipron OR exenatide OR semaglutide OR liraglutide OR albiglutide OR dulaglutide OR tirzepatide OR bexagliflozin OR canagliflozin OR dapagliflozin OR empagliflozin OR ertugliflozin OR ipragliflozin OR luseogliflozin OR remogliflozin OR sergliflozin OR sotagliflozin OR tofogliflozin OR henagliflozin OR janagliflozin OR mizagliflozin OR velagliflozin OR enavogliflozin OR licogliflozin OR rongliflozin) AND (random OR randomized OR randomised) | N/A | 2024/10/24 | 9 |

Abbreviation: N/A: not applied

**Tab. S3: Excluded studies and reason**

| Reason | Numbers | References |
| --- | --- | --- |
| Animal study | 9 | [8, 9, 75, 77, 84, 89, 92, 112, 138] |
| Commentary | 1 | [115] |
| Duplicate sample source with other included studies | 2 | [121, 137] |
| Meta-analysis/network meta-analysis | 9 | [13, 22, 23, 26-28, 31-33] |
| Not randomized controlled trial | 8 | [114, 118, 126, 154, 155, 167, 173, 176] |
| After checking full-text, not report target outcome, either in primary/secondary outcome or in adverse event profile | 81 | [50, 51, 53, 54, 61, 63, 74, 76, 78, 85-88, 90, 91, 93-96, 98-106, 108-111, 113, 116, 117, 119, 120, 122-125, 127, 128, 130, 132-136, 139-153, 156-161, 163-166, 168-172, 174, 175] |
| Patients had pre-existed neurodegeneration diseases at baseline | 13 | [10-12, 16, 79-83, 97, 107, 129, 131] |
| Protocol but not result of a trial | 1 | [162] |
| Review article | 9 | [7, 34-41] |

**Tab. S4: Characteristics of the included studies**

| Study name | Baseline illness | Primary endpoint | Glycemic outcome | Comparison | Number | Mean age (year) | Female (%) | Treatment duration | Category | ClinicalTrials.gov | Country |
| --- | --- | --- | --- | --- | --- | --- | --- | --- | --- | --- | --- |
| Herrington, W.G. (2023) (EMPA-KIDNEY)[64] | patients with renal failure | Composite of progression of kidney disease or death from cardiovascular causes | NA | Empagliflozin 10mg/day Placebo | 3304 3305 | 63.9±13.9 63.8±13.9 | 33.2 33.1 | 104 weeks | SGLT2 inhibitor | NCT03594110 | Multiple countries |
| Ji, L. (2023)[57] | patients with type 2 diabetes mellitus | Change from baseline in HbA1c | HbA1c:  -1.12(0.10)  -1.12(0.10)  -0.13(0.10) | Empagliflozin 10mg/day Empagliflozin 25mg/day Placebo | 73 73 73 | 59.9±7.7 60.7±9.1 60.1±8.0 | 41.1 45.2 50.7 | 24 weeks | SGLT2 inhibitor | NCT04233801 | China |
| Solomon, S.D. (2022) (DELIVER)[20] | patients with stabilized heart failure | Composite of worsening heart failure or cardiovascular death | NA | Dapagliflozin 10mg/day Placebo | 3131 3132 | 71.8±9.6 71.5±9.5 | 43.6 44.2 | 120 weeks | SGLT2 inhibitor | NCT03619213 | Multiple countries |
| Anker, S.D. (2021) (EMPEROR-Preserved)[47] | patients with heart failure with preserved ejection fraction | Composite of cardiovascular death or hospitalization for heart failure | HbA1c:  -0.16±0.02  0.03±0.02 | Empagliflozin 10mg/day Placebo | 2997 2991 | 71.8±9.3 71.9±9.6 | 44.6 44.7 | 156 weeks | SGLT2 inhibitor | NCT03057951 | Multiple countries |
| Bhatt, D.L. (2021) (SCORED)[48] | patients with type 2 diabetes mellitus and chronic kidney disease | Total number of deaths from cardiovascular causes, hospitalizations for heart failure, and urgent visits for heart failure | HbA1c:  -0.60 (-0.63, -0.56)  -0.17 (-0.21, -0.14) | Sotagliflozin 200-400mg/day Placebo | 5292 5292 | 68.4±8.4 68.2±8.4 | 44.3 45.5 | 116 weeks | SGLT2 inhibitor | NCT03315143 | Multiple countries |
| Bhatt, D.L. (2021) (SOLOIST-WHF)[49] | patients with type 2 diabetes mellitus and heart failure | Total number of deaths from cardiovascular causes and hospitalizations and urgent visits for heart failure | NA | Sotagliflozin 200mg/day Placebo | 608 614 | 68.6±9.5 69.3±8.8 | 32.6 34.9 | 36 weeks | SGLT2 inhibitor | NCT03521934 | Multiple countries |
| Cannon, C.P. (2020) (VERTIS CV)[51] | patients with type 2 diabetes mellitus | Major adverse cardiovascular events | HbA1c:  -0.70%  -0.72%  -0.22% | Ertugliflozin 5 mg/day Ertugliflozin 15 mg/day Placebo | 2746 2747 2745 | 64.3±8.2 64.4±8.0 64.4±8.0 | 29.1 30.3 30.7 | 182 weeks | SGLT2 inhibitor | NCT01986881 | Multiple countries |
| Heerspink, H.J.L. (2020) (DAPA-CKD)[53] | patients with renal failure | Composite of a sustained decline in the estimated GFR, end-stage kidney disease, or death from renal or cardiovascular causes | NA | Dapagliflozin 10mg/day Placebo | 2149 2149 | 61.8±12.1 61.9±12.1 | 32.9 33.3 | 125 weeks | SGLT2 inhibitor | NCT03036150 | Multiple countries |
| Gerstein, H.C. (2019) (REWIND)[21] | patients with type 2 diabetes mellitus | First occurrence of the composite end point of non-fatal myocardial infarction, non-fatal stroke, or death from cardiovascular causes | HbA1c:  -0.46±0.01  0.16±0.01 | Dulaglutide 1.5 mg/week Placebo | 4949 4952 | 66.2±6.5 66.2±6.5 | 46.6 46.1 | 281 weeks | GLP-1 agonist | NCT01394952 | Multiple countries |
| Husain, M. (2019) (PIONEER 6)[56] | patients with cardiovascular disease or chronic kidney disease | First occurrence of a major adverse cardiovascular event | HbA1c:  -1.0  -0.3 | Semaglutide 14mg/day (oral) Placebo | 1591 1592 | 66.0±7.0 66.0±7.0 | 31.9 31.4 | 64 weeks | GLP-1 agonist | NCT02692716 | Multiple countries |
| McMurray, J.J.V. (2019) (DAPA-HF)[60] | patients with stabilized heart failure | Composite of worsening heart failure or cardiovascular death | HbA1c:  -0.21±1.14  0.04±1.29 | Dapagliflozin 10mg/day Placebo | 2373 2371 | 66.2±11.0 66.5±10.8 | 23.8 23.0 | 73 weeks | SGLT2 inhibitor | NCT03036124 | Multiple countries |
| Perkovic, V. (2019) (CREDENCE)[62] | patients with type 2 diabetes mellitus | Composite of end-stage kidney disease, a doubling of the serum creatinine level, or death from renal or cardiovascular causes | HbA1c:  -0.43%  -0.32% | Canagliflozin 100 mg/day Placebo | 2202 2199 | 62.9±9.2 63.2±9.2 | 34.6 33.3 | 130 weeks | SGLT2 inhibitor | NCT02065791 | Multiple countries |
| Wiviott, S.D. (2019) (DECLARE-TIMI 58)[19] | patients with atherosclerotic vascular disease | Major adverse cardiovascular events and a composite of cardiovascular death or hospitalization for heart failure. | HbA1c:  -0.4%  -0.2% | Dapagliflozin 10mg/day Placebo | 8582 8578 | 63.9±6.8 64.0±6.8 | 36.9 37.9 | 206 weeks | SGLT2 inhibitor | NCT01730534 | Multiple countries |
| Hernandez, A.F. (2018) (Harmony Outcomes)[54] | patients with type 2 diabetes mellitus | First occurrence of cardiovascular death, myocardial infarction, or stroke | HbA1c:  Between group difference -0.52 (95%CIs=-0.58 to -0.45) | Albiglutide 30-50 mg/week Placebo | 4717 4715 | 64.1±8.7 64.2±8.7 | 30.2 31.0 | 86 weeks | GLP-1 agonist | NCT02465515 | Multiple countries |
| Holman, R.R. (2017) (EXSCEL)[55] | patients with type 2 diabetes mellitus | First occurrence of death from cardiovascular causes, nonfatal myocardial infarction, or nonfatal stroke | HbA1c:  Between group difference -0.7 (95%CIs=-0.7 to -0.6) | Exenatide 2mg/week Placebo | 7356 7396 | 61.8±9.4 61.9±9.4 | 38.0 38.0 | 166 weeks | GLP-1 agonist | NCT01144338 | Multiple countries |
| Neal, B. (2017) (CANVAS)[61] | patients with type 2 diabetes mellitus | Composite of death from cardiovascular causes, nonfatal myocardial infarction, or nonfatal stroke | HbA1c:  -0.26±0.032  -0.31±0.032  0.01±0.032 | Canagliflozin 100 mg/day Canagliflozin 300 mg/day Placebo | 1445 1443 1442 | 62.2±8.0 62.8±8.1 62.3±7.9 | 33.5 34.6 33.7 | 126 weeks | SGLT2 inhibitor | NCT01032629 | Multiple countries |
| Marso, S.P. (2016) (LEADER)[59] | patients with type 2 diabetes mellitus | First occurrence of death from cardiovascular causes, nonfatal myocardial infarction, or nonfatal stroke | NA | Liraglutide 1.8mg/day Placebo | 4668 4672 | 64.2±7.2 64.4±7.2 | 35.5 36.0 | 198 weeks | GLP-1 agonist | NCT01179048 | Multiple countries |
| Marso, S.P. (2016) (SUSTAIN-6)[58] | patients with type 2 diabetes mellitus | First occurrence of cardiovascular death, nonfatal myocardial infarction, or nonfatal stroke | HbA1c:  -1.1%  -1.4%  -0.4% | Inject semaglutide 0.5 mg/week Inject semaglutide 1.0 mg/week Placebo | 826 822 1649 | NA | 40.1 37.0 40.0 | 109 weeks | GLP-1 agonist | NCT01720446 | Multiple countries |
| Blonde, L. (2015) (AWARD-4)[50] | patients with type 2 diabetes mellitus | Change in HbA1c from baseline | HbA1c:  -1.61%  -1.41% | Dulaglutide 0.75-1.5mg/week Control with insulin glargine | 588 296 | 59.1±9.3 59.9±9.1 | 47.6 44.3 | 52 weeks | GLP-1 agonist | NCT01191268 | Multiple countries |
| Pfeffer, M.A. (2015) (ELIXA)[63] | patients with type 2 diabetes mellitus and recent acute coronary syndrome | Composite end point of cardiovascular death, myocardial infarction, stroke, or hospitalization for unstable angina | HbA1c:  -0.6%  -0.2% | Lixisenatide 20ug/day Placebo | 3031 3032 | 59.9±9.7 60.6±9.6 | 30.4 30.9 | 100 weeks | GLP-1 agonist | NCT01147250 | Multiple countries |
| Zinman, B. (2015) (EMPA-REG OUTCOME)[65] | patients with type 2 diabetes mellitus | Death from cardiovascular causes, nonfatal myocardial infarction, or nonfatal stroke | HbA1c:  -0.24 (10mg vs placebo)  -0.36 (25mg vs placebo) | Empagliflozin 10mg/day Empagliflozin 25mg/day Placebo | 2345 2342 2333 | 63.0±8.6 63.2±8.6 63.2±8.8 | 29.5 28.1 28.0 | 135 weeks | SGLT2 inhibitor | NCT01131676 | Multiple countries |
| Gallwitz, B. (2011) (H8O-SB-GWBN)[52] | patients with type 2 diabetes mellitus | Test exenatide BID was noninferior to premixed insulin aspart for HbA1C control | HbA1c:  -1.00(0.05)  -1.14(0.05) | Exenatide 5-10 mcg twice daily Control with insulin aspart | 181 173 | 57.2±10.0 56.9±9.9 | 40.3 44.5 | 26 weeks | GLP-1 agonist | NCT00434954 | Germany |

*Abbreviations: GLP-1 agonist: glucagon-like peptide-1 agonist; HbA1c:* g*lycated hemoglobin; NA: not available; SGLT2 inhibitor: sodium–glucose cotransporter 2 inhibitor*

**Tab. S5A: League table of primary outcome: subgroup of Alzheimer’s disease events**

| Dulaglutide | 0.50 [0.05; 5.52] | . | . | . | . | . | . | . |
| --- | --- | --- | --- | --- | --- | --- | --- | --- |
| 0.50 [0.05; 5.52] | Placebo_or_Control | 0.90 [0.17; 4.77] | 0.33 [0.01; 8.19] | 0.33 [0.01; 8.19] | 0.33 [0.01; 8.19] | 0.20 [0.01; 4.16] | 0.21 [0.02; 1.86] | 0.14 [0.01; 2.75] |
| 0.45 [0.02; 8.37] | 0.90 [0.17; 4.77] | Empagliflozin | . | . | . | . | . | . |
| 0.17 [0.00; 9.12] | 0.33 [0.01; 8.19] | 0.37 [0.01; 13.76] | Ertugliflozin_  high_dosage | . | 1.00 [0.10; 9.62] | . | . | . |
| 0.17 [0.00; 9.12] | 0.33 [0.01; 8.19] | 0.37 [0.01; 13.76] | 1.00 [0.01; 92.45] | Albiglutide | . | . | . | . |
| 0.17 [0.00; 9.12] | 0.33 [0.01; 8.19] | 0.37 [0.01; 13.75] | 1.00 [0.10; 9.62] | 1.00 [0.01; 92.45] | Ertugliflozin_  low_dosage | . | . | . |
| 0.10 [0.00; 4.80] | 0.20 [0.01; 4.16] | 0.22 [0.01; 7.13] | 0.60 [0.01; 49.39] | 0.60 [0.01; 49.39] | 0.60 [0.01; 49.41] | Liraglutide | . | . |
| 0.11 [0.00; 2.70] | 0.21 [0.02; 1.86] | 0.24 [0.02; 3.66] | 0.63 [0.01; 30.36] | 0.63 [0.01; 30.36] | 0.63 [0.01; 30.37] | 1.06 [0.03; 44.28] | Dapagliflozin | . |
| 0.07 [0.00; 3.23] | 0.14 [0.01; 2.75] | 0.16 [0.01; 4.76] | 0.43 [0.01; 33.46] | 0.43 [0.01; 33.46] | 0.43 [0.01; 33.47] | 0.71 [0.01; 49.58] | 0.67 [0.02; 26.59] | Exenatide |

Data present as OR [95%CIs]. Pairwise (upper-right portion) and network (lower-left portion) meta-analysis results are presented as estimate effect sizes for the outcome of events of Alzheimer’s disease. Interventions are reported in order of mean ranking of beneficially prophylactic effect on events of Alzheimer’s disease, and outcomes are expressed as odds ratio (OR) (95% confidence intervals) (95%CIs). For the pairwise meta-analyses, OR of less than 1 indicate that the treatment specified in the row got more beneficial effect than that specified in the column. For the network meta-analysis (NMA), OR of less than 1 indicate that the treatment specified in the column got more beneficial effect than that specified in the row.

*Dosage definition: Canagliflozin (Low: 100mg, and High: 300mg); Ertugliflozin (Low: 5mg, and High: 15mg); Injectable semaglutide (Low: 0.5mg, and High: 1.0mg); Empagliflozin (Low: 1-10mg, and High: 25-50mg).*

**Tab. S5B: League table of primary outcome: subgroup of dementia of Lewy body events**

| Oral_semaglutide | . | 0.33 [0.01; 8.18] | . |
| --- | --- | --- | --- |
| 1.00 [0.01; 92.37] | Liraglutide | 0.33 [0.01; 8.19] | . |
| 0.33 [0.01; 8.18] | 0.33 [0.01; 8.19] | Placebo_or_Control | 0.33 [0.01; 8.19] |
| 0.11 [0.00; 10.27] | 0.11 [0.00; 10.28] | 0.33 [0.01; 8.19] | Dapagliflozin |

Data present as OR [95%CIs]. Pairwise (upper-right portion) and network (lower-left portion) meta-analysis results are presented as estimate effect sizes for the outcome of events of dementia of Lewy body. Interventions are reported in order of mean ranking of beneficially prophylactic effect on events of dementia of Lewy body, and outcomes are expressed as odds ratio (OR) (95% confidence intervals) (95%CIs). For the pairwise meta-analyses, OR of less than 1 indicate that the treatment specified in the row got more beneficial effect than that specified in the column. For the network meta-analysis (NMA), OR of less than 1 indicate that the treatment specified in the column got more beneficial effect than that specified in the row.

*Dosage definition: Canagliflozin (Low: 100mg, and High: 300mg); Ertugliflozin (Low: 5mg, and High: 15mg); Injectable semaglutide (Low: 0.5mg, and High: 1.0mg); Empagliflozin (Low: 1-10mg, and High: 25-50mg).*

**Tab. S5C: League table of primary outcome: subgroup of multiple sclerosis events**

| Dapagliflozin | . | . | 0.33 [0.01; 8.18] | . |
| --- | --- | --- | --- | --- |
| 1.00 [0.01; 92.46] | Inject_semaglutide | . | 0.33 [0.01; 8.19] | . |
| 1.00 [0.01; 92.09] | 1.00 [0.01; 92.14] | Exenatide | 0.33 [0.01; 8.21] | . |
| 0.33 [0.01; 8.18] | 0.33 [0.01; 8.19] | 0.33 [0.01; 8.21] | Placebo_or_Control | 0.33 [0.01; 8.18] |
| 0.11 [0.00; 10.26] | 0.11 [0.00; 10.27] | 0.11 [0.00; 10.30] | 0.33 [0.01; 8.18] | Lixisenatide |

Data present as OR [95%CIs]. Pairwise (upper-right portion) and network (lower-left portion) meta-analysis results are presented as estimate effect sizes for the outcome of events of multiple sclerosis. Interventions are reported in order of mean ranking of beneficially prophylactic effect on events of multiple sclerosis, and outcomes are expressed as odds ratio (OR) (95% confidence intervals) (95%CIs). For the pairwise meta-analyses, OR of less than 1 indicate that the treatment specified in the row got more beneficial effect than that specified in the column. For the network meta-analysis (NMA), OR of less than 1 indicate that the treatment specified in the column got more beneficial effect than that specified in the row.

*Dosage definition: Canagliflozin (Low: 100mg, and High: 300mg); Ertugliflozin (Low: 5mg, and High: 15mg); Injectable semaglutide (Low: 0.5mg, and High: 1.0mg); Empagliflozin (Low: 1-10mg, and High: 25-50mg).*

**Tab. S5D: League table of primary outcome: subgroup of amyotrophic lateral sclerosis events**

| Dulaglutide | . | . | 0.17 [0.01; 4.12] | . | . |
| --- | --- | --- | --- | --- | --- |
| 0.50 [0.01; 46.52] | Lixisenatide | . | 0.33 [0.01; 8.19] | . | . |
| 0.17 [0.00; 11.58] | 0.33 [0.00; 23.02] | Dapagliflozin | 1.00 [0.06; 15.98] | . | . |
| 0.17 [0.01; 4.12] | 0.33 [0.01; 8.19] | 1.00 [0.06; 15.98] | Placebo_or_Control | 0.67 [0.03; 16.39] | 0.67 [0.03; 16.37] |
| 0.11 [0.00; 10.35] | 0.22 [0.00; 20.57] | 0.67 [0.01; 46.04] | 0.67 [0.03; 16.39] | Canagliflozin | . |
| 0.11 [0.00; 10.34] | 0.22 [0.00; 20.55] | 0.67 [0.01; 46.00] | 0.67 [0.03; 16.37] | 1.00 [0.01; 92.43] | Ertugliflozin |

Data present as OR [95%CIs]. Pairwise (upper-right portion) and network (lower-left portion) meta-analysis results are presented as estimate effect sizes for the outcome of events of amyotrophic lateral sclerosis. Interventions are reported in order of mean ranking of beneficially prophylactic effect on events of amyotrophic lateral sclerosis, and outcomes are expressed as odds ratio (OR) (95% confidence intervals) (95%CIs). For the pairwise meta-analyses, OR of less than 1 indicate that the treatment specified in the row got more beneficial effect than that specified in the column. For the network meta-analysis (NMA), OR of less than 1 indicate that the treatment specified in the column got more beneficial effect than that specified in the row.

*Dosage definition: Canagliflozin (Low: 100mg, and High: 300mg); Ertugliflozin (Low: 5mg, and High: 15mg); Injectable semaglutide (Low: 0.5mg, and High: 1.0mg); Empagliflozin (Low: 1-10mg, and High: 25-50mg).*

**Tab. S5E: League table of safety profile: drop-out rate**

| Canagliflozin_  high_dosage | . | 0.86 [0.58; 1.28] | . | . | . | . | . | . | . | . | . | . | . | . | ***0.58 [0.40; 0.85]** | . |
| --- | --- | --- | --- | --- | --- | --- | --- | --- | --- | --- | --- | --- | --- | --- | --- | --- |
| 1.05 [0.47; 2.36] | Inject_  semaglutide_  high_dosage | . | . | 0.79 [0.34; 1.81] | . | . | . | . | . | . | . | . | . | . | 0.55 [0.27; 1.12] | . |
| 0.88 [0.60; 1.28] | 0.83 [0.38; 1.84] | Canagliflozin_  low_dosage | . | . | . | . | . | . | . | . | . | . | . | . | ***0.65 [0.47; 0.91]** | . |
| 1.10 [0.29; 4.22] | 1.05 [0.24; 4.60] | 1.26 [0.33; 4.76] | Sotagliflozin | . | . | . | . | . | . | . | . | . | . | . | 0.52 [0.14; 1.90] | . |
| 0.83 [0.39; 1.77] | 0.79 [0.34; 1.81] | 0.94 [0.45; 1.98] | 0.75 [0.18; 3.21] | Inject_  semaglutide_  low_dosage | . | . | . | . | . | . | . | . | . | . | 0.69 [0.36; 1.35] | . |
| 0.72 [0.46; 1.12] | 0.68 [0.32; 1.46] | 0.82 [0.54; 1.24] | 0.65 [0.17; 2.43] | 0.86 [0.42; 1.77] | Empagliflozin_  high_dosage | . | . | . | 0.99 [0.75; 1.32] | . | . | . | . | . | 0.74 [0.56; 0.99] | . |
| 0.70 [0.45; 1.11] | 0.67 [0.31; 1.44] | 0.80 [0.53; 1.23] | 0.64 [0.17; 2.40] | 0.85 [0.42; 1.75] | 0.98 [0.68; 1.43] | Albiglutide | . | . | . | . | . | . | . | . | 0.81 [0.62; 1.07] | . |
| 0.69 [0.43; 1.11] | 0.66 [0.30; 1.43] | 0.79 [0.50; 1.23] | 0.63 [0.17; 2.36] | 0.83 [0.40; 1.74] | 0.96 [0.64; 1.44] | 0.98 [0.65; 1.47] | Dapagliflozin | . | . | . | . | . | . | . | 0.83 [0.61; 1.13] | . |
| 0.69 [0.19; 2.45] | 0.65 [0.16; 2.69] | 0.79 [0.22; 2.77] | 0.63 [0.11; 3.69] | 0.83 [0.21; 3.33] | 0.96 [0.28; 3.34] | 0.98 [0.28; 3.40] | 1.00 [0.28; 3.50] | Oral_  semaglutide | . | . | . | . | . | . | 0.83 [0.25; 2.81] | . |
| ***0.66 [0.44; 0.99]** | 0.63 [0.30; 1.32] | 0.76 [0.53; 1.09] | 0.60 [0.16; 2.21] | 0.80 [0.40; 1.59] | 0.93 [0.71; 1.20] | 0.94 [0.69; 1.29] | 0.96 [0.68; 1.36] | 0.96 [0.28; 3.28] | Empagliflozin_  low_dosage | . | . | . | . | . | 0.87 [0.74; 1.02] | . |
| 0.66 [0.40; 1.09] | 0.63 [0.28; 1.39] | 0.75 [0.47; 1.21] | 0.60 [0.16; 2.28] | 0.80 [0.38; 1.68] | 0.92 [0.60; 1.42] | 0.93 [0.60; 1.45] | 0.96 [0.60; 1.52] | 0.96 [0.27; 3.39] | 0.99 [0.68; 1.45] | Liraglutide | . | . | . | . | 0.87 [0.62; 1.23] | . |
| 0.63 [0.39; 1.02] | 0.60 [0.28; 1.31] | 0.72 [0.46; 1.12] | 0.57 [0.15; 2.16] | 0.76 [0.37; 1.58] | 0.88 [0.59; 1.31] | 0.90 [0.60; 1.34] | 0.92 [0.59; 1.41] | 0.92 [0.26; 3.21] | 0.95 [0.68; 1.34] | 0.96 [0.61; 1.51] | Ertugliflozin_  low_dosage | . | 0.95 [0.70; 1.28] | . | 0.91 [0.67; 1.23] | . |
| 0.60 [0.36; 1.02] | 0.57 [0.25; 1.29] | 0.69 [0.42; 1.13] | 0.55 [0.14; 2.10] | 0.73 [0.34; 1.56] | 0.84 [0.53; 1.33] | 0.85 [0.54; 1.36] | 0.87 [0.54; 1.42] | 0.87 [0.25; 3.12] | 0.91 [0.60; 1.36] | 0.91 [0.55; 1.52] | 0.95 [0.59; 1.54] | Lixisenatide | . | . | 0.95 [0.66; 1.38] | . |
| ***0.60 [0.37; 0.96]** | 0.57 [0.26; 1.24] | 0.68 [0.44; 1.06] | 0.54 [0.14; 2.04] | 0.72 [0.35; 1.50] | 0.83 [0.56; 1.24] | 0.85 [0.57; 1.27] | 0.87 [0.56; 1.33] | 0.87 [0.25; 3.03] | 0.90 [0.64; 1.27] | 0.91 [0.57; 1.43] | 0.95 [0.70; 1.28] | 0.99 [0.61; 1.60] | Ertugliflozin_  high_dosage | . | 0.96 [0.71; 1.30] | . |
| ***0.60 [0.38; 0.93]** | 0.57 [0.26; 1.22] | 0.68 [0.45; 1.03] | 0.54 [0.15; 2.02] | 0.72 [0.35; 1.47] | 0.84 [0.58; 1.20] | 0.85 [0.58; 1.23] | 0.87 [0.58; 1.29] | 0.87 [0.25; 3.01] | 0.90 [0.67; 1.22] | 0.91 [0.59; 1.39] | 0.95 [0.64; 1.40] | 0.99 [0.63; 1.56] | 1.00 [0.68; 1.48] | Exenatide | 0.96 [0.74; 1.24] | . |
| ***0.57 [0.40; 0.83]** | 0.55 [0.27; 1.12] | ***0.65 [0.47; 0.91]** | 0.52 [0.14; 1.90] | 0.69 [0.36; 1.35] | 0.80 [0.62; 1.04] | 0.81 [0.62; 1.07] | 0.83 [0.61; 1.13] | 0.83 [0.25; 2.81] | 0.87 [0.74; 1.02] | 0.87 [0.62; 1.23] | 0.91 [0.67; 1.23] | 0.95 [0.66; 1.38] | 0.96 [0.71; 1.30] | 0.96 [0.74; 1.24] | Placebo_  or_Control | 0.92 [0.63; 1.35] |
| ***0.53 [0.31; 0.90]** | 0.50 [0.22; 1.14] | ***0.60 [0.37; 0.99]** | 0.48 [0.13; 1.85] | 0.64 [0.30; 1.38] | 0.74 [0.47; 1.17] | 0.75 [0.47; 1.20] | 0.77 [0.47; 1.25] | 0.77 [0.22; 2.75] | 0.80 [0.53; 1.21] | 0.80 [0.48; 1.34] | 0.84 [0.52; 1.36] | 0.88 [0.52; 1.50] | 0.89 [0.55; 1.44] | 0.89 [0.56; 1.40] | 0.92 [0.63; 1.35] | Dulaglutide |

Data present as OR [95%CIs]. Pairwise (upper-right portion) and network (lower-left portion) meta-analysis results are presented as estimate effect sizes for the outcome of safety profile (drop-out rate). Interventions are reported in order of mean ranking of safety, and outcomes are expressed as odds ratio (OR) (95% confidence intervals) (95%CIs). For the pairwise meta-analyses, OR of less than 1 indicate that the treatment specified in the row got more safety than that specified in the column. For the network meta-analysis (NMA), OR of less than 1 indicate that the treatment specified in the column got more safety than that specified in the row. Bold results marked with * indicate statistical significance.

*Dosage definition: Canagliflozin (Low: 100mg, and High: 300mg); Ertugliflozin (Low: 5mg, and High: 15mg); Injectable semaglutide (Low: 0.5mg, and High: 1.0mg); Empagliflozin (Low: 1-10mg, and High: 25-50mg).*

*Abbreviation: 95%CIs: 95% confidence intervals; GLP-1 agonist: glucagon-like peptide-1 agonist; NMA: network meta-analysis; OR: odds ratio; RCT: randomized controlled trial; SGLT2 inhibitor: sodium–glucose cotransporter 2 inhibitor*

**Tab. S6: SUCRA (Surface under the cumulative ranking) of primary outcome: overall events of neurodegenerative diseases**

| Treatment | Rank 1 | Rank 2 | Rank 3 | Rank 4 | Rank 5 | Rank 6 | Rank 7 | Rank 8 | Rank 9 | Rank 10 | Rank 11 | Rank 12 | Rank 13 | Rank 14 | Rank 15 | Rank 16 | Rank 17 | SUCRA |
| --- | --- | --- | --- | --- | --- | --- | --- | --- | --- | --- | --- | --- | --- | --- | --- | --- | --- | --- |
| Albiglutide | 0 | 0 | 0.0004375 | 0.0045625 | 0.00605 | 0.0038875 | 0.004475 | 0.0045625 | 0.0063875 | 0.010525 | 0.0149375 | 0.0202375 | 0.1390125 | 0.030475 | 0.2195625 | 0.028725 | 0.5061625 | 10.60953125 |
| Canagliflozin_high_dosage | 0 | 0 | 0.000175 | 0.0004625 | 0.001 | 0.0010625 | 0.0014 | 0.0019125 | 0.0032875 | 0.0075 | 0.0099875 | 0.0191375 | 0.09615 | 0.2106375 | 0.16035 | 0.28105 | 0.2058875 | 11.97523438 |
| Canagliflozin_low_dosage | 0 | 0 | 0.0001375 | 0.0016125 | 0.001925 | 0.0021125 | 0.002375 | 0.0031375 | 0.005525 | 0.0115375 | 0.0202125 | 0.0358 | 0.192625 | 0.1580375 | 0.2125 | 0.2650375 | 0.087425 | 15.506875 |
| Dapagliflozin | 1.25E-05 | 0.0013625 | 0.0213125 | 0.1677625 | 0.279075 | 0.229325 | 0.145625 | 0.0821875 | 0.04475 | 0.0194375 | 0.007275 | 0.001525 | 0.000325 | 2.50E-05 | 0 | 0 | 0 | 69.4628125 |
| Dulaglutide | 0.00015 | 0.00575 | 0.0572875 | 0.3624125 | 0.2307875 | 0.1340125 | 0.08345 | 0.0529125 | 0.0379625 | 0.0209125 | 0.010625 | 0.00285 | 0.000725 | 0.0001375 | 2.50E-05 | 0 | 0 | 73.04867188 |
| Empagliflozin_high_dosage | 0.2667 | 0.30595 | 0.335975 | 0.03205 | 0.0155125 | 0.0103875 | 0.0076875 | 0.0073625 | 0.0069375 | 0.0052625 | 0.0036 | 0.002025 | 0.000425 | 1.00E-04 | 2.50E-05 | 0 | 0 | 90.91507813 |
| Empagliflozin_low_dosage | 0 | 7.50E-05 | 0.002625 | 0.03785 | 0.0905 | 0.1462375 | 0.157625 | 0.1725 | 0.1723 | 0.1240375 | 0.0708125 | 0.0198875 | 0.004275 | 0.0010875 | 0.0001625 | 2.50E-05 | 0 | 57.15609375 |
| Ertugliflozin_high_dosage | 0 | 3.75E-05 | 0.0011125 | 0.0089 | 0.0082125 | 0.0079875 | 0.0073375 | 0.0074625 | 0.011 | 0.020325 | 0.0302375 | 0.0499125 | 0.218225 | 0.1832625 | 0.1995875 | 0.1924375 | 0.0539625 | 19.5896875 |
| Ertugliflozin_low_dosage | 0 | 0 | 0.0002375 | 0.002225 | 0.0030125 | 0.003075 | 0.0052625 | 0.0046875 | 0.0061125 | 0.011 | 0.016825 | 0.029325 | 0.112375 | 0.26 | 0.17765 | 0.222675 | 0.1455375 | 14.86242188 |
| Exenatide | 0 | 2.50E-05 | 0.00015 | 0.0013375 | 0.0038 | 0.0072875 | 0.011275 | 0.02105 | 0.0508125 | 0.133325 | 0.1869625 | 0.39 | 0.0904875 | 0.082925 | 0.0145625 | 0.005675 | 0.000325 | 34.405625 |
| Inject_semaglutide_low_dosage | 0 | 0.0001875 | 0.0026125 | 0.0162875 | 0.0270125 | 0.0353 | 0.0412 | 0.04905 | 0.0810875 | 0.1590375 | 0.252025 | 0.2165125 | 0.0701125 | 0.039675 | 0.006975 | 0.002525 | 4.00E-04 | 41.1853125 |
| Inject_semaglutide_high_dosage | 7.50E-05 | 0.0018625 | 0.0174 | 0.087175 | 0.070875 | 0.0738 | 0.068325 | 0.0713125 | 0.1104625 | 0.173475 | 0.1665875 | 0.099375 | 0.0386 | 0.0165125 | 0.0029125 | 0.0011375 | 0.0001125 | 51.241875 |
| Liraglutide | 0 | 0.00105 | 0.01185 | 0.0844 | 0.1149875 | 0.130175 | 0.1222625 | 0.1339125 | 0.1524125 | 0.1208 | 0.0818 | 0.033625 | 0.0085375 | 0.003625 | 0.0004125 | 0.000125 | 2.50E-05 | 58.05164063 |
| Lixisenatide | 0.000325 | 0.0047125 | 0.034725 | 0.1594 | 0.107875 | 0.087225 | 0.0721375 | 0.076475 | 0.112 | 0.11085 | 0.109925 | 0.077175 | 0.0276875 | 0.0134625 | 0.005275 | 0.0005875 | 0.0001625 | 57.35945313 |
| Oral_semaglutide | 0.217925 | 0.385075 | 0.3428375 | 0.0204625 | 0.010125 | 0.0072375 | 0.0040125 | 0.003475 | 0.0032 | 0.003025 | 0.0016625 | 0.000725 | 2.00E-04 | 3.75E-05 | 0 | 0 | 0 | 91.64039063 |
| Placebo_or_Control | 0 | 0 | 3.75E-05 | 0.0022875 | 0.0249875 | 0.1186125 | 0.2646375 | 0.3073375 | 0.1952625 | 0.0686 | 0.0162 | 0.0018125 | 0.000225 | 0 | 0 | 0 | 0 | 57.47953125 |
| Sotagliflozin | 0.5148125 | 0.2939125 | 0.1710875 | 0.0108125 | 0.0042625 | 0.002275 | 0.0009125 | 0.0006625 | 5.00E-04 | 0.00035 | 0.000325 | 7.50E-05 | 1.25E-05 | 0 | 0 | 0 | 0 | 95.50976563 |

*Dosage definition: Canagliflozin (Low: 100mg, and High: 300mg); Ertugliflozin (Low: 5mg, and High: 15mg); Injectable semaglutide (Low: 0.5mg, and High: 1.0mg); Empagliflozin (Low: 1-10mg, and High: 25-50mg).*

**Tab. S7A: inconsistency within the primary outcome: overall events of neurodegenerative diseases**

| Comparison | No.Studies | NMA | Direct | Indirect | Difference | Diff_95CI_lower | Diff_95CI_upper | *p* value |
| --- | --- | --- | --- | --- | --- | --- | --- | --- |
| Albiglutide:Canagliflozin_high_dosage | 0 | -0.511909873 | NA | -0.511909873 | NA | NA | NA | NA |
| Albiglutide:Canagliflozin_low_dosage | 0 | 0.000935138 | NA | 0.000935138 | NA | NA | NA | NA |
| Albiglutide:Dapagliflozin | 0 | 1.455756407 | NA | 1.455756407 | NA | NA | NA | NA |
| Albiglutide:Dulaglutide | 0 | 1.963672542 | NA | 1.963672542 | NA | NA | NA | NA |
| Albiglutide:Empagliflozin_high_dosage | 0 | 2.217614124 | NA | 2.217614124 | NA | NA | NA | NA |
| Albiglutide:Empagliflozin_low_dosage | 0 | 1.136178715 | NA | 1.136178715 | NA | NA | NA | NA |
| Albiglutide:Ertugliflozin_high_dosage | 0 | 0.000152122 | NA | 0.000152122 | NA | NA | NA | NA |
| Albiglutide:Ertugliflozin_low_dosage | 0 | -0.511401967 | NA | -0.511401967 | NA | NA | NA | NA |
| Albiglutide:Exenatide | 0 | -0.469492927 | NA | -0.469492927 | NA | NA | NA | NA |
| Albiglutide:Inject_semaglutide_low_dosage | 0 | -0.287894117 | NA | -0.287894117 | NA | NA | NA | NA |
| Albiglutide:Inject_semaglutide_high_dosage | 0 | 0.401605644 | NA | 0.401605644 | NA | NA | NA | NA |
| Albiglutide:Liraglutide | 0 | 1.097542979 | NA | 1.097542979 | NA | NA | NA | NA |
| Albiglutide:Lixisenatide | 0 | 1.098070266 | NA | 1.098070266 | NA | NA | NA | NA |
| Albiglutide:Oral_semaglutide | 0 | 2.709095623 | NA | 2.709095623 | NA | NA | NA | NA |
| Albiglutide:Placebo_or_Control | 1 | 1.098400245 | 1.098400245 | NA | NA | NA | NA | NA |
| Albiglutide:Sotagliflozin | 0 | 2.650496293 | NA | 2.650496293 | NA | NA | NA | NA |
| Canagliflozin_high_dosage:Canagliflozin_low_dosage | 1 | 0.512845011 | 0.512904627 | 0.512141808 | 0.000762818 | -7.244831831 | 7.246357468 | 0.99983536 |
| Canagliflozin_high_dosage:Dapagliflozin | 0 | 1.967666281 | NA | 1.967666281 | NA | NA | NA | NA |
| Canagliflozin_high_dosage:Dulaglutide | 0 | 2.475582415 | NA | 2.475582415 | NA | NA | NA | NA |
| Canagliflozin_high_dosage:Empagliflozin_high_dosage | 0 | 2.729523997 | NA | 2.729523997 | NA | NA | NA | NA |
| Canagliflozin_high_dosage:Empagliflozin_low_dosage | 0 | 1.648088588 | NA | 1.648088588 | NA | NA | NA | NA |
| Canagliflozin_high_dosage:Ertugliflozin_high_dosage | 0 | 0.512061995 | NA | 0.512061995 | NA | NA | NA | NA |
| Canagliflozin_high_dosage:Ertugliflozin_low_dosage | 0 | 0.000507907 | NA | 0.000507907 | NA | NA | NA | NA |
| Canagliflozin_high_dosage:Exenatide | 0 | 0.042416946 | NA | 0.042416946 | NA | NA | NA | NA |
| Canagliflozin_high_dosage:Inject_semaglutide_low_dosage | 0 | 0.224015757 | NA | 0.224015757 | NA | NA | NA | NA |
| Canagliflozin_high_dosage:Inject_semaglutide_high_dosage | 0 | 0.913515517 | NA | 0.913515517 | NA | NA | NA | NA |
| Canagliflozin_high_dosage:Liraglutide | 0 | 1.609452852 | NA | 1.609452852 | NA | NA | NA | NA |
| Canagliflozin_high_dosage:Lixisenatide | 0 | 1.609980139 | NA | 1.609980139 | NA | NA | NA | NA |
| Canagliflozin_high_dosage:Oral_semaglutide | 0 | 3.221005497 | NA | 3.221005497 | NA | NA | NA | NA |
| Canagliflozin_high_dosage:Placebo_or_Control | 1 | 1.610310118 | 1.610131394 | 1.610703491 | -0.000572097 | -5.434607899 | 5.433463706 | 0.99983536 |
| Canagliflozin_high_dosage:Sotagliflozin | 0 | 3.162406166 | NA | 3.162406166 | NA | NA | NA | NA |
| Canagliflozin_low_dosage:Dapagliflozin | 0 | 1.45482127 | NA | 1.45482127 | NA | NA | NA | NA |
| Canagliflozin_low_dosage:Dulaglutide | 0 | 1.962737404 | NA | 1.962737404 | NA | NA | NA | NA |
| Canagliflozin_low_dosage:Empagliflozin_high_dosage | 0 | 2.216678986 | NA | 2.216678986 | NA | NA | NA | NA |
| Canagliflozin_low_dosage:Empagliflozin_low_dosage | 0 | 1.135243578 | NA | 1.135243578 | NA | NA | NA | NA |
| Canagliflozin_low_dosage:Ertugliflozin_high_dosage | 0 | -0.000783016 | NA | -0.000783016 | NA | NA | NA | NA |
| Canagliflozin_low_dosage:Ertugliflozin_low_dosage | 0 | -0.512337104 | NA | -0.512337104 | NA | NA | NA | NA |
| Canagliflozin_low_dosage:Exenatide | 0 | -0.470428065 | NA | -0.470428065 | NA | NA | NA | NA |
| Canagliflozin_low_dosage:Inject_semaglutide_low_dosage | 0 | -0.288829254 | NA | -0.288829254 | NA | NA | NA | NA |
| Canagliflozin_low_dosage:Inject_semaglutide_high_dosage | 0 | 0.400670506 | NA | 0.400670506 | NA | NA | NA | NA |
| Canagliflozin_low_dosage:Liraglutide | 0 | 1.096607842 | NA | 1.096607842 | NA | NA | NA | NA |
| Canagliflozin_low_dosage:Lixisenatide | 0 | 1.097135128 | NA | 1.097135128 | NA | NA | NA | NA |
| Canagliflozin_low_dosage:Oral_semaglutide | 0 | 2.708160486 | NA | 2.708160486 | NA | NA | NA | NA |
| Canagliflozin_low_dosage:Placebo_or_Control | 2 | 1.097465107 | 1.097465107 | NA | NA | NA | NA | NA |
| Canagliflozin_low_dosage:Sotagliflozin | 0 | 2.649561156 | NA | 2.649561156 | NA | NA | NA | NA |
| Dapagliflozin:Dulaglutide | 0 | 0.507916134 | NA | 0.507916134 | NA | NA | NA | NA |
| Dapagliflozin:Empagliflozin_high_dosage | 0 | 0.761857717 | NA | 0.761857717 | NA | NA | NA | NA |
| Dapagliflozin:Empagliflozin_low_dosage | 0 | -0.319577692 | NA | -0.319577692 | NA | NA | NA | NA |
| Dapagliflozin:Ertugliflozin_high_dosage | 0 | -1.455604285 | NA | -1.455604285 | NA | NA | NA | NA |
| Dapagliflozin:Ertugliflozin_low_dosage | 0 | -1.967158374 | NA | -1.967158374 | NA | NA | NA | NA |
| Dapagliflozin:Exenatide | 0 | -1.925249335 | NA | -1.925249335 | NA | NA | NA | NA |
| Dapagliflozin:Inject_semaglutide_low_dosage | 0 | -1.743650524 | NA | -1.743650524 | NA | NA | NA | NA |
| Dapagliflozin:Inject_semaglutide_high_dosage | 0 | -1.054150764 | NA | -1.054150764 | NA | NA | NA | NA |
| Dapagliflozin:Liraglutide | 0 | -0.358213428 | NA | -0.358213428 | NA | NA | NA | NA |
| Dapagliflozin:Lixisenatide | 0 | -0.357686141 | NA | -0.357686141 | NA | NA | NA | NA |
| Dapagliflozin:Oral_semaglutide | 0 | 1.253339216 | NA | 1.253339216 | NA | NA | NA | NA |
| Dapagliflozin:Placebo_or_Control | 4 | -0.357356163 | -0.357356163 | NA | NA | NA | NA | NA |
| Dapagliflozin:Sotagliflozin | 0 | 1.194739886 | NA | 1.194739886 | NA | NA | NA | NA |
| Dulaglutide:Empagliflozin_high_dosage | 0 | 0.253941582 | NA | 0.253941582 | NA | NA | NA | NA |
| Dulaglutide:Empagliflozin_low_dosage | 0 | -0.827493827 | NA | -0.827493827 | NA | NA | NA | NA |
| Dulaglutide:Ertugliflozin_high_dosage | 0 | -1.96352042 | NA | -1.96352042 | NA | NA | NA | NA |
| Dulaglutide:Ertugliflozin_low_dosage | 0 | -2.475074508 | NA | -2.475074508 | NA | NA | NA | NA |
| Dulaglutide:Exenatide | 0 | -2.433165469 | NA | -2.433165469 | NA | NA | NA | NA |
| Dulaglutide:Inject_semaglutide_low_dosage | 0 | -2.251566658 | NA | -2.251566658 | NA | NA | NA | NA |
| Dulaglutide:Inject_semaglutide_high_dosage | 0 | -1.562066898 | NA | -1.562066898 | NA | NA | NA | NA |
| Dulaglutide:Liraglutide | 0 | -0.866129562 | NA | -0.866129562 | NA | NA | NA | NA |
| Dulaglutide:Lixisenatide | 0 | -0.865602276 | NA | -0.865602276 | NA | NA | NA | NA |
| Dulaglutide:Oral_semaglutide | 0 | 0.745423082 | NA | 0.745423082 | NA | NA | NA | NA |
| Dulaglutide:Placebo_or_Control | 2 | -0.865272297 | -0.865272297 | NA | NA | NA | NA | NA |
| Dulaglutide:Sotagliflozin | 0 | 0.686823752 | NA | 0.686823752 | NA | NA | NA | NA |
| Empagliflozin_high_dosage:Empagliflozin_low_dosage | 1 | -1.081435409 | -1.097758864 | -0.920211765 | -0.177547099 | -10.73484267 | 10.37974848 | 0.973705167 |
| Empagliflozin_high_dosage:Ertugliflozin_high_dosage | 0 | -2.217462002 | NA | -2.217462002 | NA | NA | NA | NA |
| Empagliflozin_high_dosage:Ertugliflozin_low_dosage | 0 | -2.72901609 | NA | -2.72901609 | NA | NA | NA | NA |
| Empagliflozin_high_dosage:Exenatide | 0 | -2.687107051 | NA | -2.687107051 | NA | NA | NA | NA |
| Empagliflozin_high_dosage:Inject_semaglutide_low_dosage | 0 | -2.50550824 | NA | -2.50550824 | NA | NA | NA | NA |
| Empagliflozin_high_dosage:Inject_semaglutide_high_dosage | 0 | -1.81600848 | NA | -1.81600848 | NA | NA | NA | NA |
| Empagliflozin_high_dosage:Liraglutide | 0 | -1.120071145 | NA | -1.120071145 | NA | NA | NA | NA |
| Empagliflozin_high_dosage:Lixisenatide | 0 | -1.119543858 | NA | -1.119543858 | NA | NA | NA | NA |
| Empagliflozin_high_dosage:Oral_semaglutide | 0 | 0.4914815 | NA | 0.4914815 | NA | NA | NA | NA |
| Empagliflozin_high_dosage:Placebo_or_Control | 1 | -1.119213879 | -1.10289037 | -1.280437031 | 0.177546661 | -10.37972287 | 10.73481619 | 0.973705167 |
| Empagliflozin_high_dosage:Sotagliflozin | 0 | 0.432882169 | NA | 0.432882169 | NA | NA | NA | NA |
| Empagliflozin_low_dosage:Ertugliflozin_high_dosage | 0 | -1.136026593 | NA | -1.136026593 | NA | NA | NA | NA |
| Empagliflozin_low_dosage:Ertugliflozin_low_dosage | 0 | -1.647580682 | NA | -1.647580682 | NA | NA | NA | NA |
| Empagliflozin_low_dosage:Exenatide | 0 | -1.605671642 | NA | -1.605671642 | NA | NA | NA | NA |
| Empagliflozin_low_dosage:Inject_semaglutide_low_dosage | 0 | -1.424072832 | NA | -1.424072832 | NA | NA | NA | NA |
| Empagliflozin_low_dosage:Inject_semaglutide_high_dosage | 0 | -0.734573072 | NA | -0.734573072 | NA | NA | NA | NA |
| Empagliflozin_low_dosage:Liraglutide | 0 | -0.038635736 | NA | -0.038635736 | NA | NA | NA | NA |
| Empagliflozin_low_dosage:Lixisenatide | 0 | -0.038108449 | NA | -0.038108449 | NA | NA | NA | NA |
| Empagliflozin_low_dosage:Oral_semaglutide | 0 | 1.572916908 | NA | 1.572916908 | NA | NA | NA | NA |
| Empagliflozin_low_dosage:Placebo_or_Control | 3 | -0.037778471 | -0.037778471 | NA | NA | NA | NA | NA |
| Empagliflozin_low_dosage:Sotagliflozin | 0 | 1.514317578 | NA | 1.514317578 | NA | NA | NA | NA |
| Ertugliflozin_high_dosage:Ertugliflozin_low_dosage | 1 | -0.511554089 | -0.511554089 | NA | NA | NA | NA | NA |
| Ertugliflozin_high_dosage:Exenatide | 0 | -0.469645049 | NA | -0.469645049 | NA | NA | NA | NA |
| Ertugliflozin_high_dosage:Inject_semaglutide_low_dosage | 0 | -0.288046239 | NA | -0.288046239 | NA | NA | NA | NA |
| Ertugliflozin_high_dosage:Inject_semaglutide_high_dosage | 0 | 0.401453522 | NA | 0.401453522 | NA | NA | NA | NA |
| Ertugliflozin_high_dosage:Liraglutide | 0 | 1.097390857 | NA | 1.097390857 | NA | NA | NA | NA |
| Ertugliflozin_high_dosage:Lixisenatide | 0 | 1.097918144 | NA | 1.097918144 | NA | NA | NA | NA |
| Ertugliflozin_high_dosage:Oral_semaglutide | 0 | 2.708943501 | NA | 2.708943501 | NA | NA | NA | NA |
| Ertugliflozin_high_dosage:Placebo_or_Control | 1 | 1.098248123 | 1.098248123 | NA | NA | NA | NA | NA |
| Ertugliflozin_high_dosage:Sotagliflozin | 0 | 2.650344171 | NA | 2.650344171 | NA | NA | NA | NA |
| Ertugliflozin_low_dosage:Exenatide | 0 | 0.041909039 | NA | 0.041909039 | NA | NA | NA | NA |
| Ertugliflozin_low_dosage:Inject_semaglutide_low_dosage | 0 | 0.22350785 | NA | 0.22350785 | NA | NA | NA | NA |
| Ertugliflozin_low_dosage:Inject_semaglutide_high_dosage | 0 | 0.91300761 | NA | 0.91300761 | NA | NA | NA | NA |
| Ertugliflozin_low_dosage:Liraglutide | 0 | 1.608944946 | NA | 1.608944946 | NA | NA | NA | NA |
| Ertugliflozin_low_dosage:Lixisenatide | 0 | 1.609472233 | NA | 1.609472233 | NA | NA | NA | NA |
| Ertugliflozin_low_dosage:Oral_semaglutide | 0 | 3.22049759 | NA | 3.22049759 | NA | NA | NA | NA |
| Ertugliflozin_low_dosage:Placebo_or_Control | 1 | 1.609802211 | 1.609802211 | NA | NA | NA | NA | NA |
| Ertugliflozin_low_dosage:Sotagliflozin | 0 | 3.16189826 | NA | 3.16189826 | NA | NA | NA | NA |
| Exenatide:Inject_semaglutide_low_dosage | 0 | 0.181598811 | NA | 0.181598811 | NA | NA | NA | NA |
| Exenatide:Inject_semaglutide_high_dosage | 0 | 0.871098571 | NA | 0.871098571 | NA | NA | NA | NA |
| Exenatide:Liraglutide | 0 | 1.567035906 | NA | 1.567035906 | NA | NA | NA | NA |
| Exenatide:Lixisenatide | 0 | 1.567563193 | NA | 1.567563193 | NA | NA | NA | NA |
| Exenatide:Oral_semaglutide | 0 | 3.178588551 | NA | 3.178588551 | NA | NA | NA | NA |
| Exenatide:Placebo_or_Control | 2 | 1.567893172 | 1.567893172 | NA | NA | NA | NA | NA |
| Exenatide:Sotagliflozin | 0 | 3.11998922 | NA | 3.11998922 | NA | NA | NA | NA |
| Inject_semaglutide_low_dosage:Inject_semaglutide_high_dosage | 1 | 0.68949976 | 0.68949976 | NA | NA | NA | NA | NA |
| Inject_semaglutide_low_dosage:Liraglutide | 0 | 1.385437096 | NA | 1.385437096 | NA | NA | NA | NA |
| Inject_semaglutide_low_dosage:Lixisenatide | 0 | 1.385964383 | NA | 1.385964383 | NA | NA | NA | NA |
| Inject_semaglutide_low_dosage:Oral_semaglutide | 0 | 2.99698974 | NA | 2.99698974 | NA | NA | NA | NA |
| Inject_semaglutide_low_dosage:Placebo_or_Control | 1 | 1.386294361 | 1.386294361 | NA | NA | NA | NA | NA |
| Inject_semaglutide_low_dosage:Sotagliflozin | 0 | 2.93839041 | NA | 2.93839041 | NA | NA | NA | NA |
| Inject_semaglutide_high_dosage:Liraglutide | 0 | 0.695937336 | NA | 0.695937336 | NA | NA | NA | NA |
| Inject_semaglutide_high_dosage:Lixisenatide | 0 | 0.696464622 | NA | 0.696464622 | NA | NA | NA | NA |
| Inject_semaglutide_high_dosage:Oral_semaglutide | 0 | 2.30748998 | NA | 2.30748998 | NA | NA | NA | NA |
| Inject_semaglutide_high_dosage:Placebo_or_Control | 1 | 0.696794601 | 0.696794601 | NA | NA | NA | NA | NA |
| Inject_semaglutide_high_dosage:Sotagliflozin | 0 | 2.24889065 | NA | 2.24889065 | NA | NA | NA | NA |
| Liraglutide:Lixisenatide | 0 | 0.000527287 | NA | 0.000527287 | NA | NA | NA | NA |
| Liraglutide:Oral_semaglutide | 0 | 1.611552644 | NA | 1.611552644 | NA | NA | NA | NA |
| Liraglutide:Placebo_or_Control | 1 | 0.000857265 | 0.000857265 | NA | NA | NA | NA | NA |
| Liraglutide:Sotagliflozin | 0 | 1.552953314 | NA | 1.552953314 | NA | NA | NA | NA |
| Lixisenatide:Oral_semaglutide | 0 | 1.611025357 | NA | 1.611025357 | NA | NA | NA | NA |
| Lixisenatide:Placebo_or_Control | 1 | 0.000329979 | 0.000329979 | NA | NA | NA | NA | NA |
| Lixisenatide:Sotagliflozin | 0 | 1.552426027 | NA | 1.552426027 | NA | NA | NA | NA |
| Oral_semaglutide:Placebo_or_Control | 1 | -1.610695379 | -1.610695379 | NA | NA | NA | NA | NA |
| Oral_semaglutide:Sotagliflozin | 0 | -0.05859933 | NA | -0.05859933 | NA | NA | NA | NA |
| Sotagliflozin:Placebo_or_Control | 2 | -1.552096049 | -1.552096049 | NA | NA | NA | NA | NA |

**Tab. S7B: inconsistency within the primary outcome: subgroup of Parkinson’s disease events**

| Comparison | No.Studies | NMA | Direct | Indirect | Difference | Diff_95CI_lower | Diff_95CI_upper | *p* value |
| --- | --- | --- | --- | --- | --- | --- | --- | --- |
| Canagliflozin_high_dosage:Canagliflozin_low_dosage | 1 | 0.001325906 | 0.001385522 | 0.000432247 | 0.000953275 | -9.053686138 | 9.055592688 | 0.99983536 |
| Canagliflozin_high_dosage:Dapagliflozin | 0 | 2.361359069 | NA | 2.361359069 | NA | NA | NA | NA |
| Canagliflozin_high_dosage:Dulaglutide | 0 | 1.791128968 | NA | 1.791128968 | NA | NA | NA | NA |
| Canagliflozin_high_dosage:Empagliflozin | 0 | 1.636425402 | NA | 1.636425402 | NA | NA | NA | NA |
| Canagliflozin_high_dosage:Exenatide | 0 | -0.245585632 | NA | -0.245585632 | NA | NA | NA | NA |
| Canagliflozin_high_dosage:Inject_semaglutide_low_dosage | 0 | -1.204097248 | NA | -1.204097248 | NA | NA | NA | NA |
| Canagliflozin_high_dosage:Inject_semaglutide_high_dosage | 0 | -0.696916829 | NA | -0.696916829 | NA | NA | NA | NA |
| Canagliflozin_high_dosage:Liraglutide | 0 | 1.503613378 | NA | 1.503613378 | NA | NA | NA | NA |
| Canagliflozin_high_dosage:Oral_semaglutide | 0 | 2.198031837 | NA | 2.198031837 | NA | NA | NA | NA |
| Canagliflozin_high_dosage:Placebo_or_Control | 1 | 1.098791013 | 1.098612289 | 1.099247915 | -0.000635626 | -6.038103372 | 6.036832119 | 0.99983536 |
| Canagliflozin_high_dosage:Sotagliflozin | 0 | 2.650887061 | NA | 2.650887061 | NA | NA | NA | NA |
| Canagliflozin_low_dosage:Dapagliflozin | 0 | 2.360033163 | NA | 2.360033163 | NA | NA | NA | NA |
| Canagliflozin_low_dosage:Dulaglutide | 0 | 1.789803062 | NA | 1.789803062 | NA | NA | NA | NA |
| Canagliflozin_low_dosage:Empagliflozin | 0 | 1.635099496 | NA | 1.635099496 | NA | NA | NA | NA |
| Canagliflozin_low_dosage:Exenatide | 0 | -0.246911538 | NA | -0.246911538 | NA | NA | NA | NA |
| Canagliflozin_low_dosage:Inject_semaglutide_low_dosage | 0 | -1.205423154 | NA | -1.205423154 | NA | NA | NA | NA |
| Canagliflozin_low_dosage:Inject_semaglutide_high_dosage | 0 | -0.698242735 | NA | -0.698242735 | NA | NA | NA | NA |
| Canagliflozin_low_dosage:Liraglutide | 0 | 1.502287473 | NA | 1.502287473 | NA | NA | NA | NA |
| Canagliflozin_low_dosage:Oral_semaglutide | 0 | 2.196705931 | NA | 2.196705931 | NA | NA | NA | NA |
| Canagliflozin_low_dosage:Placebo_or_Control | 2 | 1.097465107 | 1.097465107 | NA | NA | NA | NA | NA |
| Canagliflozin_low_dosage:Sotagliflozin | 0 | 2.649561156 | NA | 2.649561156 | NA | NA | NA | NA |
| Dapagliflozin:Dulaglutide | 0 | -0.570230101 | NA | -0.570230101 | NA | NA | NA | NA |
| Dapagliflozin:Empagliflozin | 0 | -0.724933667 | NA | -0.724933667 | NA | NA | NA | NA |
| Dapagliflozin:Exenatide | 0 | -2.606944701 | NA | -2.606944701 | NA | NA | NA | NA |
| Dapagliflozin:Inject_semaglutide_low_dosage | 0 | -3.565456317 | NA | -3.565456317 | NA | NA | NA | NA |
| Dapagliflozin:Inject_semaglutide_high_dosage | 0 | -3.058275898 | NA | -3.058275898 | NA | NA | NA | NA |
| Dapagliflozin:Liraglutide | 0 | -0.85774569 | NA | -0.85774569 | NA | NA | NA | NA |
| Dapagliflozin:Oral_semaglutide | 0 | -0.163327232 | NA | -0.163327232 | NA | NA | NA | NA |
| Dapagliflozin:Placebo_or_Control | 3 | -1.262568056 | -1.262568056 | NA | NA | NA | NA | NA |
| Dapagliflozin:Sotagliflozin | 0 | 0.289527993 | NA | 0.289527993 | NA | NA | NA | NA |
| Dulaglutide:Empagliflozin | 0 | -0.154703566 | NA | -0.154703566 | NA | NA | NA | NA |
| Dulaglutide:Exenatide | 0 | -2.0367146 | NA | -2.0367146 | NA | NA | NA | NA |
| Dulaglutide:Inject_semaglutide_low_dosage | 0 | -2.995226216 | NA | -2.995226216 | NA | NA | NA | NA |
| Dulaglutide:Inject_semaglutide_high_dosage | 0 | -2.488045797 | NA | -2.488045797 | NA | NA | NA | NA |
| Dulaglutide:Liraglutide | 0 | -0.28751559 | NA | -0.28751559 | NA | NA | NA | NA |
| Dulaglutide:Oral_semaglutide | 0 | 0.406902869 | NA | 0.406902869 | NA | NA | NA | NA |
| Dulaglutide:Placebo_or_Control | 1 | -0.692337955 | -0.692337955 | NA | NA | NA | NA | NA |
| Dulaglutide:Sotagliflozin | 0 | 0.859758093 | NA | 0.859758093 | NA | NA | NA | NA |
| Empagliflozin:Exenatide | 0 | -1.882011034 | NA | -1.882011034 | NA | NA | NA | NA |
| Empagliflozin:Inject_semaglutide_low_dosage | 0 | -2.84052265 | NA | -2.84052265 | NA | NA | NA | NA |
| Empagliflozin:Inject_semaglutide_high_dosage | 0 | -2.333342231 | NA | -2.333342231 | NA | NA | NA | NA |
| Empagliflozin:Liraglutide | 0 | -0.132812023 | NA | -0.132812023 | NA | NA | NA | NA |
| Empagliflozin:Oral_semaglutide | 0 | 0.561606435 | NA | 0.561606435 | NA | NA | NA | NA |
| Empagliflozin:Placebo_or_Control | 3 | -0.537634389 | -0.537634389 | NA | NA | NA | NA | NA |
| Empagliflozin:Sotagliflozin | 0 | 1.01446166 | NA | 1.01446166 | NA | NA | NA | NA |
| Exenatide:Inject_semaglutide_low_dosage | 0 | -0.958511616 | NA | -0.958511616 | NA | NA | NA | NA |
| Exenatide:Inject_semaglutide_high_dosage | 0 | -0.451331197 | NA | -0.451331197 | NA | NA | NA | NA |
| Exenatide:Liraglutide | 0 | 1.749199011 | NA | 1.749199011 | NA | NA | NA | NA |
| Exenatide:Oral_semaglutide | 0 | 2.443617469 | NA | 2.443617469 | NA | NA | NA | NA |
| Exenatide:Placebo_or_Control | 2 | 1.344376645 | 1.344376645 | NA | NA | NA | NA | NA |
| Exenatide:Sotagliflozin | 0 | 2.896472694 | NA | 2.896472694 | NA | NA | NA | NA |
| Inject_semaglutide_low_dosage:Inject_semaglutide_high_dosage | 1 | 0.507180419 | 0.507180419 | NA | NA | NA | NA | NA |
| Inject_semaglutide_low_dosage:Liraglutide | 0 | 2.707710627 | NA | 2.707710627 | NA | NA | NA | NA |
| Inject_semaglutide_low_dosage:Oral_semaglutide | 0 | 3.402129085 | NA | 3.402129085 | NA | NA | NA | NA |
| Inject_semaglutide_low_dosage:Placebo_or_Control | 1 | 2.302888261 | 2.302888261 | NA | NA | NA | NA | NA |
| Inject_semaglutide_low_dosage:Sotagliflozin | 0 | 3.85498431 | NA | 3.85498431 | NA | NA | NA | NA |
| Inject_semaglutide_high_dosage:Liraglutide | 0 | 2.200530208 | NA | 2.200530208 | NA | NA | NA | NA |
| Inject_semaglutide_high_dosage:Oral_semaglutide | 0 | 2.894948666 | NA | 2.894948666 | NA | NA | NA | NA |
| Inject_semaglutide_high_dosage:Placebo_or_Control | 1 | 1.795707842 | 1.795707842 | NA | NA | NA | NA | NA |
| Inject_semaglutide_high_dosage:Sotagliflozin | 0 | 3.347803891 | NA | 3.347803891 | NA | NA | NA | NA |
| Liraglutide:Oral_semaglutide | 0 | 0.694418458 | NA | 0.694418458 | NA | NA | NA | NA |
| Liraglutide:Placebo_or_Control | 1 | -0.404822366 | -0.404822366 | NA | NA | NA | NA | NA |
| Liraglutide:Sotagliflozin | 0 | 1.147273683 | NA | 1.147273683 | NA | NA | NA | NA |
| Oral_semaglutide:Placebo_or_Control | 1 | -1.099240824 | -1.099240824 | NA | NA | NA | NA | NA |
| Oral_semaglutide:Sotagliflozin | 0 | 0.452855224 | NA | 0.452855224 | NA | NA | NA | NA |
| Sotagliflozin:Placebo_or_Control | 2 | -1.552096049 | -1.552096049 | NA | NA | NA | NA | NA |

**Tab. S7C: inconsistency within the primary outcome: subgroup of Alzheimer’s disease events**

| Comparison | No.Studies | NMA | Direct | Indirect | Difference | Diff_95CI_lower | Diff_95CI_upper | *p* value |
| --- | --- | --- | --- | --- | --- | --- | --- | --- |
| Albiglutide:Dapagliflozin | 0 | -0.456647921 | NA | -0.456647921 | NA | NA | NA | NA |
| Albiglutide:Dulaglutide | 0 | 1.7905362 | NA | 1.7905362 | NA | NA | NA | NA |
| Albiglutide:Empagliflozin | 0 | 0.989690607 | NA | 0.989690607 | NA | NA | NA | NA |
| Albiglutide:Ertugliflozin_high_dosage | 0 | 0.000152122 | NA | 0.000152122 | NA | NA | NA | NA |
| Albiglutide:Ertugliflozin_low_dosage | 0 | -0.000212044 | NA | -0.000212044 | NA | NA | NA | NA |
| Albiglutide:Exenatide | 0 | -0.851723585 | NA | -0.851723585 | NA | NA | NA | NA |
| Albiglutide:Liraglutide | 0 | -0.512322602 | NA | -0.512322602 | NA | NA | NA | NA |
| Albiglutide:Placebo_or_Control | 1 | 1.098400245 | 1.098400245 | NA | NA | NA | NA | NA |
| Dapagliflozin:Dulaglutide | 0 | 2.247184122 | NA | 2.247184122 | NA | NA | NA | NA |
| Dapagliflozin:Empagliflozin | 0 | 1.446338528 | NA | 1.446338528 | NA | NA | NA | NA |
| Dapagliflozin:Ertugliflozin_high_dosage | 0 | 0.456800043 | NA | 0.456800043 | NA | NA | NA | NA |
| Dapagliflozin:Ertugliflozin_low_dosage | 0 | 0.456435877 | NA | 0.456435877 | NA | NA | NA | NA |
| Dapagliflozin:Exenatide | 0 | -0.395075663 | NA | -0.395075663 | NA | NA | NA | NA |
| Dapagliflozin:Liraglutide | 0 | -0.055674681 | NA | -0.055674681 | NA | NA | NA | NA |
| Dapagliflozin:Placebo_or_Control | 2 | 1.555048166 | 1.555048166 | NA | NA | NA | NA | NA |
| Dulaglutide:Empagliflozin | 0 | -0.800845594 | NA | -0.800845594 | NA | NA | NA | NA |
| Dulaglutide:Ertugliflozin_high_dosage | 0 | -1.790384078 | NA | -1.790384078 | NA | NA | NA | NA |
| Dulaglutide:Ertugliflozin_low_dosage | 0 | -1.790748245 | NA | -1.790748245 | NA | NA | NA | NA |
| Dulaglutide:Exenatide | 0 | -2.642259785 | NA | -2.642259785 | NA | NA | NA | NA |
| Dulaglutide:Liraglutide | 0 | -2.302858803 | NA | -2.302858803 | NA | NA | NA | NA |
| Dulaglutide:Placebo_or_Control | 1 | -0.692135956 | -0.692135956 | NA | NA | NA | NA | NA |
| Empagliflozin:Ertugliflozin_high_dosage | 0 | -0.989538485 | NA | -0.989538485 | NA | NA | NA | NA |
| Empagliflozin:Ertugliflozin_low_dosage | 0 | -0.989902651 | NA | -0.989902651 | NA | NA | NA | NA |
| Empagliflozin:Exenatide | 0 | -1.841414191 | NA | -1.841414191 | NA | NA | NA | NA |
| Empagliflozin:Liraglutide | 0 | -1.502013209 | NA | -1.502013209 | NA | NA | NA | NA |
| Empagliflozin:Placebo_or_Control | 3 | 0.108709638 | 0.108709638 | NA | NA | NA | NA | NA |
| Ertugliflozin_high_dosage:Ertugliflozin_low_dosage | 1 | -0.000364166 | -0.000364166 | NA | NA | NA | NA | NA |
| Ertugliflozin_high_dosage:Exenatide | 0 | -0.851875707 | NA | -0.851875707 | NA | NA | NA | NA |
| Ertugliflozin_high_dosage:Liraglutide | 0 | -0.512474724 | NA | -0.512474724 | NA | NA | NA | NA |
| Ertugliflozin_high_dosage:Placebo_or_Control | 1 | 1.098248123 | 1.098248123 | NA | NA | NA | NA | NA |
| Ertugliflozin_low_dosage:Exenatide | 0 | -0.851511541 | NA | -0.851511541 | NA | NA | NA | NA |
| Ertugliflozin_low_dosage:Liraglutide | 0 | -0.512110558 | NA | -0.512110558 | NA | NA | NA | NA |
| Ertugliflozin_low_dosage:Placebo_or_Control | 1 | 1.098612289 | 1.098612289 | NA | NA | NA | NA | NA |
| Exenatide:Liraglutide | 0 | 0.339400983 | NA | 0.339400983 | NA | NA | NA | NA |
| Exenatide:Placebo_or_Control | 1 | 1.950123829 | 1.950123829 | NA | NA | NA | NA | NA |
| Liraglutide:Placebo_or_Control | 1 | 1.610722847 | 1.610722847 | NA | NA | NA | NA | NA |

**Tab. S7D: inconsistency within the primary outcome: subgroup of dementia of Lewy body events**

| Comparison | No.Studies | NMA | Direct | Indirect | Difference | Diff_95CI_lower | Diff_95CI_upper | *p* value |
| --- | --- | --- | --- | --- | --- | --- | --- | --- |
| Dapagliflozin:Liraglutide | 0 | 2.196115516 | NA | 2.196115516 | NA | NA | NA | NA |
| Dapagliflozin:Oral_semaglutide | 0 | 2.19738645 | NA | 2.19738645 | NA | NA | NA | NA |
| Dapagliflozin:Placebo_or_Control | 1 | 1.098145626 | 1.098145626 | NA | NA | NA | NA | NA |
| Liraglutide:Oral_semaglutide | 0 | 0.001270934 | NA | 0.001270934 | NA | NA | NA | NA |
| Liraglutide:Placebo_or_Control | 1 | -1.09796989 | -1.09796989 | NA | NA | NA | NA | NA |
| Oral_semaglutide:Placebo_or_Control | 1 | -1.099240824 | -1.099240824 | NA | NA | NA | NA | NA |

**Tab. S7E: inconsistency within the primary outcome: subgroup of multiple sclerosis events**

| Comparison | No.Studies | NMA | Direct | Indirect | Difference | Diff_95CI_lower | Diff_95CI_upper | *p* value |
| --- | --- | --- | --- | --- | --- | --- | --- | --- |
| Dapagliflozin:Exenatide | 0 | -0.004134812 | NA | -0.004134812 | NA | NA | NA | NA |
| Dapagliflozin:Inject_semaglutide | 0 | -0.000465333 | NA | -0.000465333 | NA | NA | NA | NA |
| Dapagliflozin:Lixisenatide | 0 | -2.198349649 | NA | -2.198349649 | NA | NA | NA | NA |
| Dapagliflozin:Placebo_or_Control | 1 | -1.099077621 | -1.099077621 | NA | NA | NA | NA | NA |
| Exenatide:Inject_semaglutide | 0 | 0.00366948 | NA | 0.00366948 | NA | NA | NA | NA |
| Exenatide:Lixisenatide | 0 | -2.194214837 | NA | -2.194214837 | NA | NA | NA | NA |
| Exenatide:Placebo_or_Control | 1 | -1.094942809 | -1.094942809 | NA | NA | NA | NA | NA |
| Inject_semaglutide:Lixisenatide | 0 | -2.197884317 | NA | -2.197884317 | NA | NA | NA | NA |
| Inject_semaglutide:Placebo_or_Control | 1 | -1.098612289 | -1.098612289 | NA | NA | NA | NA | NA |
| Lixisenatide:Placebo_or_Control | 1 | 1.099272028 | 1.099272028 | NA | NA | NA | NA | NA |

**Tab. S7F: inconsistency within the primary outcome: subgroup of amyotrophic lateral sclerosis events**

| Comparison | No.Studies | NMA | Direct | Indirect | Difference | Diff_95CI_lower | Diff_95CI_upper | *p* value |
| --- | --- | --- | --- | --- | --- | --- | --- | --- |
| Canagliflozin:Dapagliflozin | 0 | 0.405181728 | NA | 0.405181728 | NA | NA | NA | NA |
| Canagliflozin:Dulaglutide | 0 | 2.19211871 | NA | 2.19211871 | NA | NA | NA | NA |
| Canagliflozin:Ertugliflozin | 0 | -0.00059364 | NA | -0.00059364 | NA | NA | NA | NA |
| Canagliflozin:Lixisenatide | 0 | 1.50321062 | NA | 1.50321062 | NA | NA | NA | NA |
| Canagliflozin:Placebo_or_Control | 1 | 0.404598332 | 0.404598332 | NA | NA | NA | NA | NA |
| Dapagliflozin:Dulaglutide | 0 | 1.786936982 | NA | 1.786936982 | NA | NA | NA | NA |
| Dapagliflozin:Ertugliflozin | 0 | -0.405775368 | NA | -0.405775368 | NA | NA | NA | NA |
| Dapagliflozin:Lixisenatide | 0 | 1.098028892 | NA | 1.098028892 | NA | NA | NA | NA |
| Dapagliflozin:Placebo_or_Control | 1 | -0.000583397 | -0.000583397 | NA | NA | NA | NA | NA |
| Dulaglutide:Ertugliflozin | 0 | -2.19271235 | NA | -2.19271235 | NA | NA | NA | NA |
| Dulaglutide:Lixisenatide | 0 | -0.68890809 | NA | -0.68890809 | NA | NA | NA | NA |
| Dulaglutide:Placebo_or_Control | 1 | -1.787520379 | -1.787520379 | NA | NA | NA | NA | NA |
| Ertugliflozin:Lixisenatide | 0 | 1.50380426 | NA | 1.50380426 | NA | NA | NA | NA |
| Ertugliflozin:Placebo_or_Control | 1 | 0.405191971 | 0.405191971 | NA | NA | NA | NA | NA |
| Lixisenatide:Placebo_or_Control | 1 | -1.098612289 | -1.098612289 | NA | NA | NA | NA | NA |

**Tab. S7G: inconsistency within the safety profile: drop-out rate**

| Comparison | No.Studies | NMA | Direct | Indirect | Difference | Diff_95CI_lower | Diff_95CI_upper | *p* value |
| --- | --- | --- | --- | --- | --- | --- | --- | --- |
| Albiglutide:Canagliflozin_high_dosage | 0 | 0.349949731 | NA | 0.349949731 | NA | NA | NA | NA |
| Albiglutide:Canagliflozin_low_dosage | 0 | 0.218330869 | NA | 0.218330869 | NA | NA | NA | NA |
| Albiglutide:Dapagliflozin | 0 | -0.02142977 | NA | -0.02142977 | NA | NA | NA | NA |
| Albiglutide:Dulaglutide | 0 | -0.285177014 | NA | -0.285177014 | NA | NA | NA | NA |
| Albiglutide:Empagliflozin_high_dosage | 0 | 0.01535198 | NA | 0.01535198 | NA | NA | NA | NA |
| Albiglutide:Empagliflozin_low_dosage | 0 | -0.060953037 | NA | -0.060953037 | NA | NA | NA | NA |
| Albiglutide:Ertugliflozin_high_dosage | 0 | -0.16617923 | NA | -0.16617923 | NA | NA | NA | NA |
| Albiglutide:Ertugliflozin_low_dosage | 0 | -0.110124781 | NA | -0.110124781 | NA | NA | NA | NA |
| Albiglutide:Exenatide | 0 | -0.164926198 | NA | -0.164926198 | NA | NA | NA | NA |
| Albiglutide:Inject_semaglutide_low_dosage | 0 | 0.160670428 | NA | 0.160670428 | NA | NA | NA | NA |
| Albiglutide:Inject_semaglutide_high_dosage | 0 | 0.400600198 | NA | 0.400600198 | NA | NA | NA | NA |
| Albiglutide:Liraglutide | 0 | -0.067314577 | NA | -0.067314577 | NA | NA | NA | NA |
| Albiglutide:Lixisenatide | 0 | -0.157055348 | NA | -0.157055348 | NA | NA | NA | NA |
| Albiglutide:Oral_semaglutide | 0 | -0.022962333 | NA | -0.022962333 | NA | NA | NA | NA |
| Albiglutide:Placebo_or_Control | 1 | -0.20528389 | -0.20528389 | NA | NA | NA | NA | NA |
| Albiglutide:Sotagliflozin | 0 | 0.44667799 | NA | 0.44667799 | NA | NA | NA | NA |
| Canagliflozin_high_dosage:Canagliflozin_low_dosage | 1 | -0.131618862 | -0.145934915 | 0.102320754 | -0.248255669 | -1.875281945 | 1.378770607 | 0.764897224 |
| Canagliflozin_high_dosage:Dapagliflozin | 0 | -0.371379501 | NA | -0.371379501 | NA | NA | NA | NA |
| Canagliflozin_high_dosage:Dulaglutide | 0 | -0.635126745 | NA | -0.635126745 | NA | NA | NA | NA |
| Canagliflozin_high_dosage:Empagliflozin_high_dosage | 0 | -0.334597751 | NA | -0.334597751 | NA | NA | NA | NA |
| Canagliflozin_high_dosage:Empagliflozin_low_dosage | 0 | -0.410902769 | NA | -0.410902769 | NA | NA | NA | NA |
| Canagliflozin_high_dosage:Ertugliflozin_high_dosage | 0 | -0.516128961 | NA | -0.516128961 | NA | NA | NA | NA |
| Canagliflozin_high_dosage:Ertugliflozin_low_dosage | 0 | -0.460074513 | NA | -0.460074513 | NA | NA | NA | NA |
| Canagliflozin_high_dosage:Exenatide | 0 | -0.514875929 | NA | -0.514875929 | NA | NA | NA | NA |
| Canagliflozin_high_dosage:Inject_semaglutide_low_dosage | 0 | -0.189279304 | NA | -0.189279304 | NA | NA | NA | NA |
| Canagliflozin_high_dosage:Inject_semaglutide_high_dosage | 0 | 0.050650467 | NA | 0.050650467 | NA | NA | NA | NA |
| Canagliflozin_high_dosage:Liraglutide | 0 | -0.417264308 | NA | -0.417264308 | NA | NA | NA | NA |
| Canagliflozin_high_dosage:Lixisenatide | 0 | -0.50700508 | NA | -0.50700508 | NA | NA | NA | NA |
| Canagliflozin_high_dosage:Oral_semaglutide | 0 | -0.372912064 | NA | -0.372912064 | NA | NA | NA | NA |
| Canagliflozin_high_dosage:Placebo_or_Control | 1 | -0.555233621 | -0.543144477 | -0.815103505 | 0.271959028 | -1.510415114 | 2.05433317 | 0.764897224 |
| Canagliflozin_high_dosage:Sotagliflozin | 0 | 0.096728259 | NA | 0.096728259 | NA | NA | NA | NA |
| Canagliflozin_low_dosage:Dapagliflozin | 0 | -0.239760639 | NA | -0.239760639 | NA | NA | NA | NA |
| Canagliflozin_low_dosage:Dulaglutide | 0 | -0.503507883 | NA | -0.503507883 | NA | NA | NA | NA |
| Canagliflozin_low_dosage:Empagliflozin_high_dosage | 0 | -0.202978889 | NA | -0.202978889 | NA | NA | NA | NA |
| Canagliflozin_low_dosage:Empagliflozin_low_dosage | 0 | -0.279283907 | NA | -0.279283907 | NA | NA | NA | NA |
| Canagliflozin_low_dosage:Ertugliflozin_high_dosage | 0 | -0.384510099 | NA | -0.384510099 | NA | NA | NA | NA |
| Canagliflozin_low_dosage:Ertugliflozin_low_dosage | 0 | -0.32845565 | NA | -0.32845565 | NA | NA | NA | NA |
| Canagliflozin_low_dosage:Exenatide | 0 | -0.383257067 | NA | -0.383257067 | NA | NA | NA | NA |
| Canagliflozin_low_dosage:Inject_semaglutide_low_dosage | 0 | -0.057660442 | NA | -0.057660442 | NA | NA | NA | NA |
| Canagliflozin_low_dosage:Inject_semaglutide_high_dosage | 0 | 0.182269329 | NA | 0.182269329 | NA | NA | NA | NA |
| Canagliflozin_low_dosage:Liraglutide | 0 | -0.285645446 | NA | -0.285645446 | NA | NA | NA | NA |
| Canagliflozin_low_dosage:Lixisenatide | 0 | -0.375386218 | NA | -0.375386218 | NA | NA | NA | NA |
| Canagliflozin_low_dosage:Oral_semaglutide | 0 | -0.241293202 | NA | -0.241293202 | NA | NA | NA | NA |
| Canagliflozin_low_dosage:Placebo_or_Control | 2 | -0.423614759 | -0.423614759 | NA | NA | NA | NA | NA |
| Canagliflozin_low_dosage:Sotagliflozin | 0 | 0.228347121 | NA | 0.228347121 | NA | NA | NA | NA |
| Dapagliflozin:Dulaglutide | 0 | -0.263747244 | NA | -0.263747244 | NA | NA | NA | NA |
| Dapagliflozin:Empagliflozin_high_dosage | 0 | 0.03678175 | NA | 0.03678175 | NA | NA | NA | NA |
| Dapagliflozin:Empagliflozin_low_dosage | 0 | -0.039523268 | NA | -0.039523268 | NA | NA | NA | NA |
| Dapagliflozin:Ertugliflozin_high_dosage | 0 | -0.14474946 | NA | -0.14474946 | NA | NA | NA | NA |
| Dapagliflozin:Ertugliflozin_low_dosage | 0 | -0.088695011 | NA | -0.088695011 | NA | NA | NA | NA |
| Dapagliflozin:Exenatide | 0 | -0.143496428 | NA | -0.143496428 | NA | NA | NA | NA |
| Dapagliflozin:Inject_semaglutide_low_dosage | 0 | 0.182100197 | NA | 0.182100197 | NA | NA | NA | NA |
| Dapagliflozin:Inject_semaglutide_high_dosage | 0 | 0.422029968 | NA | 0.422029968 | NA | NA | NA | NA |
| Dapagliflozin:Liraglutide | 0 | -0.045884807 | NA | -0.045884807 | NA | NA | NA | NA |
| Dapagliflozin:Lixisenatide | 0 | -0.135625579 | NA | -0.135625579 | NA | NA | NA | NA |
| Dapagliflozin:Oral_semaglutide | 0 | -0.001532563 | NA | -0.001532563 | NA | NA | NA | NA |
| Dapagliflozin:Placebo_or_Control | 4 | -0.18385412 | -0.18385412 | NA | NA | NA | NA | NA |
| Dapagliflozin:Sotagliflozin | 0 | 0.46810776 | NA | 0.46810776 | NA | NA | NA | NA |
| Dulaglutide:Empagliflozin_high_dosage | 0 | 0.300528994 | NA | 0.300528994 | NA | NA | NA | NA |
| Dulaglutide:Empagliflozin_low_dosage | 0 | 0.224223976 | NA | 0.224223976 | NA | NA | NA | NA |
| Dulaglutide:Ertugliflozin_high_dosage | 0 | 0.118997784 | NA | 0.118997784 | NA | NA | NA | NA |
| Dulaglutide:Ertugliflozin_low_dosage | 0 | 0.175052233 | NA | 0.175052233 | NA | NA | NA | NA |
| Dulaglutide:Exenatide | 0 | 0.120250816 | NA | 0.120250816 | NA | NA | NA | NA |
| Dulaglutide:Inject_semaglutide_low_dosage | 0 | 0.445847441 | NA | 0.445847441 | NA | NA | NA | NA |
| Dulaglutide:Inject_semaglutide_high_dosage | 0 | 0.685777212 | NA | 0.685777212 | NA | NA | NA | NA |
| Dulaglutide:Liraglutide | 0 | 0.217862437 | NA | 0.217862437 | NA | NA | NA | NA |
| Dulaglutide:Lixisenatide | 0 | 0.128121665 | NA | 0.128121665 | NA | NA | NA | NA |
| Dulaglutide:Oral_semaglutide | 0 | 0.262214681 | NA | 0.262214681 | NA | NA | NA | NA |
| Dulaglutide:Placebo_or_Control | 2 | 0.079893124 | 0.079893124 | NA | NA | NA | NA | NA |
| Dulaglutide:Sotagliflozin | 0 | 0.731855004 | NA | 0.731855004 | NA | NA | NA | NA |
| Empagliflozin_high_dosage:Empagliflozin_low_dosage | 2 | -0.076305018 | -0.008834121 | -0.408845813 | 0.400011692 | -0.292454157 | 1.092477541 | 0.257551164 |
| Empagliflozin_high_dosage:Ertugliflozin_high_dosage | 0 | -0.18153121 | NA | -0.18153121 | NA | NA | NA | NA |
| Empagliflozin_high_dosage:Ertugliflozin_low_dosage | 0 | -0.125476761 | NA | -0.125476761 | NA | NA | NA | NA |
| Empagliflozin_high_dosage:Exenatide | 0 | -0.180278178 | NA | -0.180278178 | NA | NA | NA | NA |
| Empagliflozin_high_dosage:Inject_semaglutide_low_dosage | 0 | 0.145318447 | NA | 0.145318447 | NA | NA | NA | NA |
| Empagliflozin_high_dosage:Inject_semaglutide_high_dosage | 0 | 0.385248218 | NA | 0.385248218 | NA | NA | NA | NA |
| Empagliflozin_high_dosage:Liraglutide | 0 | -0.082666557 | NA | -0.082666557 | NA | NA | NA | NA |
| Empagliflozin_high_dosage:Lixisenatide | 0 | -0.172407329 | NA | -0.172407329 | NA | NA | NA | NA |
| Empagliflozin_high_dosage:Oral_semaglutide | 0 | -0.038314313 | NA | -0.038314313 | NA | NA | NA | NA |
| Empagliflozin_high_dosage:Placebo_or_Control | 2 | -0.22063587 | -0.296278522 | 0.136269901 | -0.432548423 | -1.115718379 | 0.250621534 | 0.214624452 |
| Empagliflozin_high_dosage:Sotagliflozin | 0 | 0.43132601 | NA | 0.43132601 | NA | NA | NA | NA |
| Empagliflozin_low_dosage:Ertugliflozin_high_dosage | 0 | -0.105226192 | NA | -0.105226192 | NA | NA | NA | NA |
| Empagliflozin_low_dosage:Ertugliflozin_low_dosage | 0 | -0.049171744 | NA | -0.049171744 | NA | NA | NA | NA |
| Empagliflozin_low_dosage:Exenatide | 0 | -0.10397316 | NA | -0.10397316 | NA | NA | NA | NA |
| Empagliflozin_low_dosage:Inject_semaglutide_low_dosage | 0 | 0.221623465 | NA | 0.221623465 | NA | NA | NA | NA |
| Empagliflozin_low_dosage:Inject_semaglutide_high_dosage | 0 | 0.461553236 | NA | 0.461553236 | NA | NA | NA | NA |
| Empagliflozin_low_dosage:Liraglutide | 0 | -0.00636154 | NA | -0.00636154 | NA | NA | NA | NA |
| Empagliflozin_low_dosage:Lixisenatide | 0 | -0.096102311 | NA | -0.096102311 | NA | NA | NA | NA |
| Empagliflozin_low_dosage:Oral_semaglutide | 0 | 0.037990704 | NA | 0.037990704 | NA | NA | NA | NA |
| Empagliflozin_low_dosage:Placebo_or_Control | 4 | -0.144330852 | -0.140486828 | -3.221084538 | 3.080597709 | -1.508451819 | 7.669647238 | 0.188271235 |
| Empagliflozin_low_dosage:Sotagliflozin | 0 | 0.507631028 | NA | 0.507631028 | NA | NA | NA | NA |
| Ertugliflozin_high_dosage:Ertugliflozin_low_dosage | 1 | 0.056054448 | 0.056054448 | NA | NA | NA | NA | NA |
| Ertugliflozin_high_dosage:Exenatide | 0 | 0.001253032 | NA | 0.001253032 | NA | NA | NA | NA |
| Ertugliflozin_high_dosage:Inject_semaglutide_low_dosage | 0 | 0.326849657 | NA | 0.326849657 | NA | NA | NA | NA |
| Ertugliflozin_high_dosage:Inject_semaglutide_high_dosage | 0 | 0.566779428 | NA | 0.566779428 | NA | NA | NA | NA |
| Ertugliflozin_high_dosage:Liraglutide | 0 | 0.098864653 | NA | 0.098864653 | NA | NA | NA | NA |
| Ertugliflozin_high_dosage:Lixisenatide | 0 | 0.009123881 | NA | 0.009123881 | NA | NA | NA | NA |
| Ertugliflozin_high_dosage:Oral_semaglutide | 0 | 0.143216897 | NA | 0.143216897 | NA | NA | NA | NA |
| Ertugliflozin_high_dosage:Placebo_or_Control | 1 | -0.03910466 | -0.03910466 | NA | NA | NA | NA | NA |
| Ertugliflozin_high_dosage:Sotagliflozin | 0 | 0.61285722 | NA | 0.61285722 | NA | NA | NA | NA |
| Ertugliflozin_low_dosage:Exenatide | 0 | -0.054801417 | NA | -0.054801417 | NA | NA | NA | NA |
| Ertugliflozin_low_dosage:Inject_semaglutide_low_dosage | 0 | 0.270795209 | NA | 0.270795209 | NA | NA | NA | NA |
| Ertugliflozin_low_dosage:Inject_semaglutide_high_dosage | 0 | 0.51072498 | NA | 0.51072498 | NA | NA | NA | NA |
| Ertugliflozin_low_dosage:Liraglutide | 0 | 0.042810204 | NA | 0.042810204 | NA | NA | NA | NA |
| Ertugliflozin_low_dosage:Lixisenatide | 0 | -0.046930567 | NA | -0.046930567 | NA | NA | NA | NA |
| Ertugliflozin_low_dosage:Oral_semaglutide | 0 | 0.087162448 | NA | 0.087162448 | NA | NA | NA | NA |
| Ertugliflozin_low_dosage:Placebo_or_Control | 1 | -0.095159109 | -0.095159109 | NA | NA | NA | NA | NA |
| Ertugliflozin_low_dosage:Sotagliflozin | 0 | 0.556802771 | NA | 0.556802771 | NA | NA | NA | NA |
| Exenatide:Inject_semaglutide_low_dosage | 0 | 0.325596626 | NA | 0.325596626 | NA | NA | NA | NA |
| Exenatide:Inject_semaglutide_high_dosage | 0 | 0.565526396 | NA | 0.565526396 | NA | NA | NA | NA |
| Exenatide:Liraglutide | 0 | 0.097611621 | NA | 0.097611621 | NA | NA | NA | NA |
| Exenatide:Lixisenatide | 0 | 0.00787085 | NA | 0.00787085 | NA | NA | NA | NA |
| Exenatide:Oral_semaglutide | 0 | 0.141963865 | NA | 0.141963865 | NA | NA | NA | NA |
| Exenatide:Placebo_or_Control | 2 | -0.040357692 | -0.040357692 | NA | NA | NA | NA | NA |
| Exenatide:Sotagliflozin | 0 | 0.611604188 | NA | 0.611604188 | NA | NA | NA | NA |
| Inject_semaglutide_low_dosage:Inject_semaglutide_high_dosage | 1 | 0.239929771 | 0.239929771 | NA | NA | NA | NA | NA |
| Inject_semaglutide_low_dosage:Liraglutide | 0 | -0.227985005 | NA | -0.227985005 | NA | NA | NA | NA |
| Inject_semaglutide_low_dosage:Lixisenatide | 0 | -0.317725776 | NA | -0.317725776 | NA | NA | NA | NA |
| Inject_semaglutide_low_dosage:Oral_semaglutide | 0 | -0.183632761 | NA | -0.183632761 | NA | NA | NA | NA |
| Inject_semaglutide_low_dosage:Placebo_or_Control | 1 | -0.365954318 | -0.365954318 | NA | NA | NA | NA | NA |
| Inject_semaglutide_low_dosage:Sotagliflozin | 0 | 0.286007563 | NA | 0.286007563 | NA | NA | NA | NA |
| Inject_semaglutide_high_dosage:Liraglutide | 0 | -0.467914776 | NA | -0.467914776 | NA | NA | NA | NA |
| Inject_semaglutide_high_dosage:Lixisenatide | 0 | -0.557655547 | NA | -0.557655547 | NA | NA | NA | NA |
| Inject_semaglutide_high_dosage:Oral_semaglutide | 0 | -0.423562532 | NA | -0.423562532 | NA | NA | NA | NA |
| Inject_semaglutide_high_dosage:Placebo_or_Control | 1 | -0.605884088 | -0.605884088 | NA | NA | NA | NA | NA |
| Inject_semaglutide_high_dosage:Sotagliflozin | 0 | 0.046077792 | NA | 0.046077792 | NA | NA | NA | NA |
| Liraglutide:Lixisenatide | 0 | -0.089740771 | NA | -0.089740771 | NA | NA | NA | NA |
| Liraglutide:Oral_semaglutide | 0 | 0.044352244 | NA | 0.044352244 | NA | NA | NA | NA |
| Liraglutide:Placebo_or_Control | 1 | -0.137969313 | -0.137969313 | NA | NA | NA | NA | NA |
| Liraglutide:Sotagliflozin | 0 | 0.513992567 | NA | 0.513992567 | NA | NA | NA | NA |
| Lixisenatide:Oral_semaglutide | 0 | 0.134093015 | NA | 0.134093015 | NA | NA | NA | NA |
| Lixisenatide:Placebo_or_Control | 1 | -0.048228542 | -0.048228542 | NA | NA | NA | NA | NA |
| Lixisenatide:Sotagliflozin | 0 | 0.603733339 | NA | 0.603733339 | NA | NA | NA | NA |
| Oral_semaglutide:Placebo_or_Control | 1 | -0.182321557 | -0.182321557 | NA | NA | NA | NA | NA |
| Oral_semaglutide:Sotagliflozin | 0 | 0.469640323 | NA | 0.469640323 | NA | NA | NA | NA |
| Sotagliflozin:Placebo_or_Control | 2 | -0.65196188 | -0.65196188 | NA | NA | NA | NA | NA |

*Dosage definition: Canagliflozin (Low: 100mg, and High: 300mg); Ertugliflozin (Low: 5mg, and High: 15mg); Injectable semaglutide (Low: 0.5mg, and High: 1.0mg); Empagliflozin (Low: 1-10mg, and High: 25-50mg).*

*Abbreviation: 95%CIs: 95% confidence intervals; GLP-1 agonist: glucagon-like peptide-1 agonist; NA: not applicable; NMA: network meta-analysis; OR: odds ratio; RCT: randomized controlled trial; SGLT2 inhibitor: sodium–glucose cotransporter 2 inhibitor*
